# Supplementary material for: Acute sore throat and Fusobacterium necrophorum in primary healthcare: a systematic review and meta-analysis
Source: BMJ Open. 2021 Jun 4;11(6):e042816. doi: 10.1136/bmjopen-2020-042816 (PMC8183226; doi:10.1136/bmjopen-2020-042816)
Supplement: Supplementary data [file bmjopen-2020-042816supp001.pdf]

Acute Sore Throat and Fusobacterium Necrophorum in PHC – A Systematic Review and Meta-analysis - Appendix 1 and 2
Version: (11 January 2021)

Appendix 1: Search strings for PubMed and PROSPERO

PubMed:

(("pharyngitis"[MH] OR "pharyngitis"[TIAB] OR "Lemierre Syndrome"[MH] OR "Lemierre Syndrome"[TIAB] OR "Necrobacillosis"[TIAB] OR "pharyngotonsillitis "[TIAB] OR "tonsillitits"[TIAB] OR "throat"[TIAB] OR "epidemiology"[TIAB])

AND ("fusobacterium necrophorum"[MH] OR "fusobacterium"[TIAB]))

NOT "Case Reports"[pt]

Scopus:

(TITLE-ABS-KEY ((pharyngitis OR "Lemierre Syndrome" OR necrobacillosis OR pharyngotonsillitis OR tonsillitis OR "Peritonsillar Abscess" OR throat OR epidemiology))

AND TITLE-ABS-KEY (fusobacterium))

AND DOCTYPE (ar)

AND (LIMIT-TO (SUBJAREA , "MEDI ") OR LIMIT-TO (SUBJAREA , " IMMU ")

OR LIMIT-TO (SUBJAREA , " BIO "))

AND (LIMIT-TO (LANGUAGE , "English "))

AND (EXCLUDE (EXACTKEYWORD , "Case Report"))

Malmberg S, *et al. BMJ Open* 2022; 11:e042816. doi: 10.1136/bmjopen-2020-042816

BMJ Publishing Group Limited (BMJ) disclaims all liability and responsibility arising from any reliance placed on this supplemental material which has been supplied by the author(s)

Acute Sore Throat and Fusobacterium Necrophorum in PHC – A Systematic Review and Meta-analysis - Appendix 1 and 2
Version: (11 January 2021)

Appendix 2: Formulae for calculating P-EPV

Formula for the point estimate of P-EPV:

$$P(\mathrm{D}^+ | \mathrm{S}^+\mathrm{T}^+) = 1 - \left(\frac{\dfrac{\mathrm{Sen}}{P(\mathrm{T}^+ | \mathrm{S}^+)} - 1}{\dfrac{\mathrm{Sen}}{P(\mathrm{T}^+ | \mathrm{S}^+\mathrm{D}^-)} - 1} \right)$$

Formula for the confidence interval of P-EPV:

$$\left(1 - \left(\frac{\dfrac{\mathrm{Sen}}{a} - 1}{\dfrac{\mathrm{Sen}}{d} - 1} \right) \right), \left(1 - \left(\frac{\dfrac{\mathrm{Sen}}{b} - 1}{\dfrac{\mathrm{Sen}}{c} - 1} \right) \right)$$

$$a = p(\mathrm{T}^+ | \mathrm{S}^+) - \left(Z_{\alpha/4} \times \sqrt{\frac{p(\mathrm{T}^+ | \mathrm{S}^+) \times \left(1 - p(\mathrm{T}^+ | \mathrm{S}^+)\right)}{\#(\mathrm{S}^+)}} \right)$$

$$b = p(\mathrm{T}^+ | \mathrm{S}^+) + \left(Z_{\alpha/4} \times \sqrt{\frac{p(\mathrm{T}^+ | \mathrm{S}^+) \times \left(1 - p(\mathrm{T}^+ | \mathrm{S}^+)\right)}{\#(\mathrm{S}^+)}} \right)$$

$$c = p(\mathrm{T}^+ | \mathrm{S}^+\mathrm{D}^-) - \left(Z_{\alpha/4} \times \theta \times \sqrt{\frac{p(\mathrm{T}^+ | \mathrm{S}^-) \times \left(1 - p(\mathrm{T}^+ | \mathrm{S}^-)\right)}{\#(\mathrm{S}^-)}} \right)$$

$$d = p(\mathrm{T}^+ | \mathrm{S}^+\mathrm{D}^-) + \left(Z_{\alpha/4} \times \theta \times \sqrt{\frac{p(\mathrm{T}^+ | \mathrm{S}^-) \times \left(1 - p(\mathrm{T}^+ | \mathrm{S}^-)\right)}{\#(\mathrm{S}^-)}} \right)$$

Definitions:
T = Test outcome which may be positive (T^+) or negative (T^-)
S = Symptoms of a sore throat which may be present (S^+) or absent (S^-)
M = The bacteria of interest (FN or GAS) which may be present (M^+) or absent (M^-)
D = Disease (a sore throat caused by FN/GAS) which may be present (D^+) or absent (D^-)
Sensitivity of test to detect bacteria = $\mathrm{Sen} = P(\mathrm{T}^+ | \mathrm{M}^+)$

Setting the width of the confidence interval. For 95%: $Z_{\alpha/4} = Z_{0.05/4} = Z_{0.0125} = 2.24$
An online calculator doing the above: https://science-network.tv/epv-calculator/

Malmberg S, *et al. BMJ Open* 2022; 11:e042816. doi: 10.1136/bmjopen-2020-042816
