# Supplementary material for: Acute sore throat and Fusobacterium necrophorum in primary healthcare: a systematic review and meta-analysis
Source: BMJ Open. 2021 Jun 4;11(6):e042816. doi: 10.1136/bmjopen-2020-042816 (PMC8183226; doi:10.1136/bmjopen-2020-042816)
Supplement: Author's manuscript [file bmjopen-2020-042816.draft_revisions.pdf]

BMJ Open is committed to open peer review. As part of this commitment we make the peer review history of every article we publish publicly available.

When an article is published we post the peer reviewers' comments and the authors' responses online. We also post the versions of the paper that were used during peer review. These are the versions that the peer review comments apply to.

The versions of the paper that follow are the versions that were submitted during the peer review process. They are not the versions of record or the final published versions. They should not be cited or distributed as the published version of this manuscript.

BMJ Open is an open access journal and the full, final, typeset and author-corrected version of record of the manuscript is available on our site with no access controls, subscription charges or pay-per-view fees (http://bmjopen.bmj.com).

If you have any questions on BMJ Open's open peer review process please email info.bmjopen@bmj.com

BMJ Open

Acute Sore Throat and Fusobacterium Necrophorum in Primary Health Care – A Systematic Review and Meta-analysis

Journal:	*BMJ Open*
Manuscript ID	bmjopen-2020-042816
Article Type:	Original research
Date Submitted by the Author:	26-Jul-2020
Complete List of Authors:	Malmberg, Stefan; University of Gothenburg Sahlgrenska Academy, School of Public health and Community Medicine<br>Petrén, Susanna; University of Gothenburg Sahlgrenska Academy, School of Public health and Community Medicine<br>Gunnarsson, Ronny; University of Gothenburg Sahlgrenska Academy, School of Public Health and Community Medicine; Vastra Gotalandsregion, Regionhalsan, FoUU<br>Hedin, Katarina; Linköpings universitet Institutionen för medicin och hälsa; Region Jönköpings län, Futurum academy for health and care<br>Sundvall, Pär-Daniel ; Vastra Gotalandsregion, Research and Development Primary Health Care; University of Gothenburg Sahlgrenska Academy, School of Public health and Community Medicine
Keywords:	Epidemiology < INFECTIOUS DISEASES, PRIMARY CARE, INFECTIOUS DISEASES

SCHOLARONE™
Manuscripts

I, the Submitting Author has the right to grant and does grant on behalf of all authors of the Work (as defined in the below author licence), an exclusive licence and/or a non-exclusive licence for contributions from authors who are: i) UK Crown employees; ii) where BMJ has agreed a CC-BY licence shall apply, and/or iii) in accordance with the terms applicable for US Federal Government officers or employees acting as part of their official duties; on a worldwide, perpetual, irrevocable, royalty-free basis to BMJ Publishing Group Ltd ("BMJ") its licensees and where the relevant Journal is co-owned by BMJ to the co-owners of the Journal, to publish the Work in this journal and any other BMJ products and to exploit all rights, as set out in our licence.

The Submitting Author accepts and understands that any supply made under these terms is made by BMJ to the Submitting Author unless you are acting as an employee on behalf of your employer or a postgraduate student of an affiliated institution which is paying any applicable article publishing charge ("APC") for Open Access articles. Where the Submitting Author wishes to make the Work available on an Open Access basis (and intends to pay the relevant APC), the terms of reuse of such Open Access shall be governed by a Creative Commons licence – details of these licences and which Creative Commons licence will apply to this Work are set out in our licence referred to above.

Other than as permitted in any relevant BMJ Author's Self Archiving Policies, I confirm this Work has not been accepted for publication elsewhere, is not being considered for publication elsewhere and does not duplicate material already published. I confirm all authors consent to publication of this Work and authorise the granting of this licence.

For peer review only

**Title**	**Acute Sore Throat and Fusobacterium Necrophorum in Primary Health Care – A Systematic Review and Meta-analysis**
**Authors' name**	Stefan Malmberg[1,2], Susanna Petrén[3], Ronny Gunnarsson[1,4,5], Katarina Hedin[6,7], Pär-Daniel Sundvall[1,4,5]
**Address for each author**	[1] Region Västra Götaland, Research and Development Primary Health Care, Research and Development Centre Södra Älvsborg, Sven Eriksonsplatsen 4, SE-503 38 Borås, Sweden<br>[2] HälsoBrunnen VC, Praktikertjänst AB, Vistvägen 2, 52337 Ulricehamn, Sweden.<br>[3] Närhälsan Högsbo vårdcentral, Markmyntsgatan 14A, 414 80 Gothenburg, Sweden<br>[4] Primary Health Care, Department of Public Health and Community Medicine, Institute of Medicine, Sahlgrenska Academy, University of Gothenburg, Box 454, SE-405 30 Gothenburg Sweden<br>[5] Centre for Antibiotic Resistance Research (CARe) at University of Gothenburg, Gothenburg, Sweden<br>[6] Futurum, Region Jönköping County, Jönköping and Department of Health, Medicine and Caring Sciences, Linköping University, Linköping, Sweden<br>[7] Department of Clinical Sciences in Malmö, Family Medicine, Lund University, Malmö, Sweden
**Authors' names and positions**	Stefan Malmberg MD PhD student, Susanna Petrén MD, Ronny Gunnarsson MD PhD Professor in General Practice, Katarina Hedin MD PhD and Associate Professor in General Practice, Pär-Daniel Sundvall MD PhD
**Corresponding author**	Stefan Malmberg,<br>stefan.malmberg@stfn.se
**Word count**	Abstract: 211<br>Manuscript excluding abstract, references and tables: 3,288

For peer review only

For peer review only - http://bmjopen.bmj.com/site/about/guidelines.xhtml

Acute Sore Throat and Fusobacterium Necrophorum in PHC – A Systematic Review and Meta-analysis
Version: (7/26/2020 8:14:00 AM)

Abstract

Purpose: The objective of this review was to describe and quantify the association between *Fusobacterium necrophorum* (FN) and acute sore throat in primary health care. **Methods**: In this systematic review and meta-analysis, we searched Scopus and PubMed for studies reporting the prevalence of FN in patients attending primary care for a sore throat. Inclusion criteria and methods were specified in advance and published in PROSPERO. The primary outcome was positive etiologic predictive value (P-EPV), quantifying the probability for an association between acute sore throat and findings of FN in the pharynx. For comparison, our secondary outcome was the corresponding P-EPV for Group A Streptococcus (GAS).
Results: PubMed and Scopus yielded 258 and 232 studies, respectively. Removing duplicates and screening the abstracts resulted in 53 studies subsequently read in full text. After a systematic analysis using the SIGN50 quality assessment instrument, four publications qualified for the meta-analysis. For the four studies of medium to high quality, the cumulative P-EPV regarding FN was 64% (95% CI 33-83%). GAS, based on data from the same publications, yielded a positive EPV of 93% (95% CI 83-99%). **Conclusions**: The results indicate that FN may play a role in primary health care patients with an acute sore throat, but the association is much weaker compared to GAS.

For peer review only

For peer review only - http://bmjopen.bmj.com/site/about/guidelines.xhtml

Acute Sore Throat and Fusobacterium Necrophorum in PHC – A Systematic Review and Meta-analysis
Version: (7/26/2020 8:14:00 AM)

Article Summary

Strengths and limitations of this study

- This is the first systematic literature review with meta-analysis utilizing positive etiologic predictive value to quantify the clinical relevance of a finding of *Fusobacterium necrophorum* in patients presenting with an acute uncomplicated sore throat in PHC.
- The positive etiologic predictive value reveals the probability for a true link between *Fusobacterium necrophorum* and the sore throat expressed as a plain percentage between 0-100%.
- A potential limitation is that there were only a few available case-control studies presenting the proportion of FN.

For peer review only

For peer review only - http://bmjopen.bmj.com/site/about/guidelines.xhtml

Introduction

An acute sore throat is a common reason for attending primary health care (PHC).[1-3] Most current guidelines concerning the management of patients with a sore throat focus on Group A *Streptococci* (GAS).[4-6] However, recent studies have indicated that *Fusobacterium necrophorum* (FN) might cause a sore throat, particularly among adolescents.[7-9]

FN is an anaerobic gram-negative bacterium most known for causing the severe disease Lemierre's syndrome, a potentially life-threatening condition which typically begins with a sore throat, and is also an established pathogen in peritonsillar abscess (PTA).[10-12]

The role of FN in the sore throat has been studied in three recent reviews.[7-9] None of the three reviews have taken into consideration the carriage rate of FN in healthy controls, which is of importance when estimating the clinical relevance of FN in patients with an uncomplicated acute sore throat.

This study aimed to estimate the probability for an association between FN and uncomplicated acute sore throat in patients attending PHC, when taking into consideration the carriage rate of FN in healthy controls.

Patient and public involvement

No patient involved.

Methods

The review was conducted in accordance with the Preferred Reporting Items for Systematic Reviews and Meta-Analyses (PRISMA) statement.[13] Inclusion criteria and methods were specified in advance and documented in the review protocol. The initial protocol was

For peer review only - http://bmjopen.bmj.com/site/about/guidelines.xhtml

For peer review only

published beforehand in PROSPERO (International Prospective Register of Systematic Reviews), September 5, 2018 (registration number CRD42018106800).

Search Strategy

PubMed and SCOPUS were searched (17 September 2018) for case-control studies reporting the prevalence of FN in patients with a sore throat and in healthy individuals without any signs of infection. There were no time limitations. The search terms are described in Appendix 1.

Study Selection

All prospective and retrospective case-control studies reporting the prevalence of FN in patients attending a PHC setting for an uncomplicated acute sore throat and in a healthy control group were included. Only studies published in English were considered. Publications were not included if they were case studies, or if they included patients prescribed antibiotics before the throat swab, patients with a concurrent malignant disease, on immunosuppression, having an HIV infection, or patients having another acute infection in addition to a sore throat.

SM performed the first screening to remove duplicates from the two search strategies and, thereafter, to remove obviously irrelevant studies. The remaining studies were carefully screened independently by SM and SP to identify studies that potentially met the inclusion criteria outlined above.

The full texts of these potentially eligible studies were retrieved and independently assessed for eligibility by SM and SP. Any disagreement between them over the eligibility of particular studies was resolved through discussion within the review team.

For peer review only - http://bmjopen.bmj.com/site/about/guidelines.xhtml

The reference lists for each article were screened for additional articles potentially matching the inclusion criteria. Such articles were added to the list of potentially eligible studies for further assessment.

Appraisal of methodological quality

SM and SP independently assessed the risk of bias in the included studies by using the SIGN 50 "Methodology Checklist 4: Case Control Studies".[14] Additional methodological quality characteristics were: type of study, definition of cases, definition of healthy controls, swab method, culture method, and cases and controls collected in the same geographic area and period of time. Disagreements over the risk of bias in particular studies were resolved by discussion within the review team.

Data extraction

A standardized, pre-piloted form was used to extract data from the included studies for assessment of methodological quality and evidence synthesis. Extracted information included: study setting, type of study, definition of cases and classification of these using the Centor criteria if available, definition of healthy controls, swab method, culture method, details of the intervention and control conditions, study methodology, recruitment and study completion rates, outcomes of throat swabs, and information for the assessment of the risk of bias. SM and SP extracted data independently. Discrepancies were identified and resolved through discussion.

Data analysis

For peer review only - http://bmjopen.bmj.com/site/about/guidelines.xhtml
For peer review only

A narrative synthesis was produced for each of the included studies, structured around the study methodology, target population characteristics, outcome, and the assessment of evidence of publication bias.

Studies with a healthy control group and of a high methodological quality were used for the meta-analysis, where the pooled difference in prevalence of FN between cases and healthy controls was compared using chi-square test.

The clinical relevance of any statistical differences between symptomatic patients and healthy controls was further explored by calculating the positive etiologic predictive value (P-EPV)[15]. P-EPV is a method of quantifying the probability of a true link between the symptom (a sore throat) and the finding of FN in the throat while considering the possibility of healthy carriers of FN. P-EPV for FN was, when possible, compared to P-EPV for GAS using data from the same patients and publications.

Results

The PubMed search yielded 258 publications, and the Scopus database query yielded 232 (Figure 1). Removing duplicates and screening the abstracts resulted in 53 studies subsequently read in full text.

Exclusion of publications

Thirty-seven of these 53 articles were not included because they had a different focus, i.e. lab methods [16], or focused on a different category of patients than was the scope of this review: chronically ill patients [17], hospitalized patients [18-23], or patients with a subset of infections such as peritonsillar abscess, Lemierre's syndrome, chronic/recurrent tonsillitis, and intra-abdominal infections [11,24-51]. Four were excluded because they lacked a control group [52-55].

For peer review only - http://bmjopen.bmj.com/site/about/guidelines.xhtml

Discussions in the review team prompted the exclusion of another five articles with methodological limitations in relation to the scope of this review.[56-60]

The article published in 2004 by Aliyu et al.[56] concerned a study where routine throat swabs were analyzed for FN-specific DNA and compared with swabs obtained from healthy adults. The cases were randomly selected in the lab, but it was unclear to what extent they had a sore throat and how these symptoms were registered. The cases included children as young as five months. Hence, it was unclear what kind of patients the routine throat swabs sent to the laboratory represented. Inclusion in the control group required the absence of antibiotic therapy in the preceding two weeks, but not for cases. The swabs from cases were also cultured for GAS, but there was no information about the prevalence of GAS in the control group. The mean age and range differed substantially between cases and controls.

The article published in 2018 by Atkinson et al.[57] presented the results of applying a new laboratory method on swabs from a previously published study [61] and comparing the results to the method that was used initially. The cases included university students complaining of a sore throat, while the controls were asymptomatic students. Only 30 of the 180 control swabs were used in this study, limiting the validity of the comparison between the different lab methods. This limitation is highlighted by the remarkable difference in outcome between the laboratory methods when reviewing cases and controls for both FN and GAS.

A Letter to the Editor published in 2014 by Eaton et al. [58] concerned a project in which all throat swabs received by a microbiology laboratory in one year were cultured for GAS and FN, indicating that the only inclusion criterion was that a throat swab was taken. The laboratory served both PHC and secondary care, while the scope of this study was to focus on uncomplicated acute sore throat in PHC. Clinical details stated on accompanying request forms were used to determine if patients had pharyngitis. Those with either persistent

For peer review only - http://bmjopen.bmj.com/site/about/guidelines.xhtml

or recurrent symptoms were considered to have persistent sore throat syndrome (PSTS), indicating that multiple swabs from the same individual were allowed in the data. There was no information about antibiotic treatment. In conclusion, it was decided not to include the Letter to the Editor by Eaton et al. [58] in this review.

The text published in 2009 by Ludlam et al. [59] described a study design comparing cases from a local GP to controls comprising healthy university students, collecting throat swabs for both groups during the same two-month period. The description of inclusion and exclusion criteria for the cases was limited. The results for GAS, EBV, and FN were not shown for both groups, either in text or in tables. It is unclear whether the controls may become cases (and vice-versa). The supplementary table contained information about antibiotic treatment in the control group, but not for the cases.

The article published in 2018 by Pallon et al. [60] was a follow-up study based on the same initial data already included in a previously published article by Hedin et al. [62], which is included in this review. Therefore, the article published in 2018 by Pallon et al. [60] was not included.

Methodological quality

Of the six studies included in the qualitative analysis, four were high quality prospective studies using well defined cases and controls collected in the same geographical area and time [61-64] (Figure 2) and these were included in the meta-analysis.

Two studies presenting data from cases and controls were of medium quality [65,66] and these were not included in the meta-analysis for the reasons described below.

The Danish study by Jensen et al. 2015 [66] examined the outcome of throat cultures arriving at a microbiologic laboratory. Most of the cases came from patients with 3-4 Centor criteria who had already tested negative with a rapid antigen detection test (RADT) for GAS.

For peer review only - http://bmjopen.bmj.com/site/about/guidelines.xhtml

The control group consisted of subjects having a sore throat with 0-2 Centor criteria or fever or who were screened for carriage of Staphylococcus aureus. None of the controls were screened using the above-mentioned RADT. The cases and controls were, therefore, deemed inappropriate for inclusion in the meta-analysis.

The article published in 2007 by Jensen et al. [65] had similar problems as those described above. The inclusion criteria were somewhat unclear. It appears as if primarily patients with a negative outcome of a RADT for GAS were included as cases. Hence, this study was also not included in the meta-analysis.

Presence of Fusobacterium necrophorum in patients with a sore throat

In high quality articles, FN was detected in 18% of cases with a sore throat and a Centor score of 0-4, compared to 7% in healthy controls ($p<0.00001$, chi-square) (Table 1). The cumulative positive EPV for FN, including patients with 0-4 Centor scores, was 64% (95% CI 33-83%) (Figure 3). In cases with a Centor score of 3-4, FN was detected in 21% ($p<0.00001$, chi-square) (Table 1). The cumulative positive EPV regarding FN for patients with a Centor score of 3-4 was 71% (95% CI 34-88%) (Figure 4).

Fusobacterium necrophorum compared to Group A Streptococcus

When including all cases (Centor score 0-4) in high quality studies also providing data for GAS in the very same patients, the cumulative positive EPV for a finding of GAS was 93% (95% CI 83-99%) (Figure 5). In cases with a Centor score of 3-4, the positive EPV for GAS was 97% (95% CI 91-100%) (Figure 6).

Discussion

For peer review only - http://bmjopen.bmj.com/site/about/guidelines.xhtml

This literature review and meta-analysis showed that the P-EPV for FN (detected by culture or PCR for FN) and acute sore throat was 64% (95% CI 33-83%). The corresponding P-EPV for GAS was 93% (95% CI 83-99%), based on data from the same publications and patients. When limiting the analyses to the groups with Centor score 3-4, the P-EPV for FN was 71% (95% CI 34-88%) and for GAS, 97% (95% CI 91-100%).

Strengths and limitations

A potential limitation is that there were only a few available case-control studies presenting the proportion of FN. However, this study is the first systematic literature review with meta-analysis utilizing P-EPV to quantify the clinical relevance of a finding of FN in patients presenting with an acute uncomplicated sore throat in PHC. As such, it represents the current best understanding of the clinical importance of FN in patients with an uncomplicated acute sore throat.

Using a random effects meta-analysis would have provided odds ratios for harboring FN among cases compared to controls. This type of effect size does not give an easy to understand interpretation to a clinician. The statistical technique we used for meta-analysis, P-EPV, has been used previously[67] and provides a probability for a true link between FN and the symptom of a sore throat. This outcome is a plain percent between 0-100%. This was in our opinion a more clinically useful outcome that can be easily understood by clinicians. P-EPV does not directly take into account between-study variation so it is not a random effect model. We try to compensate for this by presenting our outcome for each individual study as well as a sensitivity analysis where we compare the consequences of combining them differently (Figure 3-6). Furthermore, P-EPV has the ability to tweak the proportion of individuals harboring FN between healthy controls and symptomatic carriers ill from something else like a virus. However, in this review we chose to not tweak this, so we set

For peer review only - http://bmjopen.bmj.com/site/about/guidelines.xhtml
For peer review only

theta to 1.0 meaning that symptomatic carriers harbor FN equally often as healthy controls. Finally, P-EPV allows us to consider the sensitivity of the test to detect FN, something that conventional random effects meta-analysis doesn't. The relatively high prevalence of FN in healthy controls (7.2%) indicates that FN, at least for some patients, is a part of the normal tonsillar flora. The proportion of patients with FN was 18% for Centor score 0-4 and 21% for Centor score 3-4, but the corresponding increase for GAS was from 19% to 35% (Table 1). Subsequently, the difference in the 95% confidence interval for P-EPV between patients having 0-4 versus 3-4 Centor scores was very small for FN, even if there was a marginal increase in the P-EPV. A larger difference would be expected if FN is the main etiologic agent in a relevant proportion of patients.

The P-EPV numbers indicate that FN may play a role as a pathogen in sore throat. However, compared to the results for GAS, the association between FN and sore throat appears to be considerably weaker. Furthermore, the narrow confidence intervals for GAS P-EPV are in contrast to the wide confidence intervals associated with the P-EPV for FN.

The high P-EPV for a finding of GAS in a throat swab is convincing and confirms the already well-established link between GAS and sore throat.[6]

It has been suggested that antibiotic treatment of uncomplicated sore throat caused by FN would be cost-effective if it reduces the incidence of Lemierre's syndrome by at least 20% [25]. However, this has not yet been investigated in a clinical trial, and it is unlikely it ever will be due to the very low incidence of Lemierre's syndrome. Other possible reasons for prescribing antibiotic treatment to patients with an uncomplicated sore throat caused by FN might be to shorten symptom duration or reduce the incidence of PTA. However, neither one has ever been tested in a clinical trial. Hence, although theoretically possible, we still have no proof that antibiotic treatment is beneficial to patients with an uncomplicated sore throat and presence of FN.

For peer review only

For peer review only - http://bmjopen.bmj.com/site/about/guidelines.xhtml

Conclusions and implications

For uncomplicated acute sore throat in PHC, the confidence interval of P-EPV for FN is wide and low compared to the corresponding P-EPV for GAS. There appears to be an association between FN and the uncomplicated acute sore throat in PHC, but it is weak compared to the same association for GAS.

We are not aware of any studies showing that antibiotic treatment has beneficial effects on the duration or severity of symptoms in an FN-associated acute sore throat.

Hence, we do not recommend routinely searching for FN in throat swabs or prescribing antibiotics to patients with a GAS negative uncomplicated acute sore throat in PHC, even when FN is a suspected pathogen.

Future research should focus on randomizing patients with an uncomplicated acute sore throat and presence of FN to treatment with antibiotics or placebo in order to assess whether the treatment is effective to reduce duration and intensity of symptoms, and, more importantly, if complications such as PTA can be prevented. The prevalence of Lemierre's syndrome is so low that any effect of antibiotics on its prevalence most likely would need to be estimated using other designs than a simple clinical trial comparing antibiotics with placebo.

Contributors

RG wa responsible for conception of the idea. The overall design of the study was made by RG, SM, PDS, SP and KH. SM and SP were responsible for assessing the eligibility and extract data from publications. Statistical analysis, interpretation of results and writing of manuscript, was made by all authors (RG, SM, PDS, SP and KH). All authors (RG, SM, PDS, SP and KH) approved the final version of the manuscript.

For peer review only - http://bmjopen.bmj.com/site/about/guidelines.xhtml

Data sharing statement

The raw data files are available from the corresponding author upon request.

Competing interest statement

None to declare.

Acknowledgements

Funding for this project came from the Local Research and Development Council, Södra Älvsborg, Sweden (reference number VGFOUSA-937533). Funding was also received from Research and Development Center for Primary Health Care, Borås, Sweden (reference number VGFOUSA-P-872381).

The authors would like to thank Professor Robert Centor who willingly provided further information from his study "The clinical presentation of Fusobacterium-positive and streptococcal-positive pharyngitis in a university health clinic: a cross-sectional study" [61], enabling us to calculate separate P-EPV for patients with 0-4 and 3-4 Centor criteria.

For peer review only - http://bmjopen.bmj.com/site/about/guidelines.xhtml
For peer review only

References

1. André M, Vernby Å, Odenholt I, et al. Diagnosis-prescribing surveys in 2000, 2002 and 2005 in Swedish general practice: Consultations, diagnosis, diagnostics and treatment choices. *Scandinavian Journal of Infectious Diseases.* 2008(40):648-654.
2. Tyrstrup M, Beckman A, Molstad S, et al. Reduction in antibiotic prescribing for respiratory tract infections in Swedish primary care- a retrospective study of electronic patient records.(Report). *BMC Infectious Diseases.* 2016;16(1).
3. Armstrong GL, Pinner RW. Outpatient visits for infectious diseases in the United States, 1980 through 1996. *Arch Intern Med.* 1999;159(21):2531-2536.
4. Shulman ST, Bisno AL, Clegg HW, et al. Clinical practice guideline for the diagnosis and management of group A streptococcal pharyngitis: 2012 update by the Infectious Diseases Society of America. *Clinical infectious diseases : an official publication of the Infectious Diseases Society of America.* 2012;55(10):e86.
5. Centor RM, Witherspoon JM, Dalton HP, Brody CE, Link K. The diagnosis of strep throat in adults in the emergency room. *Medical decision making : an international journal of the Society for Medical Decision Making.* 1981;1(3):239.
6. Pelucchi C, Grigoryan L, Galeone C, et al. Guideline for the management of acute sore throat. In. Vol 18. Oxford, UK2012:1-28.
7. Marchello C, Ebell MH. Prevalence of Group C Streptococcus and Fusobacterium Necrophorum in Patients With Sore Throat: A Meta-Analysis. *Ann Fam Med.* 2016;14(6):567-574.
8. Klug TE, Rusan M, Fuursted K, Ovesen T, Jorgensen AW. A systematic review of Fusobacterium necrophorum-positive acute tonsillitis: prevalence, methods of detection, patient characteristics, and the usefulness of the Centor score. *European journal of*

For peer review only - http://bmjopen.bmj.com/site/about/guidelines.xhtml

clinical microbiology & infectious diseases : official publication of the European Society of Clinical Microbiology. 2016;35(12):1903-1912.

9. Holm K, Bank S, Nielsen H, Kristensen LH, Prag J, Jensen A. The role of Fusobacterium necrophorum in pharyngotonsillitis - A review. *Anaerobe.* 2016;42:89-97.
10. Riordan T. Human infection with Fusobacterium necrophorum (Necrobacillosis), with a focus on Lemierre's syndrome. *Clinical microbiology reviews.* 2007;20(4):622-659.
11. Wikstén J, Kaltiainen E, Pitkäranta A, Blomgren K. Renewal of peritonsillar abscess: Impact of the bacterial species of the infection and clinical features of the patient—A prospective comparative aetiological study. *Clinical Otolaryngology.* 2017;42(6):1358-1362.
12. Powell EL, Powell J, Samuel JR, Wilson JA. A review of the pathogenesis of adult peritonsillar abscess: time for a re-evaluation. *Journal of Antimicrobial Chemotherapy.* 2013;68(9):1941-1950.
13. Moher D, Liberati A, Tetzlaff J, Altman DG. Preferred reporting items for systematic reviews and meta-analyses: the PRISMA statement. *Annals of internal medicine.* 2009;151(4):264.
14. (SIGN) SIGN. SIGN 50: a guideline developer's handbook. *Edinburgh: SIGN; 2015.* 2015;(SIGN publication no. 50)([November 2015]). Available from URL: http://www.sign.ac.uk.
15. Gunnarsson RK, Lanke J. The predictive value of microbiologic diagnostic tests if asymptomatic carriers are present. *Statistics in Medicine.* 2002;21(12):1773-1785.
16. Bank S, Nielsen HM, Hoyer Mathiasen B, Christiansen Leth D, Hagelskjær Kristensen L, Prag J. Fusobacterium necrophorum- detection and identification on a selective agar. *APMIS.* 2010;118(12):994-999.

For peer review only - http://bmjopen.bmj.com/site/about/guidelines.xhtml

17. Tejesvi MV, Uhari M, Tapiainen T, et al. Tonsillar microbiota in children with PFAPA (periodic fever, aphthous stomatitis, pharyngitis, and adenitis) syndrome. *European Journal of Clinical Microbiology and Infectious Diseases.* 2016;35(6):963-970.

18. Bailhache M, Mariani-Kurkdjian P, Lehours P, et al. Fusobacterium invasive infections in children: A retrospective study in two French tertiary care centres. *European Journal of Clinical Microbiology and Infectious Diseases.* 2013;32(8):1041-1047.

19. Goldenberg NA, Knapp-Clevenger R, Hays T, Manco-Johnson MJ. Lemierre's and Lemierre's-like syndromes in children: survival and thromboembolic outcomes. *Pediatrics.* 2005;116(4):e543-548.

20. Papaparaskevas J, Katsandri A, Pantazatou A, et al. Epidemiological characteristics of infections caused by Bacteroides, Prevotella and Fusobacterium species: A prospective observational study. *Anaerobe.* 2011;17(3):113-117.

21. Ramirez S, Hild TG, Rudolph CN, et al. Increased diagnosis of Lemierre syndrome and other Fusobacterium necrophorum infections at a Children's Hospital. *Pediatrics.* 2003;112(5):e380.

22. Rusan M, Klug TE, Ovesen T. An overview of the microbiology of acute ear, nose and throat infections requiring hospitalisation. *European journal of clinical microbiology & infectious diseases : official publication of the European Society of Clinical Microbiology.* 2009;28(3):243-251.

23. Yusuf E, Halewyck S, Wybo I, Pierard D, Gordts F. Fusobacterium necrophorum and other Fusobacterium spp. isolated from head and neck infections: A 10-year epidemiology study in an academic hospital. *Anaerobe.* 2015;34:120-124.

24. Albertz N, Nazar G. Peritonsillar abscess: treatment with immediate tonsillectomy - 10 years of experience. *Acta Otolaryngol.* 2012;132(10):1102-1107.

For peer review only

For peer review only - http://bmjopen.bmj.com/site/about/guidelines.xhtml

25. Bank S, Christensen K, Kristensen LH, Prag J. A cost-effectiveness analysis of identifying Fusobacterium necrophorum in throat swabs followed by antibiotic treatment to reduce the incidence of Lemierre's syndrome and peritonsillar abscesses. *European journal of clinical microbiology & infectious diseases : official publication of the European Society of Clinical Microbiology.* 2013;32(1):71-78.
26. Batty A, Wren MW, Gal M. Fusobacterium necrophorum as the cause of recurrent sore throat: comparison of isolates from persistent sore throat syndrome and Lemierre's disease. *J Infect.* 2005;51(4):299-306.
27. Batty A, Wren MWD. Prevalence of Fusobacterium necrophorum and other upper respiratory tract pathogens isolated from throat swabs. *British Journal of Biomedical Science.* 2005;62(2):66-70.
28. Bjork H, Bieber L, Hedin K, Sundqvist M. Tonsillar colonisation of Fusobacterium necrophorum in patients subjected to tonsillectomy. *BMC Infect Dis.* 2015;15:264.
29. Brook I. Aerobic and anaerobic bacteriology of peritonsillar abscess in children. *Acta paediatrica Scandinavica.* 1981;70(6):831-835.
30. Brook I. Role of anaerobic beta-lactamase-producing bacteria in upper respiratory tract infections. *Pediatric Infectious Disease Journal.* 1987;6(3):310-316.
31. Brook I, Foote Jr PA, Slots J. Immune response to anaerobic bacteria in patients with peritonsillar cellulitis and abscess. *Acta Oto-Laryngologica.* 1996;116(6):888-891.
32. Brook I, Shah K, Jackson W. Microbiology of healthy and diseased adenoids. *Laryngoscope.* 2000;110(6):994-999.
33. Dapefrid A, Lundström B, Tano K. Prevalence of Fusobacterium necrophorum in tonsils from patients with chronic tonsillitis. *Acta Oto-Laryngologica.* 2017;137(3):297-301.

For peer review only

For peer review only - http://bmjopen.bmj.com/site/about/guidelines.xhtml

34. Develioglu ON, Ipek HD, Bahar H, Can G, Kulekci M, Aygun G. Bacteriological evaluation of tonsillar microbial flora according to age and tonsillar size in recurrent tonsillitis. *European Archives of Oto-Rhino-Laryngology.* 2014;271(6):1661-1665.

35. Ehlers Klug T, Rusan M, Fuursted K, Ovesen T. Fusobacterium necrophorum: most prevalent pathogen in peritonsillar abscess in Denmark. *Clinical infectious diseases : an official publication of the Infectious Diseases Society of America.* 2009;49(10):1467-1472.

36. Hagelskjaer Kristensen L, Prag J. Localised Fusobacterium necrophorum infections: a prospective laboratory-based Danish study. *European journal of clinical microbiology & infectious diseases : official publication of the European Society of Clinical Microbiology.* 2008;27(8):733-739.

37. Hagelskjær Kristensen L, Prag J. Lemierre's syndrome and other disseminated Fusobacterium necrophorum infections in Denmark: A prospective epidemiological and clinical survey. *European Journal of Clinical Microbiology and Infectious Diseases.* 2008;27(9):779-789.

38. Hayakawa K, Nagashima M, Ohta K, Ohmagari N, Tayama N. Fusobacterium necrophorum subsp. funduliforme in tonsils from various patient populations in Japan. *Japanese journal of infectious diseases.* 2018.

39. Holm K, Svensson PJ, Rasmussen M. Invasive Fusobacterium necrophorum infections and Lemièrre's syndrome: the role of thrombophilia and EBV. *European Journal of Clinical Microbiology and Infectious Diseases.* 2015;34(11):2199-2207.

40. Horn J, Bender BS, Bartlett JG. Role of anaerobic bacteria in perimandibular space infections. *The Annals of otology, rhinology & laryngology Supplement.* 1991;154:34-39.

For peer review only

For peer review only - http://bmjopen.bmj.com/site/about/guidelines.xhtml

41. Huggan PJ, Murdoch DR. Fusobacterial infections: clinical spectrum and incidence of invasive disease. *J Infect.* 2008;57(4):283-289.

42. Jensen A, Fago-Olsen H, Sorensen CH, Kilian M. Molecular mapping to species level of the tonsillar crypt microbiota associated with health and recurrent tonsillitis. *PLoS One.* 2013;8(2):e56418.

43. Jokipii AMM, Jokipii L, Sipila P, Jokinen K. Semiquantitative culture results and pathogenic significance of obligate anaerobes in peritonsillar abscesses. *Journal of Clinical Microbiology.* 1988;26(5):957-961.

44. Klug TE. Incidence and microbiology of peritonsillar abscess: the influence of season, age, and gender. *European journal of clinical microbiology & infectious diseases : official publication of the European Society of Clinical Microbiology.* 2014;33(7):1163-1167.

45. Klug TE, Fischer AS, Antonsen C, Rusan M, Eskildsen H, Ovesen T. Parapharyngeal abscess is frequently associated with concomitant peritonsillar abscess. *Eur Arch Otorhinolaryngol.* 2014;271(6):1701-1707.

46. Klug TE, Henriksen JJ, Fuursted K, Ovesen T. Significant pathogens in peritonsillar abscesses. *European journal of clinical microbiology & infectious diseases : official publication of the European Society of Clinical Microbiology.* 2011;30(5):619-627.

47. Lepelletier D, Pinaud V, Le Conte P, et al. Is there an association between prior anti-inflammatory drug exposure and occurrence of peritonsillar abscess (PTA)? A national multicenter prospective observational case–control study. *European Journal of Clinical Microbiology and Infectious Diseases.* 2017;36(1):57-63.

48. Mitchelmore IJ, Prior AJ, Montgomery PQ, Tabaqchali S. Microbiological features and pathogenesis of peritonsillar abscesses. *European journal of clinical microbiology &*

For peer review only

For peer review only - http://bmjopen.bmj.com/site/about/guidelines.xhtml

infectious diseases : official publication of the European Society of Clinical Microbiology. 1995;14(10):870-877.

49. Price SL, Hardy S, Gale P, Basten GP. Prevalence of Fusobacterium necrophorum in persistent sore throat samples. *Br J Biomed Sci.* 2011;68(4):209-210.
50. Tachibana T, Orita Y, Takao S, et al. The role of bacteriological studies in the management of peritonsillar abscess. *Auris Nasus Larynx.* 2016;43(6):648-653.
51. Wiksten JE, Laakso S, Maki M, Makitie AA, Pitkaranta A, Blomgren K. Microarray identification of bacterial species in peritonsillar abscesses. *European journal of clinical microbiology & infectious diseases : official publication of the European Society of Clinical Microbiology.* 2015;34(5):905-911.
52. Amess JA, O'Neill W, Ni Giollariabhaigh C, Dytrych JK. A six-month audit of the isolation of Fusobacterium necrophorum from patients with sore throat in a district general hospital. *British Journal of Biomedical Science.* 2007;64(2):63-65.
53. Suzuki K, Kurono Y, Ikeda K, et al. Nationwide surveillance of 6 otorhinolaryngological infectious diseases and antimicrobial susceptibility pattern in the isolated pathogens in Japan. *Journal of Infection and Chemotherapy.* 2015;21(7):483-491.
54. Van TT, Cox LM, Cox ME, Dien Bard J. Prevalence of Fusobacterium necrophorum in Children Presenting with Pharyngitis. *J Clin Microbiol.* 2017;55(4):1147-1153.
55. Windfuhr JP, Toepfner N, Steffen G, Waldfahrer F, Berner R. Clinical practice guideline: tonsillitis I. Diagnostics and nonsurgical management. *European Archives of Oto-Rhino-Laryngology.* 2016;273(4):973-987.
56. Aliyu SH, Marriott RK, Curran MD, et al. Real-time PCR investigation into the importance of Fusobacterium necrophorum as a cause of acute pharyngitis in general practice. *J Med Microbiol.* 2004;53(Pt 10):1029-1035.

For peer review only

For peer review only - http://bmjopen.bmj.com/site/about/guidelines.xhtml

57. Atkinson TP, Centor RM, Xiao L, et al. Analysis of the tonsillar microbiome in young adults with sore throat reveals a high relative abundance of Fusobacterium necrophorum with low diversity. *PLoS One.* 2018;13(1):e0189423.

58. Eaton C, Swindells J. The significance and epidemiology of Fusobacterium necrophorum in sore throats. *J Infect.* 2014;69(2):194-196.

59. Ludlam H, Howard J, Kingston B, et al. Epidemiology of pharyngeal carriage of Fusobacterium necrophorum. *J Med Microbiol.* 2009;58(Pt 9):1264-1265.

60. Pallon J, Sundqvist M, Hedin K. A 2-year follow-up study of patients with pharyngotonsillitis. *BMC Infect Dis.* 2018;18(1):3.

61. Centor RM, Atkinson TP, Ratliff AE, et al. The clinical presentation of Fusobacterium-positive and streptococcal-positive pharyngitis in a university health clinic: a cross-sectional study. *Ann Intern Med.* 2015;162(4):241-247.

62. Hedin K, Bieber L, Lindh M, Sundqvist M. The aetiology of pharyngotonsillitis in adolescents and adults - Fusobacterium necrophorum is commonly found. *Clinical microbiology and infection : the official publication of the European Society of Clinical Microbiology and Infectious Diseases.* 2015;21(3):263.e261-267.

63. Kjaerulff AM, Thomsen MK, Ovesen T, Klug TE. Clinical and biochemical characteristics of patients with Fusobacterium necrophorum-positive acute tonsillitis. *Eur Arch Otorhinolaryngol.* 2015;272(6):1457-1463.

64. Hayakawa K, Nagashima M, Kanehisa E, et al. Real-time PCR investigation of the prevalence of Fusobacterium necrophorum in patients with pharyngitis in Japan. *J Infect Chemother.* 2018;24(12):969-974.

65. Jensen A, Hagelskjær Kristensen L, Prag J. Detection of fusobacterium necrophorum subsp. funduliforme in tonsillitis in young adults by real-time PCR. *Clinical Microbiology and Infection.* 2007;13(7):695-701.

For peer review only - http://bmjopen.bmj.com/site/about/guidelines.xhtml

66. Jensen A, Hansen TM, Bank S, Kristensen LH, Prag J. Fusobacterium necrophorum tonsillitis: an important cause of tonsillitis in adolescents and young adults. *Clinical microbiology and infection : the official publication of the European Society of Clinical Microbiology and Infectious Diseases.* 2015;21(3):266.e261-263.

67. Gunnarsson RK, Manchal N. Group C beta hemolytic Streptococci as a potential pathogen in patients presenting with an uncomplicated acute sore throat - a systematic literature review and meta-analysis. *Scand J Prim Health Care.* 2020:1-12.

For peer review only

For peer review only - http://bmjopen.bmj.com/site/about/guidelines.xhtml

Table 1 – Case-control studies examining Fusobacterium necrophorum and Group A Streptococcus in patients with an acute uncomplicated sore throat in primary care

Study [ref]	Design	Method	Age, years (range)		Number of cases and controls			% FN detected (number)			% GAS detected (number)		
			Cases	Controls	Cases		Controls	Cases		Controls	Cases		Controls
					Centor 0-4	Centor 3-4		Centor 0-4	Centor 3-4		Centor 0-4	Centor 3-4	
Hedin 2015 [62]	Pro	Culture	33 (15–48)	31 (16-46)	220	85	128	15% (33)	19% (16)	3.1% (4)	30% (66)	49% (42)	2.3% (3)
Centor 2015 [61]	Pro	PCR	22 (15-30)	24 (15-30)	312	64	180	21% (64)	23% (15)	9.4% (17)	10% (32)	16% (10)	1.1% (2)
Kjærulff 2015 [63]	Pro	Culture	28 (15-40)	29 (15-40)	100	29	100	16% (16)	21% (6)	9.0 % (9)	26% (26)	48% (14)	3.0% (3)
Hayakawa 2018 [64]	Pro	PCR + culture	29 (25-37)	33 (26-36)	44	19	31	14% (6)	21% (4)	6.5% (2)	11% (5)	16% (3)	0.0% (0)
SUBTOTAL (HIGH QUALITY ARTICLES)					***676***	***197***	***439***	***18% (119)***	***21% (41)***	***7.2% (32)***	***19% (129)***	***35% (69)***	***1.8(8)***
Jensen 2007 [65]	Pro	PCR + culture	25 (18-32)	22 (18-32)	105	-	92	51% (54)	-	21% (19)	5.7%* (6*)	-	-
Jensen 2015 [66]	Retro	Culture	19 (10-40)	22 (10-40)	179	-	176	24% (43)	-	5.7% (10)	3.9%* (7*)	-	0% (0)
SUBTOTAL (INFERIOR QUALITY ARTICLES)					*284*	-	*268*	*34% (97)*	-	*11% (29)*	*4.6%* (13*)*	-	-
TOTAL (ALL SIX ARTICLES)					960	178	707	23% (216)	21% (37)	8.6% (61)	15%* (142*)	37% (66)	1.1% (8)

*GAS-tonsillitis was excluded (by GPs using rapid antigen tests) in the two articles by Jensen et al.; thus, those results for GAS were irrelevant for the purpose of this meta-analysis.

For peer review only

For peer review only - http://bmjopen.bmj.com/site/about/guidelines.xhtml

Figure 1 – PRISMA Flow Diagram

Figure 2 – Quality assessment of included studies

Figure 3 - Probability of a true link between sore throat with Centor score 0-4 and positive FN test

*Positive etiologic predictive value is the probability of a true link between sore throat and FN based on studies with data from both patients and healthy controls.

Figure 4 - Probability of a true link between the sore throat with Centor score 3-4 and positive FN test

*Positive etiologic predictive value is the probability of a true link between sore throat and FN based on studies with data from both patients and healthy controls.

Figure 5 - Probability of a true link between sore throat with Centor score 0-4 and positive GAS test

*Positive etiologic predictive value is the probability of a true link between sore throat and GAS based on studies with data from both patients and healthy controls.
Since the study by Hayakawa 2018 found no positive tests for GAS among healthy controls, P-EPV could not be calculated for that study separately. However, the results from the four studies selected for meta-analysis are included in the summary.

Figure 6 - Probability of a true link between sore throat with Centor score 3-4 and positive GAS test

*Positive etiologic predictive value is the probability of a true link between sore throat and GAS based on studies with data from both patients and healthy controls.
Since the study by Hayakawa 2018 found no positive tests for GAS among healthy controls, P-EPV could not be calculated for that study separately. However, the results from the four studies selected for meta-analysis are included in the summary.

For peer review only - http://bmjopen.bmj.com/site/about/guidelines.xhtml

For peer review only

Acute Sore Throat and Fusobacterium Necrophorum in PHC – A Systematic Review and Meta-analysis
Version: (7/26/2020 8:14:00 AM)

Appendix 1: Search strings for PubMed and PROSPERO

PubMed:

(("pharyngitis"[MH] OR "pharyngitis"[TIAB] OR "Lemierre Syndrome"[MH] OR "Lemierre Syndrome"[TIAB] OR "Necrobacillosis"[TIAB] OR "pharyngotonsillitis "[TIAB] OR "tonsillitits"[TIAB] OR "throat"[TIAB] OR "epidemiology"[TIAB])

AND ("fusobacterium necrophorum"[MH] OR "fusobacterium"[TIAB]))

NOT "Case Reports"[pt]

Scopus:

(TITLE-ABS-KEY ((pharyngitis OR "Lemierre Syndrome" OR necrobacillosis OR pharyngotonsillitis OR tonsillitis OR "Peritonsillar Abscess" OR throat OR epidemiology))

AND TITLE-ABS-KEY (fusobacterium))

AND DOCTYPE (ar)

AND (LIMIT-TO (SUBJAREA , "MEDI ") OR LIMIT-TO (SUBJAREA , " IMMU ")

OR LIMIT-TO (SUBJAREA , " BIO "))

AND (LIMIT-TO (LANGUAGE , "English "))

AND (EXCLUDE (EXACTKEYWORD , "Case Report"))

For peer review only

For peer review only - http://bmjopen.bmj.com/site/about/guidelines.xhtml

Acute Sore Throat and Fusobacterium Necrophorum in PHC – A Systematic Review and Meta-analysis
Version: (15/7/20 12:12:00 pm)

PRISMA 2009 Flow Diagram

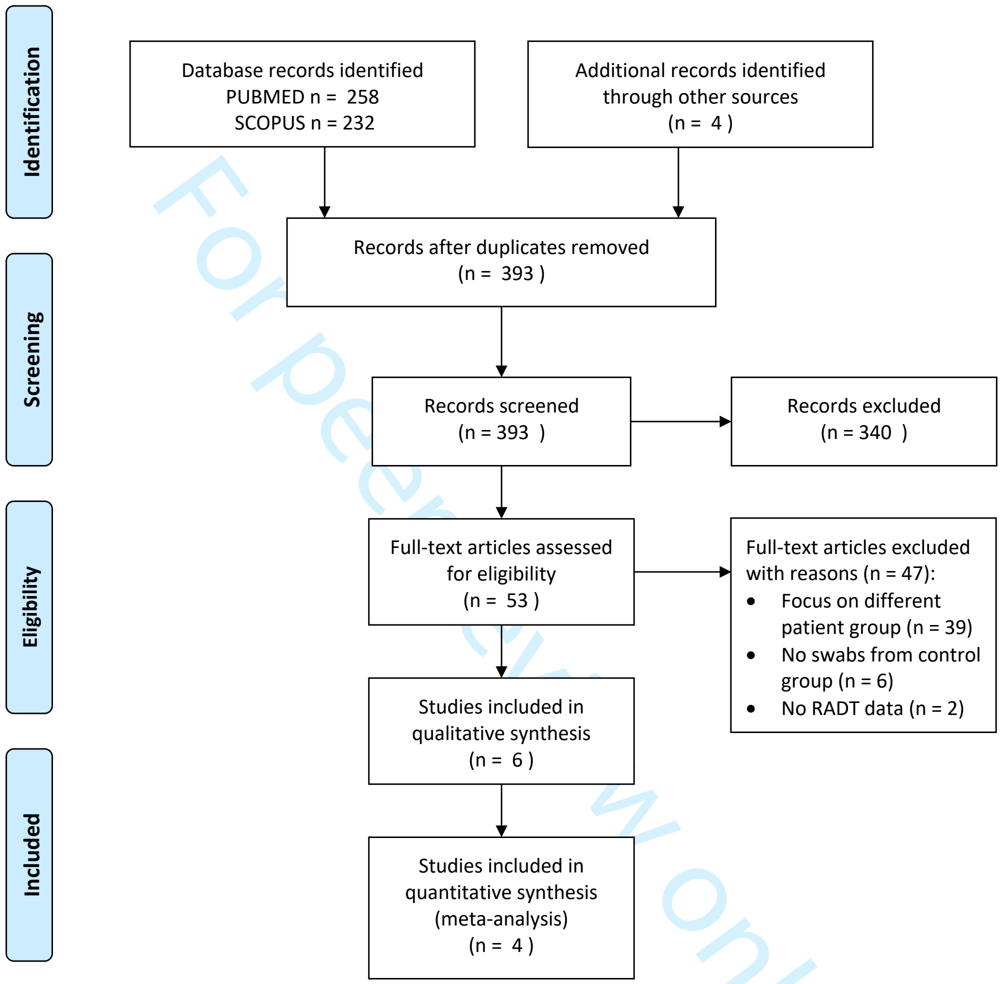

For peer review only

From: Moher D, Liberati A, Tetzlaff J, Altman DG, The PRISMA Group (2009). *P*referred *R*eporting *I*tems for *S*ystematic Reviews and *M*eta-*A*nalyses: The PRISMA Statement. PLoS Med 6(7): e1000097. doi:10.1371/journal.pmed1000097

For more information, visit www.prisma-statement.org.

Figure 1- PRISMA Flow Diagram

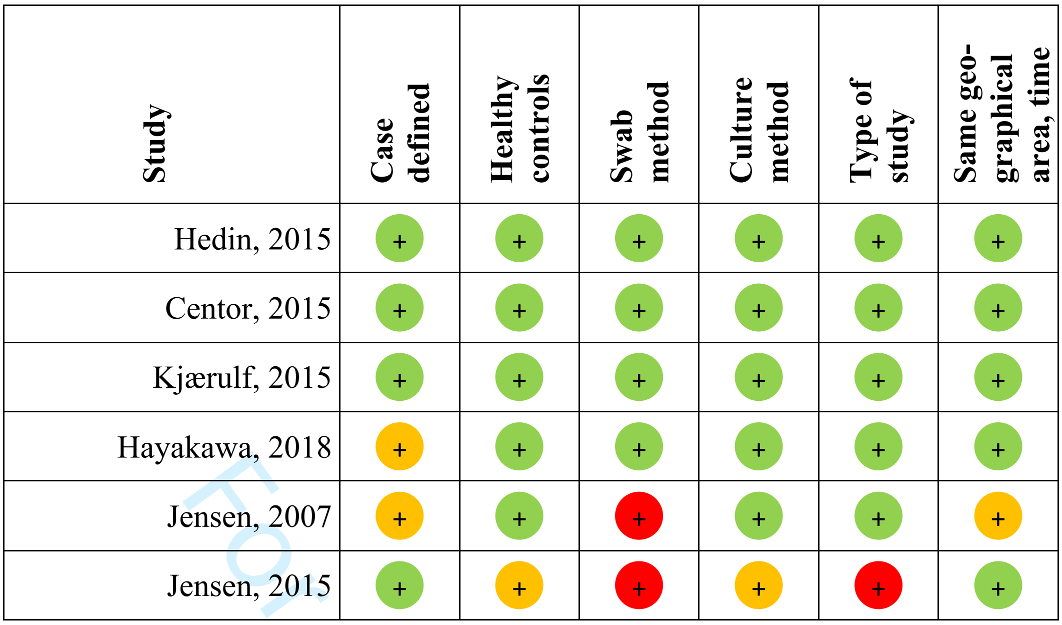

Figure 2 - Quality assessment of included studies

+ Low risk of bias + Intermediate risk of bias + High risk of bias

For peer review only

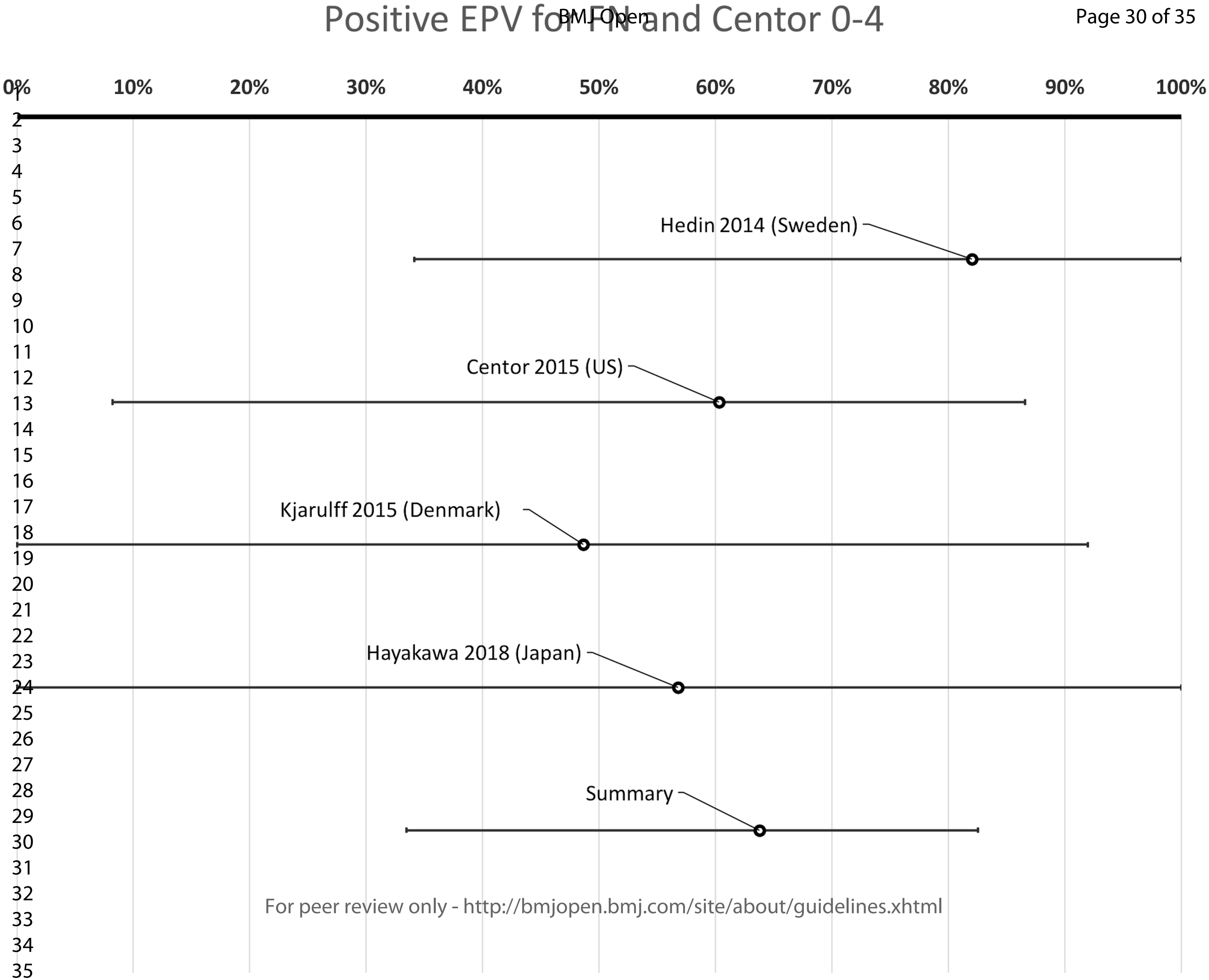

For peer review only - http://bmjopen.bmj.com/site/about/guidelines.xhtml

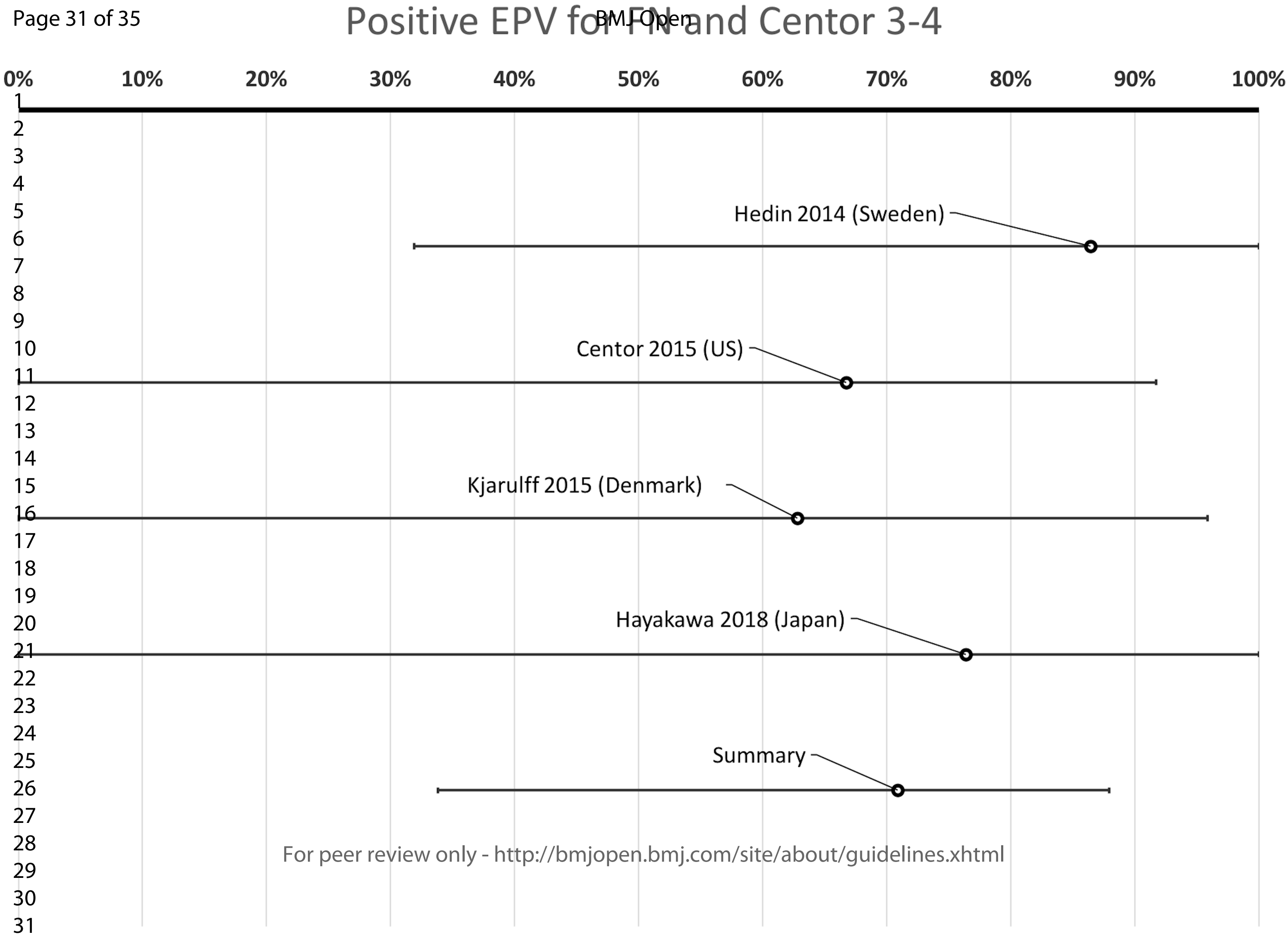
Positive EPV for FN and Centor 3-4
0%
10%
20%
30%
40%
50%
60%
70%
80%
90%
100%
Hedin 2014 (Sweden)
Centor 2015 (US)
Kjarulff 2015 (Denmark)
Hayakawa 2018 (Japan)
Summary

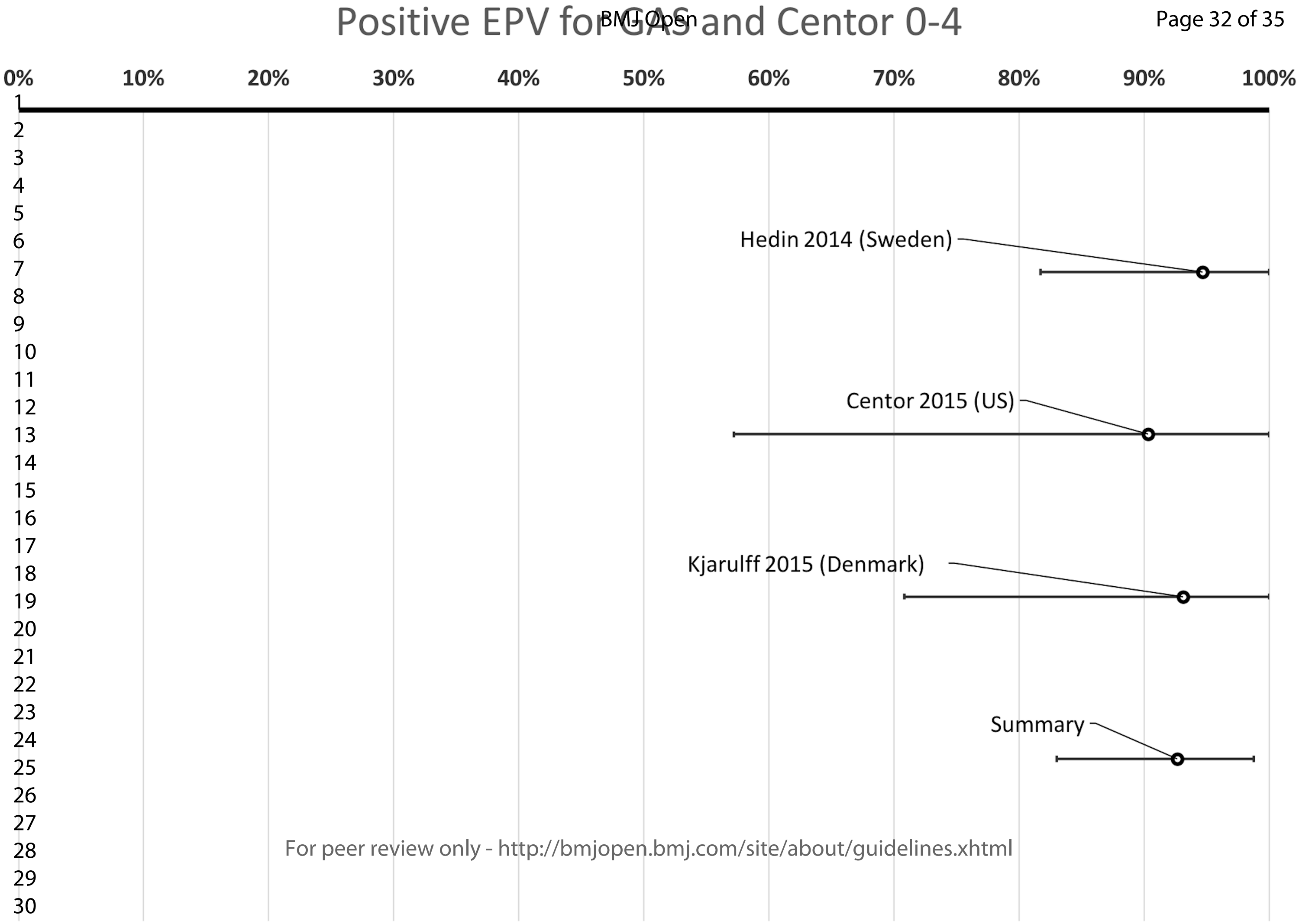
Positive EPV for GAS and Centor 0-4
0%
10%
20%
30%
40%
50%
60%
70%
80%
90%
100%
Hedin 2014 (Sweden)
Centor 2015 (US)
Kjarulff 2015 (Denmark)
Summary

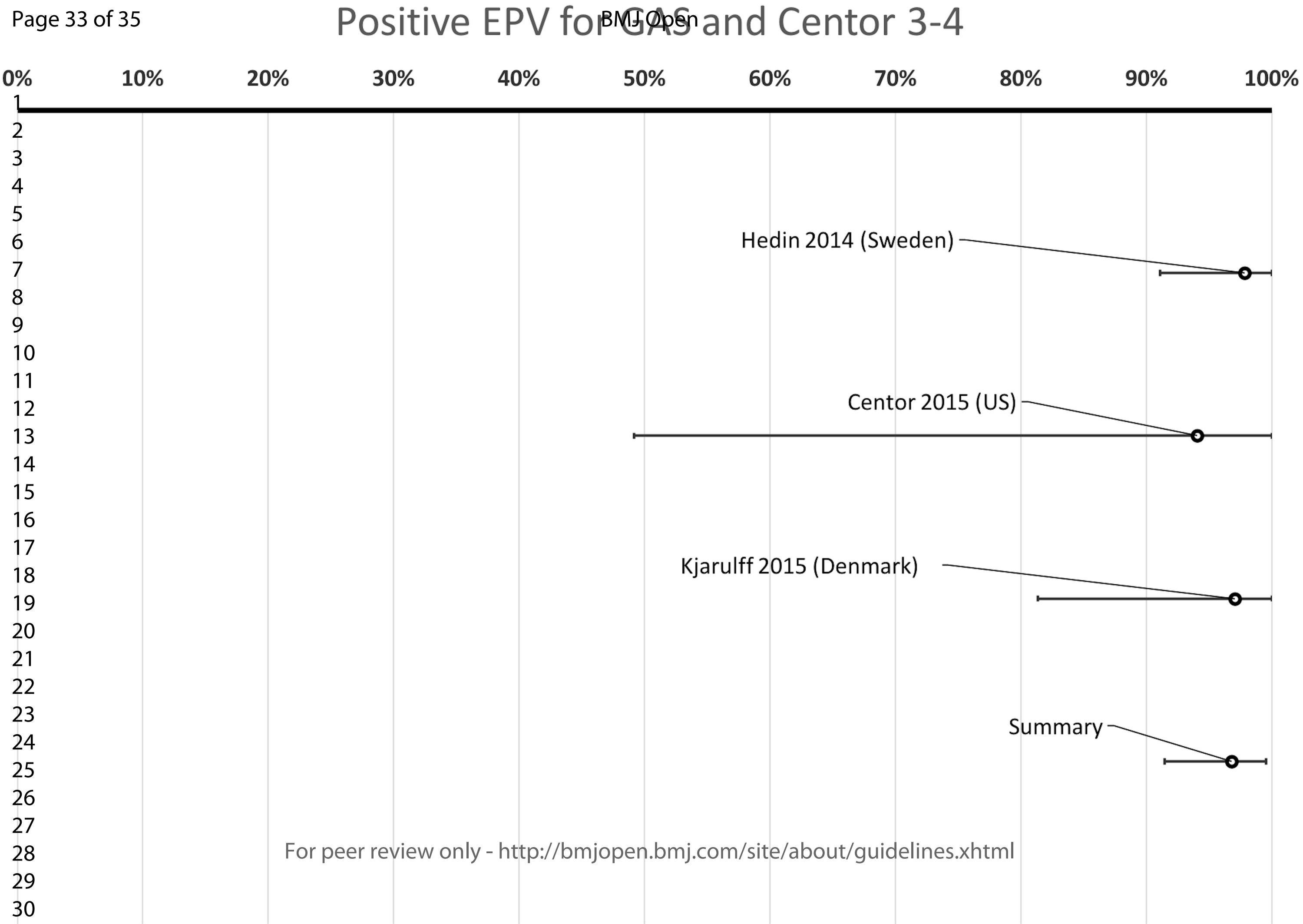

For peer review only - http://bmjopen.bmj.com/site/about/guidelines.xhtml

PRISMA 2009 Checklist

Section/topic	#	Checklist item	Reported on page #
**TITLE**			
Title	1	Identify the report as a systematic review, meta-analysis, or both.	1
**ABSTRACT**			
Structured summary	2	Provide a structured summary including, as applicable: background; objectives; data sources; study eligibility criteria, participants, and interventions; study appraisal and synthesis methods; results; limitations; conclusions and implications of key findings; systematic review registration number.	2
**INTRODUCTION**			
Rationale	3	Describe the rationale for the review in the context of what is already known.	4
Objectives	4	Provide an explicit statement of questions being addressed with reference to participants, interventions, comparisons, outcomes, and study design (PICOS).	4
**METHODS**			
Protocol and registration	5	Indicate if a review protocol exists, if and where it can be accessed (e.g., Web address), and, if available, provide registration information including registration number.	4
Eligibility criteria	6	Specify study characteristics (e.g., PICOS, length of follow-up) and report characteristics (e.g., years considered, language, publication status) used as criteria for eligibility, giving rationale.	5
Information sources	7	Describe all information sources (e.g., databases with dates of coverage, contact with study authors to identify additional studies) in the search and date last searched.	5
Search	8	Present full electronic search strategy for at least one database, including any limits used, such that it could be repeated.	31 (Appendix 1)
Study selection	9	State the process for selecting studies (i.e., screening, eligibility, included in systematic review, and, if applicable, included in the meta-analysis).	5
Data collection process	10	Describe method of data extraction from reports (e.g., piloted forms, independently, in duplicate) and any processes for obtaining and confirming data from investigators.	6
Data items	11	List and define all variables for which data were sought (e.g., PICOS, funding sources) and any assumptions and simplifications made.	6
Risk of bias in individual studies	12	Describe methods used for assessing risk of bias of individual studies (including specification of whether this was done at the study or outcome level), and how this information is to be used in any data synthesis.	6 and 26 (figure 2)
Summary measures	13	State the principal summary measures (e.g., risk ratio, difference in means).	6-7

For peer review only

PRISMA 2009 Checklist

Synthesis of results	14	Describe the methods of handling data and combining results of studies, if done, including measures of consistency (e.g., I^2) for each meta-analysis.	6-7

Section/topic	#	Checklist item	Reported on page #
Risk of bias across studies	15	Specify any assessment of risk of bias that may affect the cumulative evidence (e.g., publication bias, selective reporting within studies).	24 and 26
Additional analyses	16	Describe methods of additional analyses (e.g., sensitivity or subgroup analyses, meta-regression), if done, indicating which were pre-specified.	7 and 27-30
**RESULTS**			
Study selection	17	Give numbers of studies screened, assessed for eligibility, and included in the review, with reasons for exclusions at each stage, ideally with a flow diagram.	25
Study characteristics	18	For each study, present characteristics for which data were extracted (e.g., study size, PICOS, follow-up period) and provide the citations.	24
Risk of bias within studies	19	Present data on risk of bias of each study and, if available, any outcome level assessment (see item 12).	7-10 and 26
Results of individual studies	20	For all outcomes considered (benefits or harms), present, for each study: (a) simple summary data for each intervention group (b) effect estimates and confidence intervals, ideally with a forest plot.	24
Synthesis of results	21	Present results of each meta-analysis done, including confidence intervals and measures of consistency.	10 and 27-30
Risk of bias across studies	22	Present results of any assessment of risk of bias across studies (see Item 15).	9-10 and 26
Additional analysis	23	Give results of additional analyses, if done (e.g., sensitivity or subgroup analyses, meta-regression [see Item 16]).	11 and 27-30
**DISCUSSION**			
Summary of evidence	24	Summarize the main findings including the strength of evidence for each main outcome; consider their relevance to key groups (e.g., healthcare providers, users, and policy makers).	10
Limitations	25	Discuss limitations at study and outcome level (e.g., risk of bias), and at review-level (e.g., incomplete retrieval of identified research, reporting bias).	11-12
Conclusions	26	Provide a general interpretation of the results in the context of other evidence, and implications for future research.	12-13
**FUNDING**			

For peer review only

For peer review only - http://bmjopen.bmj.com/site/about/guidelines.xhtml

PRISMA 2009 Checklist

Funding	27	Describe sources of funding for the systematic review and other support (e.g., supply of data); role of funders for the systematic review.	14

From: Moher D, Liberati A, Tetzlaff J, Altman DG, The PRISMA Group (2009). Preferred Reporting Items for Systematic Reviews and Meta-Analyses: The PRISMA Statement. PLoS Med 6(7): e1000097. doi:10.1371/journal.pmed1000097

For more information, visit: www.prisma-statement.org.

For peer review only

For peer review only - http://bmjopen.bmj.com/site/about/guidelines.xhtml

BMJ Open

Acute Sore Throat and Fusobacterium Necrophorum in Primary Health Care – A Systematic Review and Meta-analysis

Journal:	*BMJ Open*
Manuscript ID	bmjopen-2020-042816.R1
Article Type:	Original research
Date Submitted by the Author:	18-Jan-2021

Complete List of Authors:	Malmberg, Stefan; University of Gothenburg Sahlgrenska Academy, School of Public health and Community Medicine<br>Petrén, Susanna; University of Gothenburg Sahlgrenska Academy, School of Public health and Community Medicine<br>Gunnarsson, Ronny; University of Gothenburg Sahlgrenska Academy, School of Public Health and Community Medicine; Vastra Gotalandsregion, Regionhalsan, FoUU<br>Hedin, Katarina; Linköpings universitet Institutionen för medicin och hälsa; Region Jönköpings län, Futurum academy for health and care<br>Sundvall, Pär-Daniel ; Vastra Gotalandsregion, Research and Development Primary Health Care; University of Gothenburg Sahlgrenska Academy, School of Public health and Community Medicine

**Primary Subject Heading**:	General practice / Family practice
Secondary Subject Heading:	Diagnostics, Infectious diseases, Epidemiology
Keywords:	Epidemiology < INFECTIOUS DISEASES, PRIMARY CARE, INFECTIOUS DISEASES, Diagnostic microbiology < INFECTIOUS DISEASES

SCHOLARONE™
Manuscripts

I, the Submitting Author has the right to grant and does grant on behalf of all authors of the Work (as defined in the below author licence), an exclusive licence and/or a non-exclusive licence for contributions from authors who are: i) UK Crown employees; ii) where BMJ has agreed a CC-BY licence shall apply, and/or iii) in accordance with the terms applicable for US Federal Government officers or employees acting as part of their official duties; on a worldwide, perpetual, irrevocable, royalty-free basis to BMJ Publishing Group Ltd ("BMJ") its licensees and where the relevant Journal is co-owned by BMJ to the co-owners of the Journal, to publish the Work in this journal and any other BMJ products and to exploit all rights, as set out in our licence.

The Submitting Author accepts and understands that any supply made under these terms is made by BMJ to the Submitting Author unless you are acting as an employee on behalf of your employer or a postgraduate student of an affiliated institution which is paying any applicable article publishing charge ("APC") for Open Access articles. Where the Submitting Author wishes to make the Work available on an Open Access basis (and intends to pay the relevant APC), the terms of reuse of such Open Access shall be governed by a Creative Commons licence – details of these licences and which Creative Commons licence will apply to this Work are set out in our licence referred to above.

Other than as permitted in any relevant BMJ Author's Self Archiving Policies, I confirm this Work has not been accepted for publication elsewhere, is not being considered for publication elsewhere and does not duplicate material already published. I confirm all authors consent to publication of this Work and authorise the granting of this licence.

For peer review only

Acute Sore Throat and Fusobacterium Necrophorum in PHC – A Systematic Review and Meta-analysis
Version: (11 January 2021)

**Title**	**Acute Sore Throat and Fusobacterium Necrophorum in Primary Health Care – A Systematic Review and Meta-analysis**
**Authors' name**	Stefan Malmberg[1,2], Susanna Petrén[3], Ronny Gunnarsson[1,4,5], Katarina Hedin[6,7], Pär-Daniel Sundvall[1,4,5]
**Address for each author**	[1] Region Västra Götaland, Research and Development Primary Health Care, Research and Development Centre Södra Älvsborg, Sven Eriksonsplatsen 4, SE-503 38 Borås, Sweden<br>[2] HälsoBrunnen VC, Praktikertjänst AB, Vistvägen 2, 52337 Ulricehamn, Sweden.<br>[3] Närhälsan Högsbo vårdcentral, Markmyntsgatan 14A, 414 80 Gothenburg, Sweden<br>[4] General Practice / Family Medicine, Department of Public Health and Community Medicine, Institute of Medicine, Sahlgrenska Academy, University of Gothenburg, Box 454, SE-405 30 Gothenburg Sweden<br>[5] Centre for Antibiotic Resistance Research (CARe) at University of Gothenburg, Gothenburg, Sweden<br>[6] Futurum, Region Jönköping County, Jönköping and Department of Health, Medicine and Caring Sciences, Linköping University, Linköping, Sweden<br>[7] Department of Clinical Sciences in Malmö, Family Medicine, Lund University, Malmö, Sweden
**Authors' names and positions**	Stefan Malmberg MD PhD student, Susanna Petrén MD, Ronny Gunnarsson MD PhD Professor in General Practice, Katarina Hedin MD PhD and Associate Professor in General Practice, Pär-Daniel Sundvall MD PhD
**Corresponding author**	Stefan Malmberg,<br>stefan.malmberg@stfn.se
**Word count**	Abstract: 262<br>Manuscript excluding abstract, references and tables: 3,221

For peer review only - http://bmjopen.bmj.com/site/about/guidelines.xhtml

Acute Sore Throat and Fusobacterium Necrophorum in PHC – A Systematic Review and Meta-analysis
Version: (11 January 2021)

Abstract

Purpose: The main objective of this review was to describe and quantify the association between *Fusobacterium necrophorum* (FN) and acute sore throat in primary health care.

Methods: In this systematic review and meta-analysis, we searched Scopus and PubMed for case-control studies reporting the prevalence of FN in patients attending primary care for an uncomplicated acute sore throat as well as in healthy controls. Only studies published in English were considered. Publications were not included if they were case studies, or if they included patients prescribed antibiotics before the throat swab, patients with a concurrent malignant disease, on immunosuppression, having an HIV infection, or patients having another acute infection in addition to a sore throat. Inclusion criteria and methods were specified in advance and published in PROSPERO. The primary outcome was positive etiologic predictive value (P-EPV), quantifying the probability for an association between acute sore throat and findings of FN in the pharynx. For comparison, our secondary outcome was the corresponding P-EPV for group A streptococcus (GAS). **Results**: PubMed and Scopus yielded 258 and 232 studies, respectively. Removing duplicates and screening the abstracts resulted in 53 studies subsequently read in full text. For the four studies of medium to high quality included in the meta-analysis, the cumulative P-EPV regarding FN was 64% (95% CI 33-83%). GAS, based on data from the same publications and patients, yielded a positive EPV of 93% (95% CI 83-99%). **Conclusions**: The results indicate that FN may play a role in primary health care patients with an acute sore throat, but the association is much weaker compared to GAS.

For peer review only - http://bmjopen.bmj.com/site/about/guidelines.xhtml

Article Summary

Strengths and limitations of this study

- This is the first systematic literature review with meta-analysis utilizing positive etiologic predictive value to quantify the clinical relevance of a finding of *Fusobacterium necrophorum* in patients presenting with an uncomplicated acute sore throat in PHC.
- The positive etiologic predictive value reveals the probability for a true link between *Fusobacterium necrophorum* and the sore throat expressed as a plain percentage between 0-100%.
- A potential limitation is that there were only four available case-control studies presenting the proportion of FN.

For peer review only - http://bmjopen.bmj.com/site/about/guidelines.xhtml

Introduction

An uncomplicated acute sore throat is a common reason for attending primary health care (PHC).[1-3] Most current guidelines concerning the management of patients with a sore throat focus on group A s*treptococci* (GAS).[4-6] However, recent studies have indicated that *Fusobacterium necrophorum* (FN) might cause a sore throat, particularly among adolescents and young adults.[7-9]

FN is an anaerobic gram-negative bacterium most known for causing the severe disease Lemierre's syndrome, a potentially life-threatening condition which typically begins with a sore throat, and is also an established pathogen in peritonsillar abscess (PTA).[10-12]

The role of FN in the sore throat has been studied in three recent reviews.[7-9] None of the three reviews have taken into consideration the carriage rate of FN in healthy controls, which is of importance when estimating the clinical relevance of finding FN in patients with an uncomplicated acute sore throat.

This study aimed to estimate the probability for an association between FN and the uncomplicated acute sore throat in patients attending PHC, when taking into consideration the carriage rate of FN in healthy controls. A second aim was to compare the probability for FN with the same probability for an association between GAS and patients with an uncomplicated acute sore throat.

Patient and public involvement

No patients involved.

Methods

For peer review only - http://bmjopen.bmj.com/site/about/guidelines.xhtml

The review was conducted in accordance with the Preferred Reporting Items for Systematic Reviews and Meta-Analyses (PRISMA) statement.[13] Inclusion criteria and methods were specified in advance and documented in the review protocol. The initial protocol was registered and made available beforehand in PROSPERO (International Prospective Register of Systematic Reviews), September 5, 2018 (registration number CRD42018106800).

Search Strategy

PubMed and SCOPUS were searched (17 September 2018) for case-control studies reporting the prevalence of FN in patients with an uncomplicated acute sore throat and in healthy individuals without any signs of infection. There were no time limitations. The search terms are described in Appendix 1.

Study Selection

All case-control studies reporting the prevalence of FN in patients attending a PHC setting for an uncomplicated acute sore throat and in a healthy control group were included. Only studies published in English were considered. Publications were not included if they were case studies, or if they included patients prescribed antibiotics before the throat swab, patients with a concurrent malignant disease, on immunosuppression, having an HIV infection, or patients having another acute infection in addition to a sore throat.

SM performed the first screening by reading titles and abstract to remove duplicates from the two search strategies and, thereafter, to remove obviously irrelevant studies such as animal studies. The remaining studies were carefully screened again reading titles and abstracts independently by SM and SP to identify studies that potentially met the inclusion criteria outlined above. SM and SP started screening sitting together in the same room discussing each publication to ensure they aligned their judgement. They then continued

For peer review only - http://bmjopen.bmj.com/site/about/guidelines.xhtml

screening separately but had a joint discussion whenever they decided differently if a publication should be kept or removed

The full texts of these potentially eligible studies were retrieved and independently assessed for eligibility by SM and SP. Any disagreement between them over the eligibility of particular studies was resolved through discussion within the whole review team.

The reference lists for each article were screened for additional articles potentially matching the inclusion criteria. Such articles were added to the list of potentially eligible studies for further assessment.

For peer review only

Appraisal of methodological quality

SM and SP independently assessed the risk of bias in the included studies by using methodological quality characteristics (Table 1). Overall high quality was defined as having a low risk of bias in all criteria. Having a high risk of bias in any criteria made the study to be of overall low quality. The rest was classified as being of an overall medium quality. Disagreements over the risk of bias in particular studies were resolved by discussion within the review team.

Data extraction

A standardized, pre-piloted form was used to extract data from the included studies for assessment of methodological quality and evidence synthesis. Extracted information included: study setting, definition of cases and classification of these using the Centor criteria if available, definition of healthy controls, swab method, culture or PCR method, outcomes of throat swabs, and information for the assessment of the risk of bias. SM and SP extracted data independently. Discrepancies were identified and resolved through discussion.

For peer review only - http://bmjopen.bmj.com/site/about/guidelines.xhtml

Data analysis

A narrative synthesis was produced for each of the included studies, structured around the study methodology, target population characteristics, outcome, and the assessment of methodological quality.

Studies with a healthy control group and of a medium to high methodological quality were used for the meta-analysis, where the pooled difference in prevalence of FN between cases and healthy controls was compared using chi-square test.

The clinical relevance of any statistical differences between symptomatic patients and healthy controls was further explored by calculating the positive etiologic predictive value (P-EPV)[14]. P-EPV is a method of quantifying the probability of a true link between the symptom (a sore throat) and the finding of FN in the throat while considering the possibility of healthy carriers of FN (Appendix 2). P-EPV for FN was, when possible, compared to P-EPV for GAS using data from the same patients and publications.

Using a random effects meta-analysis would have provided odds ratios for harboring FN among cases compared to controls. This type of effect size does not give an easy-to-understand interpretation to a clinician. The statistical technique we used for meta-analysis, P-EPV, has been used previously[15] and provides a probability for a true connection between FN and the symptom of a sore throat in the studied group. This outcome is a plain percent between 0-100%. If a bacterium is found equally often in patients and controls the point estimate of P-EPV will be 0% with a 95% confidence interval from 0.0% and the upper limit will be determined by the sample size. The point estimate of P-EPV will approach 100% when the difference in prevalence of a bacterium between patients and controls increases. This was in our opinion a more clinically useful outcome that can be easily understood by clinicians.

For peer review only - http://bmjopen.bmj.com/site/about/guidelines.xhtml

P-EPV does not directly take into account between-study variation so it is not a random effect model. We compensate for this by presenting our outcome for each individual study as well as a sensitivity analysis where we compare the consequences of combining them differently. Furthermore, the between-study variation statistics (I^2) calculated in random effect models is very unreliable when the number of included publications is small [16] and we knew already from the start that the number of available publications would be small.

P-EPV has the ability to adjust the proportion of individuals harboring FN between healthy controls and symptomatic carriers ill from something else like a virus. However, in this review we chose to not adjust this, so we set theta to 1.0 meaning that symptomatic carriers harbor FN equally often as healthy controls. Finally, P-EPV allows us to consider the sensitivity of the test to detect FN, something that conventional random effects meta-analysis doesn't.

Results

The PubMed search yielded 258 publications, and the Scopus database query yielded 232 (Figure 1). Reviewing reference lists did not reveal any more relevant publications not found in the initial searches. Removing duplicates and screening the abstracts resulted in 53 studies subsequently read in full text.

Exclusion of publications

Thirty-seven of these 53 articles were not included because they had a different focus, i.e. lab methods [17], or focused on a different category of patients than was the scope of this review: chronically ill patients [18], hospitalized patients [19-24], or patients with a subset of infections such as peritonsillar abscess, Lemierre's syndrome, chronic/recurrent tonsillitis, and intra-abdominal infections [11 25-52]. Four were excluded because they lacked a control group [53-56].

For peer review only - http://bmjopen.bmj.com/site/about/guidelines.xhtml
For peer review only

Discussions in the review team prompted the exclusion of another five articles with methodological limitations in relation to the scope of this review.[57-61]

The article published in 2004 by Aliyu et al.[57] concerned a study where routine throat swabs were analyzed for FN-specific DNA and compared with swabs obtained from healthy adults. The cases were randomly selected in the lab, but it was unclear to what extent they had a sore throat and how these symptoms were registered. The cases included children as young as five months. Hence, it was unclear what kind of patients the routine throat swabs sent to the laboratory represented. Inclusion in the control group required the absence of antibiotic therapy in the preceding two weeks, but not for cases. The swabs from cases were also cultured for GAS, but there was no information about the prevalence of GAS in the control group. The mean age and range differed substantially between cases and controls.

The article published in 2018 by Atkinson et al.[58] presented the results of applying a new laboratory method on swabs from a previously published study [62] and comparing the results to the method that was used initially. The cases included university students complaining of a sore throat, while the controls were asymptomatic students. Only 30 of the 180 control swabs were used in this study, limiting the validity of the comparison between the different lab methods. This limitation is highlighted by the remarkable difference in outcome between the laboratory methods when reviewing cases and controls for both FN and GAS.

A Letter to the Editor published in 2014 by Eaton et al. [59] concerned a project in which all throat swabs received by a microbiology laboratory in one year were cultured for GAS and FN, indicating that the only inclusion criterion was that a throat swab was taken. The laboratory served both PHC and secondary care, while the scope of this study was to focus on uncomplicated acute sore throat in PHC. Clinical details stated on accompanying request forms were used to determine if patients had pharyngitis. Those with either persistent

For peer review only - http://bmjopen.bmj.com/site/about/guidelines.xhtml

or recurrent symptoms were considered to have persistent sore throat syndrome (PSTS), indicating that multiple swabs from the same individual were allowed in the data. There was no information about antibiotic treatment. In conclusion, it was decided not to include the Letter to the Editor by Eaton et al. [59] in this review.

The text published in 2009 by Ludlam et al. [60] described a study design comparing cases from a local GP to controls comprising healthy university students, collecting throat swabs for both groups during the same two-month period. The description of inclusion and exclusion criteria for the cases was limited. The results for GAS, EBV, and FN were not shown for both groups, either in text or in tables. It is unclear whether the controls may become cases (and vice-versa). Their supplementary table contained information about antibiotic treatment in the control group, but not for the cases.

The article published in 2018 by Pallon et al. [61] was a follow-up study based on the same initial data already included in a previously published article by Hedin et al. [63], which is included in this review. Therefore, the article published in 2018 by Pallon et al. [61] was not included.

Methodological quality

Of the six studies included in the qualitative analysis, three were of overall high quality and one of medium quality [62-65] (Figure 2) and these were included in the meta-analysis.

Two studies presenting data from cases and controls were of low quality [66 67] and these were not included in the meta-analysis for the reasons described below.

The Danish study by Jensen et al. 2015 [67] examined the outcome of throat cultures arriving at a microbiologic laboratory. Most of the cases came from patients with 3-4 Centor criteria who had already tested negative with a rapid antigen detection test (RADT) for GAS. The control group consisted of subjects having a sore throat with 0-2 Centor criteria or fever

For peer review only - http://bmjopen.bmj.com/site/about/guidelines.xhtml

or who were screened for carriage of Staphylococcus aureus. None of the controls were screened using the above-mentioned RADT. The cases and controls were, therefore, deemed inappropriate for inclusion in the meta-analysis.

The article published in 2007 by Jensen et al. [66] had similar problems as those described above. The inclusion criteria were somewhat unclear. It appears as if primarily patients with a negative outcome of a RADT for GAS were included as cases. Hence, this study was also not included in the meta-analysis.

Presence of Fusobacterium necrophorum in patients with a sore throat

In high or medium quality articles, FN was detected in 18% of cases with a sore throat and a Centor score of 0-4, compared to 7% in healthy controls ($p<0.00001$, chi-square) (Table 2). The cumulative positive EPV for FN, including patients with 0-4 Centor scores, was 64% (95% CI 33-83%) (Table 3 and Figure 3). In cases with a Centor score of 3-4, FN was detected in 21% ($p<0.00001$, chi-square) (Table 2). The cumulative positive EPV regarding FN for patients with a Centor score of 3-4 was 71% (95% CI 34-88%) (Table 3 and Figure 4).

Fusobacterium necrophorum versus group A streptococcus

When including all cases (Centor score 0-4) in high to medium quality studies also providing data for GAS in the very same patients, the cumulative positive EPV for a finding of GAS was 93% (95% CI 83-99%) (Table 3 and Figure 5). In cases with a Centor score of 3-4, the positive EPV for GAS was 97% (95% CI 91-100%) (Table 3 and Figure 6).

Discussion

This literature review and meta-analysis showed that the P-EPV for FN (detected by culture or PCR) and the uncomplicated acute sore throat was 64% (95% CI 33-83%). The

For peer review only - http://bmjopen.bmj.com/site/about/guidelines.xhtml
For peer review only

corresponding P-EPV for GAS was 93% (95% CI 83-99%), based on data from the same publications and patients. When limiting the analyses to the patients with Centor score 3-4, the P-EPV for FN was 71% (95% CI 34-88%) and for GAS, 97% (95% CI 91-100%).

Strengths and limitations

A potential limitation is that there were only four available case-control studies presenting the proportion of FN. However, this study is the first systematic literature review with meta-analysis utilizing P-EPV to quantify the clinical relevance of a finding of FN in patients presenting with an acute uncomplicated sore throat in PHC. As such, it represents the current best understanding of the clinical importance of FN in patients with an uncomplicated acute sore throat.

The relatively high prevalence of FN in healthy controls (7.2%) indicates that FN, at least for some patients, is a part of the normal tonsillar flora. The proportion of patients with FN was 18% for Centor score 0-4 and 21% for Centor score 3-4, but the corresponding increase for GAS was from 19% to 35% (Table 2). Subsequently, the difference in the 95% confidence interval for P-EPV between patients having 0-4 versus 3-4 Centor scores was very small for FN, even if there was a marginal increase in the point estimate for P-EPV. A larger difference would be expected if FN is the main etiologic agent in a relevant proportion of patients.

The P-EPV numbers indicate that FN plays a role as a pathogen in patients with an uncomplicated acute sore throat. However, compared to the results for GAS, the association between FN and the uncomplicated acute sore throat appears to be considerably weaker and only marginally higher than the P-EPV of 53% (95% CI 36-67%) previously found for adults harboring group C streptococcus. [15] Furthermore, the narrow confidence intervals for GAS P-EPV are in contrast to the wide confidence intervals associated with the P-EPV for FN.

For peer review only - http://bmjopen.bmj.com/site/about/guidelines.xhtml

The high P-EPV for a finding of GAS in a throat swab is convincing and confirms the already well-established link between GAS and sore throat.[6]

It has been suggested that antibiotic treatment of uncomplicated acute sore throat caused by FN would be cost-effective if it reduces the incidence of Lemierre's syndrome by at least 20%. [26] However, this has not yet been investigated in a clinical trial, and it is unlikely it ever will be due to the very low incidence of Lemierre's syndrome. Other possible reasons for prescribing antibiotic treatment to patients with an uncomplicated acute sore throat caused by FN might be to shorten symptom duration or reduce the incidence of PTA. However, neither one has ever been tested in a clinical trial. Hence, although theoretically possible, we still have no proof that antibiotic treatment is beneficial to patients with an uncomplicated sore throat and presence of FN.

Conclusions and implications

For uncomplicated acute sore throat in PHC, the confidence interval of P-EPV for FN (33-83%) is wider and lower compared to the corresponding P-EPV for GAS (83-99%). The level of certainty for these confidence intervals are deemed as high as they are based on three high quality and one medium quality study including a total of 676 cases and 439 controls. Since the lower limit for the 95% confidence interval for FN is well above 0% we can with a high level of certainty state there is an association between FN and the uncomplicated acute sore throat in PHC. However, it is weaker than the same association for GAS.

We are not aware of any studies showing that antibiotic treatment has beneficial effects on the duration or severity of symptoms in an FN-associated acute sore throat. Nor do we have any evidence that antibiotic treatment to patients with an uncomplicated acute sore throat reduces the incidence of the life threatening Lemierre's syndrome. Hence, in the absence of this evidence we do not recommend routinely searching for FN in throat swabs or

For peer review only - http://bmjopen.bmj.com/site/about/guidelines.xhtml

prescribing antibiotics to patients with a GAS negative uncomplicated acute sore throat in PHC. However, to our knowledge at least one randomized controlled trial focusing on GAS-negative patients with a sore throat, and analysing the presence of FN, is underway. Hence, our current advice may in the future have to be revised.

More future studies should focus on randomizing patients with an uncomplicated acute sore throat and presence of only FN to treatment with antibiotics or placebo in order to assess whether the treatment is effective to reduce duration and intensity of symptoms, and, more importantly, if complications such as PTA can be prevented. The prevalence of Lemierre's syndrome is so low that any effect of antibiotics on its prevalence most likely would need to be estimated using other designs than a simple clinical trial comparing antibiotics with placebo.

Contributors

RG was responsible for conception of the idea. The overall design of the study was made by RG, SM, PDS, SP and KH. SM and SP were responsible for assessing the eligibility and extract data from publications. Statistical analysis, interpretation of results and writing of manuscript, was made by all authors (RG, SM, PDS, SP and KH). All authors (RG, SM, PDS, SP and KH) approved the final version of the manuscript.

Data sharing statement

The raw data files are available from the corresponding author upon request.

Competing interest statement

None to declare.

For peer review only - http://bmjopen.bmj.com/site/about/guidelines.xhtml

For peer review only

Acknowledgements

Funding for this project came from the Local Research and Development Council, Södra Älvsborg, Sweden (reference number VGFOUSA-937533). Funding was also received from Research and Development Center for Primary Health Care, Borås, Sweden (reference number VGFOUSA-P-872381).

The authors would like to thank Professor Robert Centor who willingly provided further information from his study "The clinical presentation of Fusobacterium-positive and streptococcal-positive pharyngitis in a university health clinic: a cross-sectional study" [62], enabling us to calculate separate P-EPV for patients with 0-4 and 3-4 Centor criteria.

For peer review only - http://bmjopen.bmj.com/site/about/guidelines.xhtml

References

1. André M, Vernby Å, Odenholt I, et al. Diagnosis-prescribing surveys in 2000, 2002 and 2005 in Swedish general practice: Consultations, diagnosis, diagnostics and treatment choices. *Scandinavian Journal of Infectious Diseases* 2008(40):648- 54. doi: 10.1080/00365540801932439

2. Tyrstrup M, Beckman A, Molstad S, et al. Reduction in antibiotic prescribing for respiratory tract infections in Swedish primary care- a retrospective study of electronic patient records.(Report). *BMC Infectious Diseases* 2016;16(1) doi: 10.1186/s12879-016-2018-9

3. Armstrong GL, Pinner RW. Outpatient visits for infectious diseases in the United States, 1980 through 1996. *Arch Intern Med* 1999;159(21):2531-6. doi: 10.1001/archinte.159.21.2531 [published Online First: 1999/11/26]

4. Shulman ST, Bisno AL, Clegg HW, et al. Clinical practice guideline for the diagnosis and management of group A streptococcal pharyngitis: 2012 update by the Infectious Diseases Society of America. *Clinical infectious diseases : an official publication of the Infectious Diseases Society of America* 2012;55(10):e86. doi: 10.1093/cid/cis629

5. Centor RM, Witherspoon JM, Dalton HP, et al. The diagnosis of strep throat in adults in the emergency room. *Medical decision making : an international journal of the Society for Medical Decision Making* 1981;1(3):239.

6. Pelucchi C, Grigoryan L, Galeone C, et al. Guideline for the management of acute sore throat. Oxford, UK, 2012:1-28.

7. Marchello C, Ebell MH. Prevalence of Group C Streptococcus and Fusobacterium Necrophorum in Patients With Sore Throat: A Meta-Analysis. *Ann Fam Med* 2016;14(6):567-74. doi: 10.1370/afm.2005 [published Online First: 2017/04/05]

For peer review only - http://bmjopen.bmj.com/site/about/guidelines.xhtml

8. Klug TE, Rusan M, Fuursted K, et al. A systematic review of Fusobacterium necrophorum-positive acute tonsillitis: prevalence, methods of detection, patient characteristics, and the usefulness of the Centor score. *European journal of clinical microbiology & infectious diseases : official publication of the European Society of Clinical Microbiology* 2016;35(12):1903-12. doi: 10.1007/s10096-016-2757-y [published Online First: 2016/08/29]

9. Holm K, Bank S, Nielsen H, et al. The role of Fusobacterium necrophorum in pharyngotonsillitis - A review. *Anaerobe* 2016;42:89-97. doi: 10.1016/j.anaerobe.2016.09.006

10. Riordan T. Human infection with Fusobacterium necrophorum (Necrobacillosis), with a focus on Lemierre's syndrome. *Clinical microbiology reviews* 2007;20(4):622-59. doi: 10.1128/cmr.00011-07 [published Online First: 2007/10/16]

11. Wikstén J, Kaltiainen E, Pitkäranta A, et al. Renewal of peritonsillar abscess: Impact of the bacterial species of the infection and clinical features of the patient—A prospective comparative aetiological study. *Clinical Otolaryngology* 2017;42(6):1358-62. doi: 10.1111/coa.12893

12. Powell EL, Powell J, Samuel JR, et al. A review of the pathogenesis of adult peritonsillar abscess: time for a re-evaluation. *Journal of Antimicrobial Chemotherapy* 2013;68(9):1941-50. doi: 10.1093/jac/dkt128

13. Moher D, Liberati A, Tetzlaff J, et al. Preferred reporting items for systematic reviews and meta-analyses: the PRISMA statement. *Annals of internal medicine* 2009;151(4):264. doi: 10.7326/0003-4819-151-4-200908180-00135

14. Gunnarsson RK, Lanke J. The predictive value of microbiologic diagnostic tests if asymptomatic carriers are present. *Statistics in Medicine* 2002;21(12):1773-85. doi: 10.1002/sim.1119

For peer review only - http://bmjopen.bmj.com/site/about/guidelines.xhtml

15. Gunnarsson RK, Manchal N. Group C beta hemolytic Streptococci as a potential pathogen in patients presenting with an uncomplicated acute sore throat - a systematic literature review and meta-analysis. *Scand J Prim Health Care* 2020:1-12. doi: 10.1080/02813432.2020.1753374 [published Online First: 2020/05/05]

16. von Hippel PT. The heterogeneity statistic I(2) can be biased in small meta-analyses. *BMC Med Res Methodol* 2015;15:35. doi: 10.1186/s12874-015-0024-z [published Online First: 2015/04/17]

17. Bank S, Nielsen HM, Hoyer Mathiasen B, et al. Fusobacterium necrophorum- detection and identification on a selective agar. *APMIS* 2010;118(12):994-99. doi: 10.1111/j.1600-0463.2010.02683.x

18. Tejesvi MV, Uhari M, Tapiainen T, et al. Tonsillar microbiota in children with PFAPA (periodic fever, aphthous stomatitis, pharyngitis, and adenitis) syndrome. *European Journal of Clinical Microbiology and Infectious Diseases* 2016;35(6):963-70. doi: 10.1007/s10096-016-2623-y

19. Bailhache M, Mariani-Kurkdjian P, Lehours P, et al. Fusobacterium invasive infections in children: A retrospective study in two French tertiary care centres. *European Journal of Clinical Microbiology and Infectious Diseases* 2013;32(8):1041-47. doi: 10.1007/s10096-013-1848-2

20. Goldenberg NA, Knapp-Clevenger R, Hays T, et al. Lemierre's and Lemierre's-like syndromes in children: survival and thromboembolic outcomes. *Pediatrics* 2005;116(4):e543-8. doi: 10.1542/peds.2005-0433 [published Online First: 2005/09/09]

21. Papaparaskevas J, Katsandri A, Pantazatou A, et al. Epidemiological characteristics of infections caused by Bacteroides, Prevotella and Fusobacterium species: A prospective observational study. *Anaerobe* 2011;17(3):113-17. doi: 10.1016/j.anaerobe.2011.05.013

For peer review only - http://bmjopen.bmj.com/site/about/guidelines.xhtml

22. Ramirez S, Hild TG, Rudolph CN, et al. Increased diagnosis of Lemierre syndrome and other Fusobacterium necrophorum infections at a Children's Hospital. *Pediatrics* 2003;112(5):e380. [published Online First: 2003/11/05]

23. Rusan M, Klug TE, Ovesen T. An overview of the microbiology of acute ear, nose and throat infections requiring hospitalisation. *European journal of clinical microbiology & infectious diseases : official publication of the European Society of Clinical Microbiology* 2009;28(3):243-51. doi: 10.1007/s10096-008-0619-y [published Online First: 2008/10/03]

24. Yusuf E, Halewyck S, Wybo I, et al. Fusobacterium necrophorum and other Fusobacterium spp. isolated from head and neck infections: A 10-year epidemiology study in an academic hospital. *Anaerobe* 2015;34:120-4. doi: 10.1016/j.anaerobe.2015.05.006 [published Online First: 2015/05/20]

25. Albertz N, Nazar G. Peritonsillar abscess: treatment with immediate tonsillectomy - 10 years of experience. *Acta Otolaryngol* 2012;132(10):1102-7. doi: 10.3109/00016489.2012.684399 [published Online First: 2012/06/20]

26. Bank S, Christensen K, Kristensen LH, et al. A cost-effectiveness analysis of identifying Fusobacterium necrophorum in throat swabs followed by antibiotic treatment to reduce the incidence of Lemierre's syndrome and peritonsillar abscesses. *Eur J Clin Microbiol Infect Dis* 2013;32(1):71-8. doi: 10.1007/s10096-012-1715-6

27. Batty A, Wren MW, Gal M. Fusobacterium necrophorum as the cause of recurrent sore throat: comparison of isolates from persistent sore throat syndrome and Lemierre's disease. *J Infect* 2005;51(4):299-306. doi: 10.1016/j.jinf.2004.09.013 [published Online First: 2005/07/30]

For peer review only - http://bmjopen.bmj.com/site/about/guidelines.xhtml

For peer review only

28. Batty A, Wren MWD. Prevalence of Fusobacterium necrophorum and other upper respiratory tract pathogens isolated from throat swabs. *British Journal of Biomedical Science* 2005;62(2):66-70. doi: 10.1080/09674845.2005.11732687

29. Bjork H, Bieber L, Hedin K, et al. Tonsillar colonisation of Fusobacterium necrophorum in patients subjected to tonsillectomy. *BMC Infect Dis* 2015;15:264. doi: 10.1186/s12879-015-0975-z [published Online First: 2015/07/15]

30. Brook I. Aerobic and anaerobic bacteriology of peritonsillar abscess in children. *Acta paediatrica Scandinavica* 1981;70(6):831-5. [published Online First: 1981/11/01]

31. Brook I. Role of anaerobic beta-lactamase-producing bacteria in upper respiratory tract infections. *Pediatric Infectious Disease Journal* 1987;6(3):310-16. doi: 10.1097/00006454-198703000-00045

32. Brook I, Foote Jr PA, Slots J. Immune response to anaerobic bacteria in patients with peritonsillar cellulitis and abscess. *Acta Oto-Laryngologica* 1996;116(6):888-91. doi: 10.3109/00016489609137946

33. Brook I, Shah K, Jackson W. Microbiology of healthy and diseased adenoids. *Laryngoscope* 2000;110(6):994-9. doi: 10.1097/00005537-200006000-00021 [published Online First: 2000/06/14]

34. Dapefrid A, Lundström B, Tano K. Prevalence of Fusobacterium necrophorum in tonsils from patients with chronic tonsillitis. *Acta Oto-Laryngologica* 2017;137(3):297-301. doi: 10.1080/00016489.2016.1229024

35. Develioglu ON, Ipek HD, Bahar H, et al. Bacteriological evaluation of tonsillar microbial flora according to age and tonsillar size in recurrent tonsillitis. *European Archives of Oto-Rhino-Laryngology* 2014;271(6):1661-65. doi: 10.1007/s00405-014-2898-5

36. Ehlers Klug T, Rusan M, Fuursted K, et al. Fusobacterium necrophorum: most prevalent pathogen in peritonsillar abscess in Denmark. *Clinical infectious diseases : an official*

For peer review only

For peer review only - http://bmjopen.bmj.com/site/about/guidelines.xhtml

publication of the Infectious Diseases Society of America 2009;49(10):1467-72. doi: 10.1086/644616 [published Online First: 2009/10/22]

37. Hagelskjaer Kristensen L, Prag J. Localised Fusobacterium necrophorum infections: a prospective laboratory-based Danish study. *Eur J Clin Microbiol Infect Dis* 2008;27(8):733-9. doi: 10.1007/s10096-008-0497-3

38. Hagelskjær Kristensen L, Prag J. Lemierre's syndrome and other disseminated Fusobacterium necrophorum infections in Denmark: A prospective epidemiological and clinical survey. *European Journal of Clinical Microbiology and Infectious Diseases* 2008;27(9):779-89. doi: 10.1007/s10096-008-0496-4

39. Hayakawa K, Nagashima M, Ohta K, et al. Fusobacterium necrophorum subsp. funduliforme in tonsils from various patient populations in Japan. *Japanese journal of infectious diseases* 2018 doi: 10.7883/yoken.JJID.2018.061 [published Online First: 2018/06/01]

40. Holm K, Svensson PJ, Rasmussen M. Invasive Fusobacterium necrophorum infections and Lemièrre's syndrome: the role of thrombophilia and EBV. *European Journal of Clinical Microbiology and Infectious Diseases* 2015;34(11):2199-207. doi: 10.1007/s10096-015-2469-8

41. Horn J, Bender BS, Bartlett JG. Role of anaerobic bacteria in perimandibular space infections. *The Annals of otology, rhinology & laryngology Supplement* 1991;154:34-39.

42. Huggan PJ, Murdoch DR. Fusobacterial infections: clinical spectrum and incidence of invasive disease. *J Infect* 2008;57(4):283-9. doi: 10.1016/j.jinf.2008.07.016 [published Online First: 2008/09/23]

43. Jensen A, Fago-Olsen H, Sorensen CH, et al. Molecular mapping to species level of the tonsillar crypt microbiota associated with health and recurrent tonsillitis. *PLoS One*

For peer review only - http://bmjopen.bmj.com/site/about/guidelines.xhtml

2013;8(2):e56418. doi: 10.1371/journal.pone.0056418 [published Online First: 2013/02/26]

44. Jokipii AMM, Jokipii L, Sipila P, et al. Semiquantitative culture results and pathogenic significance of obligate anaerobes in peritonsillar abscesses. *Journal of Clinical Microbiology* 1988;26(5):957-61.

45. Klug TE. Incidence and microbiology of peritonsillar abscess: the influence of season, age, and gender. *European journal of clinical microbiology & infectious diseases : official publication of the European Society of Clinical Microbiology* 2014;33(7):1163-7. doi: 10.1007/s10096-014-2052-8 [published Online First: 2014/01/30]

46. Klug TE, Fischer AS, Antonsen C, et al. Parapharyngeal abscess is frequently associated with concomitant peritonsillar abscess. *Eur Arch Otorhinolaryngol* 2014;271(6):1701-7. doi: 10.1007/s00405-013-2667-x [published Online First: 2013/08/29]

47. Klug TE, Henriksen JJ, Fuursted K, et al. Significant pathogens in peritonsillar abscesses. *European journal of clinical microbiology & infectious diseases : official publication of the European Society of Clinical Microbiology* 2011;30(5):619-27. doi: 10.1007/s10096-010-1130-9 [published Online First: 2010/12/25]

48. Lepelletier D, Pinaud V, Le Conte P, et al. Is there an association between prior anti-inflammatory drug exposure and occurrence of peritonsillar abscess (PTA)? A national multicenter prospective observational case–control study. *European Journal of Clinical Microbiology and Infectious Diseases* 2017;36(1):57-63. doi: 10.1007/s10096-016-2770-1

49. Mitchelmore IJ, Prior AJ, Montgomery PQ, et al. Microbiological features and pathogenesis of peritonsillar abscesses. *European journal of clinical microbiology & infectious diseases : official publication of the European Society of Clinical Microbiology* 1995;14(10):870-7. [published Online First: 1995/10/01]

For peer review only

For peer review only - http://bmjopen.bmj.com/site/about/guidelines.xhtml

50. Price SL, Hardy S, Gale P, et al. Prevalence of Fusobacterium necrophorum in persistent sore throat samples. *Br J Biomed Sci* 2011;68(4):209-10. [published Online First: 2012/01/24]

51. Tachibana T, Orita Y, Takao S, et al. The role of bacteriological studies in the management of peritonsillar abscess. *Auris Nasus Larynx* 2016;43(6):648-53. doi: 10.1016/j.anl.2016.01.009

52. Wiksten JE, Laakso S, Maki M, et al. Microarray identification of bacterial species in peritonsillar abscesses. *European journal of clinical microbiology & infectious diseases : official publication of the European Society of Clinical Microbiology* 2015;34(5):905-11. doi: 10.1007/s10096-014-2301-x [published Online First: 2014/12/31]

53. Amess JA, O'Neill W, Ni Giollariabhaigh C, et al. A six-month audit of the isolation of Fusobacterium necrophorum from patients with sore throat in a district general hospital. *British Journal of Biomedical Science* 2007;64(2):63-65. doi: 10.1080/09674845.2007.11732757

54. Suzuki K, Kurono Y, Ikeda K, et al. Nationwide surveillance of 6 otorhinolaryngological infectious diseases and antimicrobial susceptibility pattern in the isolated pathogens in Japan. *Journal of Infection and Chemotherapy* 2015;21(7):483-91. doi: 10.1016/j.jiac.2015.03.005

55. Van TT, Cox LM, Cox ME, et al. Prevalence of Fusobacterium necrophorum in Children Presenting with Pharyngitis. *J Clin Microbiol* 2017;55(4):1147-53. doi: 10.1128/jcm.02174-16 [published Online First: 2017/01/27]

56. Windfuhr JP, Toepfner N, Steffen G, et al. Clinical practice guideline: tonsillitis I. Diagnostics and nonsurgical management. *European Archives of Oto-Rhino-Laryngology* 2016;273(4):973-87. doi: 10.1007/s00405-015-3872-6

For peer review only

For peer review only - http://bmjopen.bmj.com/site/about/guidelines.xhtml

57. Aliyu SH, Marriott RK, Curran MD, et al. Real-time PCR investigation into the importance of Fusobacterium necrophorum as a cause of acute pharyngitis in general practice. *J Med Microbiol* 2004;53(Pt 10):1029-35. doi: 10.1099/jmm.0.45648-0 [published Online First: 2004/09/11]

58. Atkinson TP, Centor RM, Xiao L, et al. Analysis of the tonsillar microbiome in young adults with sore throat reveals a high relative abundance of Fusobacterium necrophorum with low diversity. *PLoS One* 2018;13(1):e0189423. doi: 10.1371/journal.pone.0189423 [published Online First: 2018/01/20]

59. Eaton C, Swindells J. The significance and epidemiology of Fusobacterium necrophorum in sore throats. *J Infect* 2014;69(2):194-6. doi: 10.1016/j.jinf.2014.03.005 [published Online First: 2014/03/20]

60. Ludlam H, Howard J, Kingston B, et al. Epidemiology of pharyngeal carriage of Fusobacterium necrophorum. *J Med Microbiol* 2009;58(Pt 9):1264-5. doi: 10.1099/jmm.0.006718-0 [published Online First: 2009/06/17]

61. Pallon J, Sundqvist M, Hedin K. A 2-year follow-up study of patients with pharyngotonsillitis. *BMC Infect Dis* 2018;18(1):3. doi: 10.1186/s12879-017-2917-4 [published Online First: 2018/01/03]

62. Centor RM, Atkinson TP, Ratliff AE, et al. The clinical presentation of Fusobacterium-positive and streptococcal-positive pharyngitis in a university health clinic: a cross-sectional study. *Ann Intern Med* 2015;162(4):241-7. doi: 10.7326/m14-1305 [published Online First: 2015/02/17]

63. Hedin K, Bieber L, Lindh M, et al. The aetiology of pharyngotonsillitis in adolescents and adults - Fusobacterium necrophorum is commonly found. *Clinical microbiology and infection : the official publication of the European Society of Clinical Microbiology and*

For peer review only

For peer review only - http://bmjopen.bmj.com/site/about/guidelines.xhtml

Infectious Diseases 2015;21(3):263.e1-7. doi: 10.1016/j.cmi.2014.08.020 [published Online First: 2015/02/07]

64. Kjaerulff AM, Thomsen MK, Ovesen T, et al. Clinical and biochemical characteristics of patients with Fusobacterium necrophorum-positive acute tonsillitis. *Eur Arch Otorhinolaryngol* 2015;272(6):1457-63. doi: 10.1007/s00405-015-3535-7 [published Online First: 2015/04/16]

65. Hayakawa K, Nagashima M, Kanehisa E, et al. Real-time PCR investigation of the prevalence of Fusobacterium necrophorum in patients with pharyngitis in Japan. *J Infect Chemother* 2018;24(12):969-74. doi: 10.1016/j.jiac.2018.09.008 [published Online First: 2018/10/15]

66. Jensen A, Hagelskjær Kristensen L, Prag J. Detection of fusobacterium necrophorum subsp. funduliforme in tonsillitis in young adults by real-time PCR. *Clinical Microbiology and Infection* 2007;13(7):695-701. doi: 10.1111/j.1469-0691.2007.01719.x

67. Jensen A, Hansen TM, Bank S, et al. Fusobacterium necrophorum tonsillitis: an important cause of tonsillitis in adolescents and young adults. *Clinical microbiology and infection : the official publication of the European Society of Clinical Microbiology and Infectious Diseases* 2015;21(3):266.e1-3. doi: 10.1016/j.cmi.2014.09.020 [published Online First: 2015/02/07]

For peer review only

For peer review only - http://bmjopen.bmj.com/site/about/guidelines.xhtml

Table 1- Methodological quality assessment of included studies

	Low risk	Intermediate risk	High risk
**Definition of cases**	Cases well defined as per Centor criteria or similar.	At least two criteria mentioned in case definition.	Cases not defined.
**Healthy controls**	Study includes comparison with asymptomatic controls.	Controls not asymptomatic.	---
**Swab method**	Area of throat swabbed described, transport and storage mentioned.	Area of throat swabbed mentioned but not the transport or storage.	No mention of swab method.
**Culture method**	Clear description of culture media, incubation time (or PCR if used).	Method described but not in detail	Method not discussed
**Type of study**	Case control studies on FN	Community surveillance studies mentioning FN prevalence	Observational studies without well-defined cases and controls
**Same area and time period**	Cases and controls are collected in the same area and time of year	Cases and controls are collected in the same area but over different time periods	Cases and controls are collected in different regions and time periods

For peer review only

For peer review only - http://bmjopen.bmj.com/site/about/guidelines.xhtml

Table 2 – Case-control studies examining Fusobacterium necrophorum and group A streptococcus in patients with an acute uncomplicated sore throat in primary care

Study [ref]	Design	Method	Age, years (range)		Number of cases and controls			% FN detected (number)			% GAS detected (number)		
			Cases	Controls	Cases		Controls	Cases		Controls	Cases		Controls
					Centor 0-4	Centor 3-4		Centor 0-4	Centor 3-4		Centor 0-4	Centor 3-4	
Hedin 2015 [63]	Pro	Culture	33 (15–48)	31 (16-46)	220	85	128	15% (33)	19% (16)	3.1% (4)	30% (66)	49% (42)	2.3% (3)
Centor 2015 [62]	Pro	PCR	22 (15-30)	24 (15-30)	312	64	180	21% (64)	23% (15)	9.4% (17)	10% (32)	16% (10)	1.1% (2)
Kjærulff 2015 [64]	Pro	Culture	28 (15-40)	29 (15-40)	100	29	100	16% (16)	21% (6)	9.0 % (9)	26% (26)	48% (14)	3.0% (3)
Hayakawa 2018 [65]	Pro	PCR + culture	29 (25-37)	33 (26-36)	44	19	31	14% (6)	21% (4)	6.5% (2)	11% (5)	16% (3)	0.0% (0)
SUBTOTAL (HIGH & MEDIUM QUALITY)					***676***	***197***	***439***	***18% (119)***	***21% (41)***	***7.2% (32)***	***19% (129)***	***35% (69)***	***1.8(8)***
Jensen 2007 [66]	Pro	PCR + culture	25 (18-32)	22 (18-32)	105	-	92	51% (54)	-	21% (19)	5.7%* (6*)	-	-
Jensen 2015 [67]	Retro	Culture	19 (10-40)	22 (10-40)	179	-	176	24% (43)	-	5.7% (10)	3.9%* (7*)	-	0% (0)
SUBTOTAL (LOW QUALITY ARTICLES)					*284*	-	*268*	*34% (97)*	-	*11% (29)*	*4.6%* (13*)*	-	-
TOTAL (ALL SIX ARTICLES)					960	178	707	23% (216)	21% (37)	8.6% (61)	15%* (142*)	37% (66)	1.1% (8)

*GAS-tonsillitis was excluded (by GPs using rapid antigen tests) in the two articles by Jensen et al.; thus, those results for GAS were irrelevant for the purpose of this meta-analysis.

For peer review only - http://bmjopen.bmj.com/site/about/guidelines.xhtml

Table 3 – Positive etiologic predictive value (P-EPV)*

Study	Fusobacterium necrophorum Centor 0-4**	Fusobacterium necrophorum Centor 3-4**	Group A streptococcus Centor 0-4**	Group A streptococcus Centor 3-4**
Hedin 2015 (Sweden)	82% (34-100)	86% (32-100)	95% (82-100)	98% (91-100)
Centor 2015 (US)	60% (8.2-87)	67% (0.0-92)	90% (57-100)	94% (49-100)
Kjaerulff 2015 Denmark)	49% (0.0-92)	63% (0.0-96)	93% (71-100)	97% (81-100)
Hayakawa 2018 (Japan)	57% (0.0-100)	76% (0.0-100)	-----	-----
All studies combined	64% (33-83)	71% (34-88)	93% (83-99)	97% (91-100)

* P-EPV is a method of quantifying the probability of a true link between symptoms and signs (a sore throat) and the finding of a bacterium in the throat while considering the possibility of healthy carriers of the same bacterium. 0% indicates no probability for a true link and 100% indicates a certain link.

** Including either all patients with a sore throat (Centor criteria 0-4) or only those with more prominent symptoms (Centor score 3-4).

For peer review only

For peer review only - http://bmjopen.bmj.com/site/about/guidelines.xhtml

Figure 1 – PRISMA Flow Diagram

Figure 2 – Quality assessment of included studies

Figure 3 - Probability of a true link between sore throat with Centor score 0-4 and positive FN test

*Positive etiologic predictive value is the probability of a true link between sore throat and FN based on studies with data from both patients and healthy controls.

Figure 4 - Probability of a true link between the sore throat with Centor score 3-4 and positive FN test

*Positive etiologic predictive value is the probability of a true link between sore throat and FN based on studies with data from both patients and healthy controls.

For peer review only

For peer review only - http://bmjopen.bmj.com/site/about/guidelines.xhtml

Figure 5 - Probability of a true link between sore throat with Centor score 0-4 and positive GAS test

*Positive etiologic predictive value is the probability of a true link between sore throat and GAS based on studies with data from both patients and healthy controls.

Since the study by Hayakawa 2018 found no positive tests for GAS among healthy controls, P-EPV could not be calculated for that study separately. However, the results from the four studies selected for meta-analysis are included in the summary.

Figure 6 - Probability of a true link between sore throat with Centor score 3-4 and positive GAS test

*Positive etiologic predictive value is the probability of a true link between sore throat and GAS based on studies with data from both patients and healthy controls.
Since the study by Hayakawa 2018 found no positive tests for GAS among healthy controls, P-EPV could not be calculated for that study separately. However, the results from the four studies selected for meta-analysis are included in the summary.

For peer review only

For peer review only - http://bmjopen.bmj.com/site/about/guidelines.xhtml

Acute Sore Throat and Fusobacterium Necrophorum in PHC – A Systematic Review and Meta-analysis
Version: (15/7/20 12:12:00 pm)

PRISMA 2009 Flow Diagram

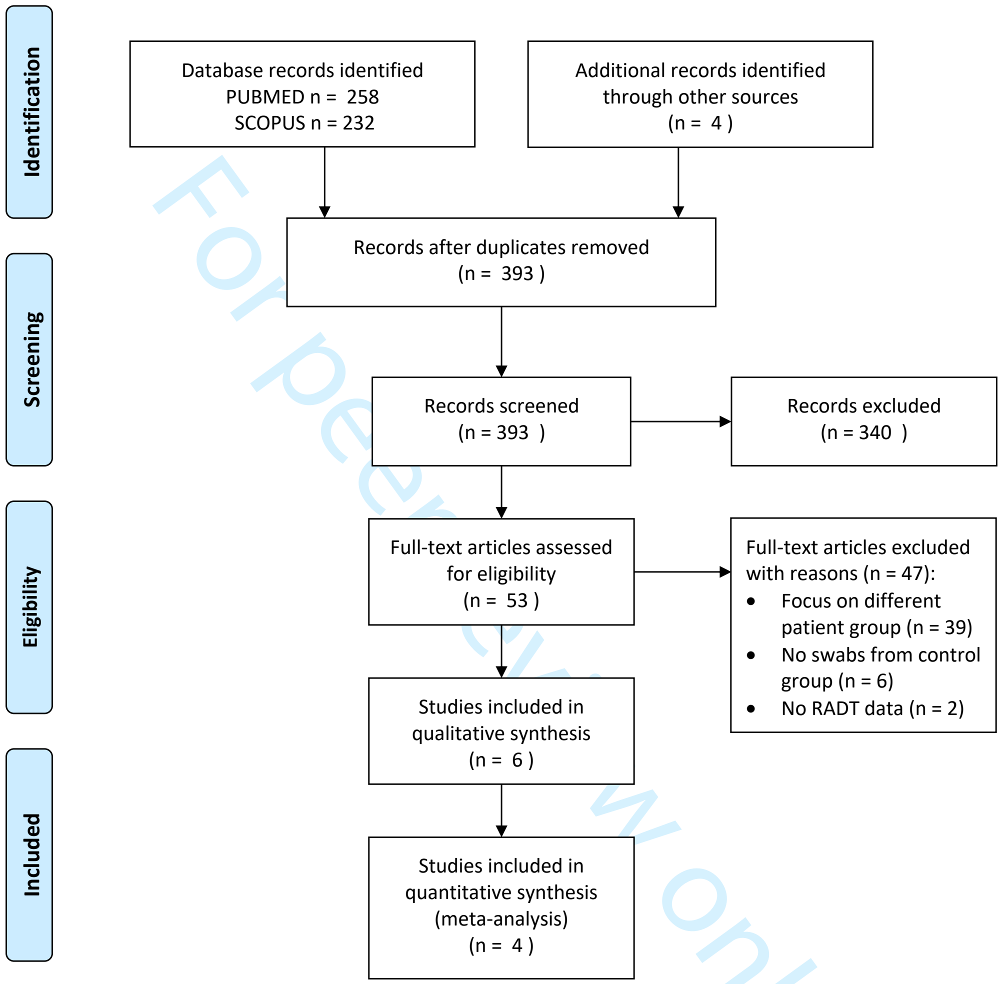

From: Moher D, Liberati A, Tetzlaff J, Altman DG, The PRISMA Group (2009). *P*referred *R*eporting *I*tems for *S*ystematic Reviews and *M*eta-*A*nalyses: The PRISMA Statement. PLoS Med 6(7): e1000097. doi:10.1371/journal.pmed1000097

For more information, visit www.prisma-statement.org.

Figure 1- PRISMA Flow Diagram

For peer review only - http://bmjopen.bmj.com/site/about/guidelines.xhtml

Study	Case defined	Healthy controls	Swab method	Culture method	Type of study	Same geo-graphical area, time
Hedin, 2015	+ (green)	+ (green)	+ (green)	+ (green)	+ (green)	+ (green)
Centor, 2015	+ (green)	+ (green)	+ (green)	+ (green)	+ (green)	+ (green)
Kjærulf, 2015	+ (green)	+ (green)	+ (green)	+ (green)	+ (green)	+ (green)
Hayakawa, 2018	+ (yellow)	+ (green)	+ (green)	+ (green)	+ (green)	+ (green)
Jensen, 2007	+ (yellow)	+ (green)	+ (red)	+ (green)	+ (green)	+ (yellow)
Jensen, 2015	+ (green)	+ (yellow)	+ (red)	+ (yellow)	+ (red)	+ (green)

Figure 2 - Quality assessment of included studies

+ (green) Low risk of bias + (yellow) Intermediate risk of bias + (red) High risk of bias

For peer review only

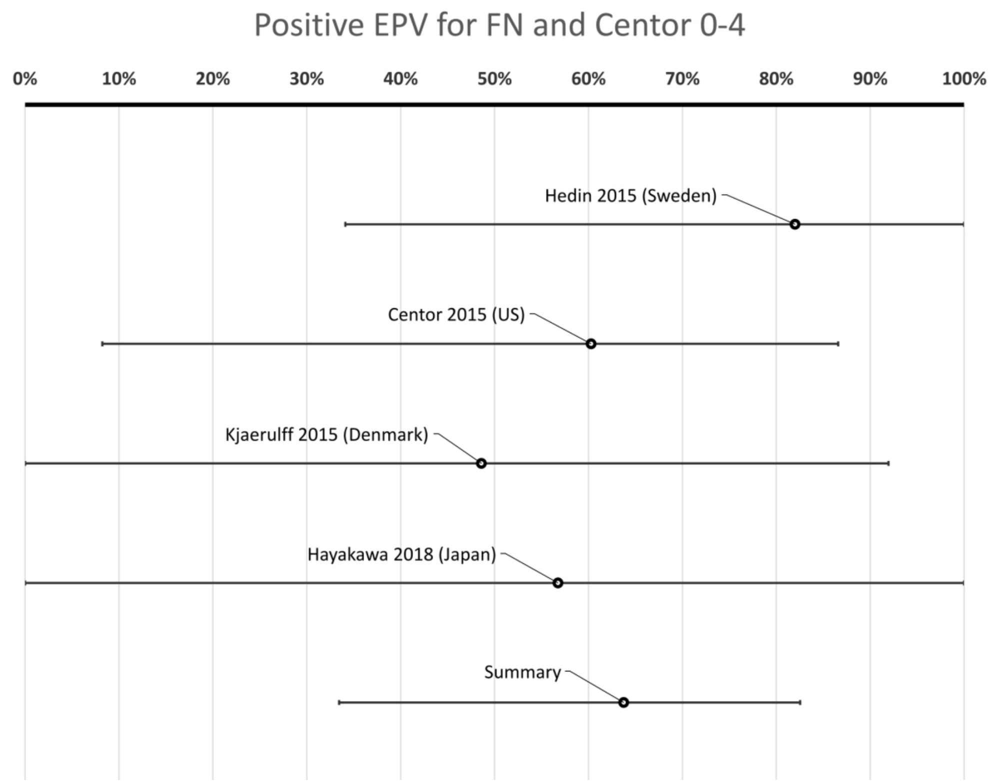

Figure 3 - Probability of a true link between sore throat with Centor score 0-4 and positive FN test

176x137mm (300 x 300 DPI)

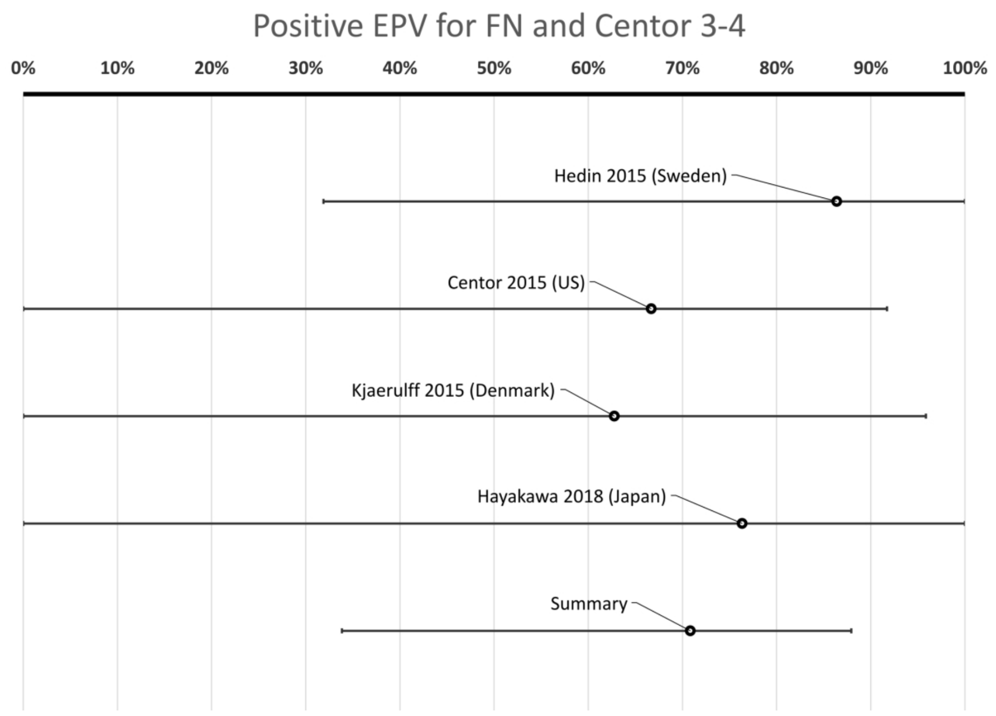

Figure 4 - Probability of a true link between the sore throat with Centor score 3-4 and positive FN test

175x125mm (300 x 300 DPI)

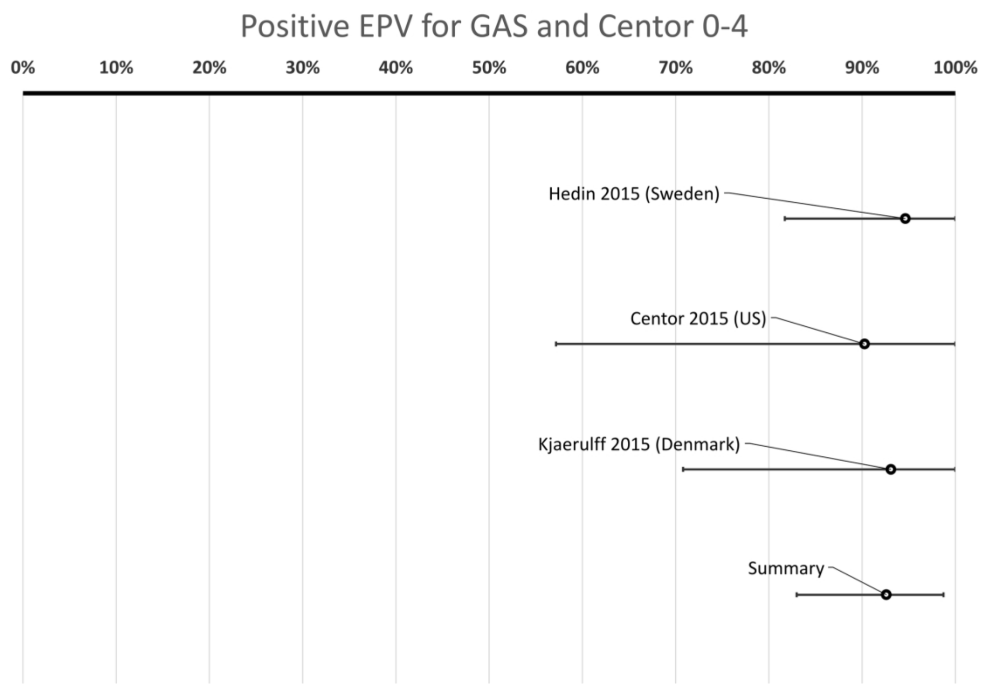

Figure 5 - Probability of a true link between sore throat with Centor score 0-4 and positive GAS test

178x121mm (300 x 300 DPI)

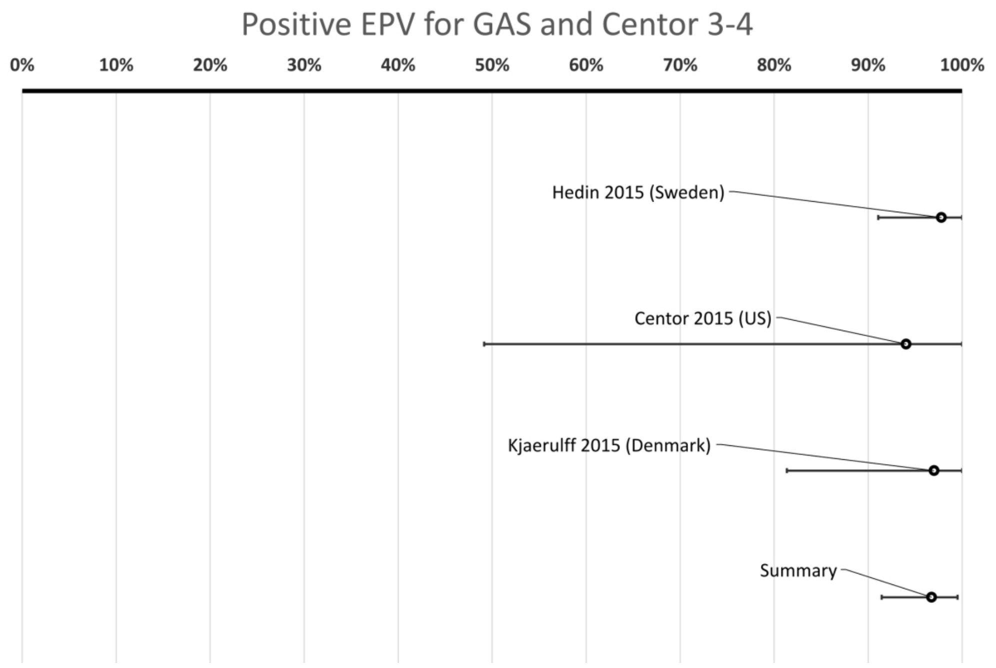

Figure 6 - Probability of a true link between sore throat with Centor score 3-4 and positive GAS test

176x117mm (300 x 300 DPI)

Acute Sore Throat and Fusobacterium Necrophorum in PHC – A Systematic Review and Meta-analysis - Appendix 1 and 2
Version: (11 January 2021)

Appendix 1: Search strings for PubMed and PROSPERO

PubMed:

(("pharyngitis"[MH] OR "pharyngitis"[TIAB] OR "Lemierre Syndrome"[MH] OR "Lemierre Syndrome"[TIAB] OR "Necrobacillosis"[TIAB] OR "pharyngotonsillitis "[TIAB] OR "tonsillitits"[TIAB] OR "throat"[TIAB] OR "epidemiology"[TIAB])

AND ("fusobacterium necrophorum"[MH] OR "fusobacterium"[TIAB]))

NOT "Case Reports"[pt]

Scopus:

(TITLE-ABS-KEY ((pharyngitis OR "Lemierre Syndrome" OR necrobacillosis OR pharyngotonsillitis OR tonsillitis OR "Peritonsillar Abscess" OR throat OR epidemiology))

AND TITLE-ABS-KEY (fusobacterium))

AND DOCTYPE (ar)

AND (LIMIT-TO (SUBJAREA , "MEDI ") OR LIMIT-TO (SUBJAREA , " IMMU ")

OR LIMIT-TO (SUBJAREA , " BIO "))

AND (LIMIT-TO (LANGUAGE , "English "))

AND (EXCLUDE (EXACTKEYWORD , "Case Report"))

For peer review only - http://bmjopen.bmj.com/site/about/guidelines.xhtml

Acute Sore Throat and Fusobacterium Necrophorum in PHC – A Systematic Review and Meta-analysis - Appendix 1 and 2
Version: (11 January 2021)

Appendix 2: Formulae for calculating P-EPV

Formula for the point estimate of P-EPV:

$$P(\mathrm{D}^+|\mathrm{S}^+\mathrm{T}^+) = 1 - \left(\frac{\dfrac{\mathrm{Sen}}{P(\mathrm{T}^+|\mathrm{S}^+)} - 1}{\dfrac{\mathrm{Sen}}{P(\mathrm{T}^+|\mathrm{S}^+\mathrm{D}^-)} - 1} \right)$$

Formula for the confidence interval of P-EPV:

$$\left(1 - \left(\frac{\dfrac{\mathrm{Sen}}{a} - 1}{\dfrac{\mathrm{Sen}}{d} - 1} \right) \right), \left(1 - \left(\frac{\dfrac{\mathrm{Sen}}{b} - 1}{\dfrac{\mathrm{Sen}}{c} - 1} \right) \right)$$

$$a = p(\mathrm{T}^+|\mathrm{S}^+) - \left(Z_{\alpha/4} \times \sqrt{\frac{p(\mathrm{T}^+|\mathrm{S}^+) \times (1 - p(\mathrm{T}^+|\mathrm{S}^+))}{\#(\mathrm{S}^+)}} \right)$$

$$b = p(\mathrm{T}^+|\mathrm{S}^+) + \left(Z_{\alpha/4} \times \sqrt{\frac{p(\mathrm{T}^+|\mathrm{S}^+) \times (1 - p(\mathrm{T}^+|\mathrm{S}^+))}{\#(\mathrm{S}^+)}} \right)$$

$$c = p(\mathrm{T}^+|\mathrm{S}^+\mathrm{D}^-) - \left(Z_{\alpha/4} \times \theta \times \sqrt{\frac{p(\mathrm{T}^+|\mathrm{S}^-) \times (1 - p(\mathrm{T}^+|\mathrm{S}^-))}{\#(\mathrm{S}^-)}} \right)$$

$$d = p(\mathrm{T}^+|\mathrm{S}^+\mathrm{D}^-) + \left(Z_{\alpha/4} \times \theta \times \sqrt{\frac{p(\mathrm{T}^+|\mathrm{S}^-) \times (1 - p(\mathrm{T}^+|\mathrm{S}^-))}{\#(\mathrm{S}^-)}} \right)$$

Definitions:
T = Test outcome which may be positive (T^+) or negative (T^-)
S = Symptoms of a sore throat which may be present (S^+) or absent (S^-)
M = The bacteria of interest (FN or GAS) which may be present (M^+) or absent (M^-)
D = Disease (a sore throat caused by FN/GAS) which may be present (D^+) or absent (D^-)
Sensitivity of test to detect bacteria = $\mathrm{Sen} = P(\mathrm{T}^+|\mathrm{M}^+)$

Setting the width of the confidence interval. For 95%: $Z_{\alpha/4} = Z_{0.05/4} = Z_{0.0125} = 2.24$

An online calculator doing the above: https://science-network.tv/epv-calculator/

For peer review only - http://bmjopen.bmj.com/site/about/guidelines.xhtml

PRISMA 2009 Checklist

Section/topic	#	Checklist item	Reported on page #
**TITLE**			
Title	1	Identify the report as a systematic review, meta-analysis, or both.	1
**ABSTRACT**			
Structured summary	2	Provide a structured summary including, as applicable: background; objectives; data sources; study eligibility criteria, participants, and interventions; study appraisal and synthesis methods; results; limitations; conclusions and implications of key findings; systematic review registration number.	2
**INTRODUCTION**			
Rationale	3	Describe the rationale for the review in the context of what is already known.	4
Objectives	4	Provide an explicit statement of questions being addressed with reference to participants, interventions, comparisons, outcomes, and study design (PICOS).	4
**METHODS**			
Protocol and registration	5	Indicate if a review protocol exists, if and where it can be accessed (e.g., Web address), and, if available, provide registration information including registration number.	4
Eligibility criteria	6	Specify study characteristics (e.g., PICOS, length of follow-up) and report characteristics (e.g., years considered, language, publication status) used as criteria for eligibility, giving rationale.	5
Information sources	7	Describe all information sources (e.g., databases with dates of coverage, contact with study authors to identify additional studies) in the search and date last searched.	5
Search	8	Present full electronic search strategy for at least one database, including any limits used, such that it could be repeated.	31 (Appendix 1)
Study selection	9	State the process for selecting studies (i.e., screening, eligibility, included in systematic review, and, if applicable, included in the meta-analysis).	5
Data collection process	10	Describe method of data extraction from reports (e.g., piloted forms, independently, in duplicate) and any processes for obtaining and confirming data from investigators.	6
Data items	11	List and define all variables for which data were sought (e.g., PICOS, funding sources) and any assumptions and simplifications made.	6
Risk of bias in individual studies	12	Describe methods used for assessing risk of bias of individual studies (including specification of whether this was done at the study or outcome level), and how this information is to be used in any data synthesis.	6 and 26 (figure 2)
Summary measures	13	State the principal summary measures (e.g., risk ratio, difference in means).	6-7

For peer review only - http://bmjopen.bmj.com/site/about/guidelines.xhtml

PRISMA 2009 Checklist

Synthesis of results	14	Describe the methods of handling data and combining results of studies, if done, including measures of consistency (e.g., I^2) for each meta-analysis.	6-7

Section/topic	#	Checklist item	Reported on page #
Risk of bias across studies	15	Specify any assessment of risk of bias that may affect the cumulative evidence (e.g., publication bias, selective reporting within studies).	24 and 26
Additional analyses	16	Describe methods of additional analyses (e.g., sensitivity or subgroup analyses, meta-regression), if done, indicating which were pre-specified.	7 and 27-30
**RESULTS**			
Study selection	17	Give numbers of studies screened, assessed for eligibility, and included in the review, with reasons for exclusions at each stage, ideally with a flow diagram.	25
Study characteristics	18	For each study, present characteristics for which data were extracted (e.g., study size, PICOS, follow-up period) and provide the citations.	24
Risk of bias within studies	19	Present data on risk of bias of each study and, if available, any outcome level assessment (see item 12).	7-10 and 26
Results of individual studies	20	For all outcomes considered (benefits or harms), present, for each study: (a) simple summary data for each intervention group (b) effect estimates and confidence intervals, ideally with a forest plot.	24
Synthesis of results	21	Present results of each meta-analysis done, including confidence intervals and measures of consistency.	10 and 27-30
Risk of bias across studies	22	Present results of any assessment of risk of bias across studies (see Item 15).	9-10 and 26
Additional analysis	23	Give results of additional analyses, if done (e.g., sensitivity or subgroup analyses, meta-regression [see Item 16]).	11 and 27-30
**DISCUSSION**			
Summary of evidence	24	Summarize the main findings including the strength of evidence for each main outcome; consider their relevance to key groups (e.g., healthcare providers, users, and policy makers).	10
Limitations	25	Discuss limitations at study and outcome level (e.g., risk of bias), and at review-level (e.g., incomplete retrieval of identified research, reporting bias).	11-12
Conclusions	26	Provide a general interpretation of the results in the context of other evidence, and implications for future research.	12-13
**FUNDING**			

For peer review only - http://bmjopen.bmj.com/site/about/guidelines.xhtml

PRISMA 2009 Checklist

Funding	27	Describe sources of funding for the systematic review and other support (e.g., supply of data); role of funders for the systematic review.	14

From: Moher D, Liberati A, Tetzlaff J, Altman DG, The PRISMA Group (2009). Preferred Reporting Items for Systematic Reviews and Meta-Analyses: The PRISMA Statement. PLoS Med 6(7): e1000097. doi:10.1371/journal.pmed1000097

For more information, visit: **www.prisma-statement.org**.

For peer review only

For peer review only - http://bmjopen.bmj.com/site/about/guidelines.xhtml

BMJ Open

Acute Sore Throat and Fusobacterium Necrophorum in Primary Health Care – A Systematic Review and Meta-analysis

Journal:	*BMJ Open*
Manuscript ID	bmjopen-2020-042816.R2
Article Type:	Original research
Date Submitted by the Author:	15-Apr-2021
Complete List of Authors:	Malmberg, Stefan; University of Gothenburg Sahlgrenska Academy, School of Public health and Community Medicine<br>Petrén, Susanna; University of Gothenburg Sahlgrenska Academy, School of Public health and Community Medicine<br>Gunnarsson, Ronny; University of Gothenburg Sahlgrenska Academy, School of Public Health and Community Medicine; Vastra Gotalandsregion, Regionhalsan, FoUU<br>Hedin, Katarina; Linköpings universitet Institutionen för medicin och hälsa; Region Jönköpings län, Futurum academy for health and care<br>Sundvall, Pär-Daniel ; Vastra Gotalandsregion, Research and Development Primary Health Care; University of Gothenburg Sahlgrenska Academy, School of Public health and Community Medicine
**Primary Subject Heading**:	General practice / Family practice
Secondary Subject Heading:	Diagnostics, Infectious diseases, Epidemiology
Keywords:	Epidemiology < INFECTIOUS DISEASES, PRIMARY CARE, INFECTIOUS DISEASES, Diagnostic microbiology < INFECTIOUS DISEASES

SCHOLARONE™
Manuscripts

I, the Submitting Author has the right to grant and does grant on behalf of all authors of the Work (as defined in the below author licence), an exclusive licence and/or a non-exclusive licence for contributions from authors who are: i) UK Crown employees; ii) where BMJ has agreed a CC-BY licence shall apply, and/or iii) in accordance with the terms applicable for US Federal Government officers or employees acting as part of their official duties; on a worldwide, perpetual, irrevocable, royalty-free basis to BMJ Publishing Group Ltd ("BMJ") its licensees and where the relevant Journal is co-owned by BMJ to the co-owners of the Journal, to publish the Work in this journal and any other BMJ products and to exploit all rights, as set out in our licence.

The Submitting Author accepts and understands that any supply made under these terms is made by BMJ to the Submitting Author unless you are acting as an employee on behalf of your employer or a postgraduate student of an affiliated institution which is paying any applicable article publishing charge ("APC") for Open Access articles. Where the Submitting Author wishes to make the Work available on an Open Access basis (and intends to pay the relevant APC), the terms of reuse of such Open Access shall be governed by a Creative Commons licence – details of these licences and which Creative Commons licence will apply to this Work are set out in our licence referred to above.

Other than as permitted in any relevant BMJ Author's Self Archiving Policies, I confirm this Work has not been accepted for publication elsewhere, is not being considered for publication elsewhere and does not duplicate material already published. I confirm all authors consent to publication of this Work and authorise the granting of this licence.

For peer review only

Acute Sore Throat and Fusobacterium Necrophorum in PHC – A Systematic Review and Meta-analysis
Version: (15th of April 2021)

**Title**	**Acute Sore Throat and Fusobacterium Necrophorum in Primary Health Care – A Systematic Review and Meta-analysis**
**Authors' name**	Stefan Malmberg[1,2], Susanna Petrén[3], Ronny Gunnarsson[1,4,5], Katarina Hedin[6,7], Pär-Daniel Sundvall[1,4,5]
**Address for each author**	[1] Region Västra Götaland, Research and Development Primary Health Care, Research and Development Centre Södra Älvsborg, Sven Eriksonsplatsen 4, SE-503 38 Borås, Sweden<br><br>[2] HälsoBrunnen VC, Praktikertjänst AB, Vistvägen 2, 52337 Ulricehamn, Sweden.<br><br>[3] Närhälsan Högsbo vårdcentral, Markmyntsgatan 14A, 414 80 Gothenburg, Sweden<br><br>[4] General Practice / Family Medicine, Department of Public Health and Community Medicine, Institute of Medicine, Sahlgrenska Academy, University of Gothenburg, Box 454, SE-405 30 Gothenburg Sweden<br><br>[5] Centre for Antibiotic Resistance Research (CARe) at University of Gothenburg, Gothenburg, Sweden<br><br>[6] Futurum, Region Jönköping County, Jönköping and Department of Health, Medicine and Caring Sciences, Linköping University, Linköping, Sweden<br><br>[7] Department of Clinical Sciences in Malmö, Family Medicine, Lund University, Malmö, Sweden
**Authors' names and positions**	Stefan Malmberg MD PhD student, Susanna Petrén MD, Ronny Gunnarsson MD PhD Professor in General Practice, Katarina Hedin MD PhD and Associate Professor in General Practice, Pär-Daniel Sundvall MD PhD
**Corresponding author**	Stefan Malmberg,<br><br>stefan.malmberg@stfn.se
**Word count**	Abstract: 262<br><br>Manuscript excluding abstract, references and tables: 3,221

For peer review only

For peer review only - http://bmjopen.bmj.com/site/about/guidelines.xhtml

Acute Sore Throat and Fusobacterium Necrophorum in PHC – A Systematic Review and Meta-analysis
Version: (15th of April 2021)

Abstract

Purpose: The main objective of this review was to describe and quantify the association between *Fusobacterium necrophorum* (FN) and acute sore throat in primary health care. **Methods**: In this systematic review and meta-analysis, we searched Scopus and PubMed for case-control studies reporting the prevalence of FN in patients attending primary care for an uncomplicated acute sore throat as well as in healthy controls. Only studies published in English were considered. Publications were not included if they were case studies, or if they included patients prescribed antibiotics before the throat swab, patients with a concurrent malignant disease, on immunosuppression, having an HIV infection, or patients having another acute infection in addition to a sore throat. Inclusion criteria and methods were specified in advance and published in PROSPERO. The primary outcome was positive etiologic predictive value (P-EPV), quantifying the probability for an association between acute sore throat and findings of FN in the pharynx. For comparison, our secondary outcome was the corresponding P-EPV for group A streptococcus (GAS). **Results**: PubMed and Scopus yielded 258 and 232 studies, respectively. Removing duplicates and screening the abstracts resulted in 53 studies subsequently read in full text. For the four studies of medium to high quality included in the meta-analysis, the cumulative P-EPV regarding FN was 64% (95% CI 33-83%). GAS, based on data from the same publications and patients, yielded a positive EPV of 93% (95% CI 83-99%). **Conclusions**: The results indicate that FN may play a role in primary health care patients with an acute sore throat, but the association is much weaker compared to GAS.

For peer review only

Acute Sore Throat and Fusobacterium Necrophorum in PHC – A Systematic Review and Meta-analysis
Version: (15th of April 2021)

Article Summary

Strengths and limitations of this study

- This is the first systematic literature review with meta-analysis utilizing positive etiologic predictive value to quantify the clinical relevance of a finding of *Fusobacterium necrophorum* (FN) in patients presenting with an uncomplicated acute sore throat in PHC.
- The positive etiologic predictive value reveals the probability for a true link between *Fusobacterium necrophorum* and the sore throat expressed as a plain percentage between 0-100% and it was 64% (95% CI 33-83%).
- A potential limitation is that there were only four available case-control studies with low or medium risk for bias presenting the proportion of FN.

For peer review only

For peer review only - http://bmjopen.bmj.com/site/about/guidelines.xhtml

Introduction

An uncomplicated acute sore throat is a common reason for attending primary health care (PHC).[1-3] Most current guidelines concerning the management of patients with a sore throat focus on group A *streptococci* (GAS).[4-6] However, recent studies have indicated that *Fusobacterium necrophorum* (FN) might cause a sore throat, particularly among adolescents and young adults.[7-9]

FN is an anaerobic gram-negative bacterium most known for causing the severe disease Lemierre's syndrome, a potentially life-threatening condition which typically begins with a sore throat, and is also an established pathogen in peritonsillar abscess (PTA).[10-12]

The role of FN in the sore throat has been studied in three recent reviews.[7-9] None of the three reviews have taken into consideration the carriage rate of FN in healthy controls, which is of importance when estimating the clinical relevance of finding FN in patients with an uncomplicated acute sore throat.

This study aimed to estimate the probability for an association between FN and the uncomplicated acute sore throat in patients attending PHC, when taking into consideration the carriage rate of FN in healthy controls. A second aim was to compare the probability for FN with the same probability for an association between GAS and patients with an uncomplicated acute sore throat.

Patient and public involvement

No patients involved.

For peer review only - http://bmjopen.bmj.com/site/about/guidelines.xhtml

Ethics approval

No ethical approval was needed since only publicly available data from published articles (in which informed consent was obtained by the primary investigators) was retrieved and analysed. No personal, sensitive or confidential information was collected in the scope of this study.

Methods

The review was conducted in accordance with the Preferred Reporting Items for Systematic Reviews and Meta-Analyses (PRISMA) statement.[13] Inclusion criteria and methods were specified in advance and documented in the review protocol. The initial protocol was registered and made available beforehand in PROSPERO (International Prospective Register of Systematic Reviews), September 5, 2018 (registration number CRD42018106800).

Search Strategy

PubMed and SCOPUS were searched (17 September 2018) for case-control studies reporting the prevalence of FN in patients with an uncomplicated acute sore throat and in healthy individuals without any signs of infection. There were no time limitations. The search terms are described in Appendix 1.

Study Selection

All case-control studies reporting the prevalence of FN in patients attending a PHC setting for an uncomplicated acute sore throat and in a healthy control group were included. Only studies published in English were considered. Publications were not included if they were case studies, or if they included patients prescribed antibiotics before the throat swab, patients

For peer review only - http://bmjopen.bmj.com/site/about/guidelines.xhtml

with a concurrent malignant disease, on immunosuppression, having an HIV infection, or patients having another acute infection in addition to a sore throat.

SM performed the first screening by reading titles and abstract to remove duplicates from the two search strategies and, thereafter, to remove obviously irrelevant studies such as animal studies. The remaining studies were carefully screened again reading titles and abstracts independently by SM and SP to identify studies that potentially met the inclusion criteria outlined above. SM and SP started screening sitting together in the same room discussing each publication to ensure they aligned their judgement. They then continued screening separately but had a joint discussion whenever they decided differently if a publication should be kept or removed

The full texts of these potentially eligible studies were retrieved and independently assessed for eligibility by SM and SP. Any disagreement between them over the eligibility of particular studies was resolved through discussion within the whole review team.

The reference lists for each article were screened for additional articles potentially matching the inclusion criteria. Such articles were added to the list of potentially eligible studies for further assessment.

Appraisal of methodological quality

SM and SP independently assessed the risk of bias in the included studies by using methodological quality characteristics (Table 1). Overall high quality was defined as having a low risk of bias in all criteria. Having a high risk of bias in any criteria made the study to be of overall low quality. The rest was classified as having an overall moderate risk of bias. Disagreements over the risk of bias in particular studies were resolved by discussion within the review team.

For peer review only - http://bmjopen.bmj.com/site/about/guidelines.xhtml

Data extraction

A standardized, pre-piloted form was used to extract data from the included studies for assessment of methodological quality and evidence synthesis. Extracted information included: study setting, definition of cases and classification of these using the Centor criteria if available, definition of healthy controls, swab method, culture or PCR method, outcomes of throat swabs, and information for the assessment of the risk of bias. SM and SP extracted data independently. Discrepancies were identified and resolved through discussion.

Data analysis

A narrative synthesis was produced for each of the included studies, structured around the study methodology, target population characteristics, outcome, and the assessment of methodological quality.

Studies with a healthy control group and of a medium to high methodological quality were used for the meta-analysis, where the pooled difference in prevalence of FN between cases and healthy controls was compared using chi-square test.

The clinical relevance of any statistical differences between symptomatic patients and healthy controls was further explored by calculating the positive etiologic predictive value (P-EPV)[14]. P-EPV is a method of quantifying the probability of a true link between the symptom (a sore throat) and the finding of FN in the throat while considering the possibility of healthy carriers of FN (Appendix 2). P-EPV for FN was, when possible, compared to P-EPV for GAS using data from the same patients and publications.

Using a random effects meta-analysis would have provided odds ratios for harboring FN among cases compared to controls. The statistical technique we used for meta-analysis, P-EPV, has been used previously[15] and provides a probability for a true connection between

For peer review only - http://bmjopen.bmj.com/site/about/guidelines.xhtml

FN and the symptom of a sore throat in the studied group. This outcome is a plain percent between 0-100%. If a bacterium is found equally often in patients and controls the point estimate of P-EPV will be 0% with a 95% confidence interval from 0.0% and the upper limit will be determined by the sample size. The point estimate of P-EPV will approach 100% when the difference in prevalence of a bacterium between patients and controls increases. This was in our opinion a more clinically useful outcome that can be easily understood, especially by clinicians unfamiliar with research and odds ratios.

P-EPV does not directly take into account between-study variation so it is not a random effect model. We compensate for this by presenting our outcome for each individual study as well as a sensitivity analysis where we compare the consequences of combining them differently. Furthermore, the between-study variation statistics (I^2) calculated in random effect models is very unreliable when the number of included publications is small [16] and we knew already from the start that the number of available publications would be small.

P-EPV has the ability to adjust the proportion of individuals harboring FN between healthy controls and symptomatic carriers ill from something else like a virus. However, in this review we chose to not adjust this, so we set theta to 1.0 meaning that symptomatic carriers harbor FN equally often as healthy controls. Finally, P-EPV allows us to consider the sensitivity of the test to detect FN, something that conventional random effects meta-analysis doesn't.

Results

The PubMed search yielded 258 publications, and the Scopus database query yielded 232 (Figure 1). Reviewing reference lists did not reveal any more relevant publications not found in the initial searches. Removing duplicates and screening the abstracts resulted in 53 studies subsequently read in full text.

For peer review only

For peer review only - http://bmjopen.bmj.com/site/about/guidelines.xhtml

Exclusion of publications

Thirty-seven of these 53 articles were not included because they had a different focus, i.e. lab methods [17], or focused on a different category of patients than was the scope of this review: chronically ill patients [18], hospitalized patients [19-24], or patients with a subset of infections such as peritonsillar abscess, Lemierre's syndrome, chronic/recurrent tonsillitis, and intra-abdominal infections [11 25-52]. Four were excluded because they lacked a control group [53-56]. Discussions in the review team prompted the exclusion of another five articles with methodological limitations in relation to the scope of this review.[57-61]

The article published in 2004 by Aliyu et al.[57] concerned a study where routine throat swabs were analyzed for FN-specific DNA and compared with swabs obtained from healthy adults. The cases were randomly selected in the lab, but it was unclear to what extent they had a sore throat and how these symptoms were registered. The cases included children as young as five months. Hence, it was unclear what kind of patients the routine throat swabs sent to the laboratory represented. Inclusion in the control group required the absence of antibiotic therapy in the preceding two weeks, but not for cases. The swabs from cases were also cultured for GAS, but there was no information about the prevalence of GAS in the control group. The mean age and range differed substantially between cases and controls.

The article published in 2018 by Atkinson et al.[58] presented the results of applying a new laboratory method on swabs from a previously published study [62] and comparing the results to the method that was used initially. The cases included university students complaining of a sore throat, while the controls were asymptomatic students. Only 30 of the 180 control swabs were used in this study, limiting the validity of the comparison between the different lab methods. This limitation is highlighted by the remarkable difference in

For peer review only

For peer review only - http://bmjopen.bmj.com/site/about/guidelines.xhtml

outcome between the laboratory methods when reviewing cases and controls for both FN and GAS.

A Letter to the Editor published in 2014 by Eaton et al. [59] concerned a project in which all throat swabs received by a microbiology laboratory in one year were cultured for GAS and FN, indicating that the only inclusion criterion was that a throat swab was taken. The laboratory served both PHC and secondary care, while the scope of this study was to focus on uncomplicated acute sore throat in PHC. Clinical details stated on accompanying request forms were used to determine if patients had pharyngitis. Those with either persistent or recurrent symptoms were considered to have persistent sore throat syndrome (PSTS), indicating that multiple swabs from the same individual were allowed in the data. There was no information about antibiotic treatment. In conclusion, it was decided not to include the Letter to the Editor by Eaton et al. [59] in this review.

The text published in 2009 by Ludlam et al. [60] described a study design comparing cases from a local GP to controls comprising healthy university students, collecting throat swabs for both groups during the same two-month period. The description of inclusion and exclusion criteria for the cases was limited. The results for GAS, EBV, and FN were not shown for both groups, either in text or in tables. It is unclear whether the controls may become cases (and vice-versa). Their supplementary table contained information about antibiotic treatment in the control group, but not for the cases.

The article published in 2018 by Pallon et al. [61] was a follow-up study based on the same initial data already included in a previously published article by Hedin et al. [63], which is included in this review. Therefore, the article published in 2018 by Pallon et al. [61] was not included.

Methodological quality

For peer review only - http://bmjopen.bmj.com/site/about/guidelines.xhtml

Of the six studies included in the qualitative analysis, three were of overall high quality and one of medium quality [62-65] (Figure 2) and these were included in the meta-analysis.

Two studies presenting data from cases and controls were of low quality [66 67] and these were not included in the meta-analysis for the reasons described below.

The Danish study by Jensen et al. 2015 [67] examined the outcome of throat cultures arriving at a microbiologic laboratory. Most of the cases came from patients with 3-4 Centor criteria who had already tested negative with a rapid antigen detection test (RADT) for GAS. The control group consisted of subjects having a sore throat with 0-2 Centor criteria or fever or who were screened for carriage of Staphylococcus aureus. None of the controls were screened using the above-mentioned RADT. The cases and controls were, therefore, deemed inappropriate for inclusion in the meta-analysis.

The article published in 2007 by Jensen et al. [66] had similar problems as those described above. The inclusion criteria were somewhat unclear. It appears as if primarily patients with a negative outcome of a RADT for GAS were included as cases. Hence, this study was also not included in the meta-analysis.

Presence of Fusobacterium necrophorum in patients with a sore throat

In high or medium quality articles, FN was detected in 18% of cases with a sore throat and a Centor score of 0-4, compared to 7.2% in healthy controls ($p<0.00001$, chi-square) (Table 2). The cumulative positive EPV for FN for the four publications with low or medium risk for bias, including patients with 0-4 Centor scores, was 64% (95% CI 33-83%) (Table 3 and Figure 3). In cases with a Centor score of 3-4, FN was detected in 21% ($p<0.00001$, chi-square) (Table 2). The cumulative positive EPV regarding FN for patients with a Centor score of 3-4 was 71% (95% CI 34-88%) (Table 3 and Figure 4).

For peer review only - http://bmjopen.bmj.com/site/about/guidelines.xhtml

Fusobacterium necrophorum versus group A streptococcus

When including all cases (Centor score 0-4) in studies with low or medium risk for bias also providing data for GAS in the very same patients, the cumulative positive EPV for a finding of GAS was 93% (95% CI 83-99%) (Table 3 and Figure 5). In cases with a Centor score of 3-4, the positive EPV for GAS was 97% (95% CI 91-100%) (Table 3 and Figure 6).

Discussion

This literature review and meta-analysis showed that the P-EPV for FN (detected by culture or PCR) and the uncomplicated acute sore throat was 64% (95% CI 33-83%). The corresponding P-EPV for GAS was 93% (95% CI 83-99%), based on data from the same publications and patients. When limiting the analyses to the patients with Centor score 3-4, the P-EPV for FN was 71% (95% CI 34-88%) and for GAS, 97% (95% CI 91-100%).

Strengths and limitations

A potential limitation is that there were only four available case-control studies presenting the proportion of FN. However, this study is the first systematic literature review with meta-analysis utilizing P-EPV to quantify the clinical relevance of a finding of FN in patients presenting with an acute uncomplicated sore throat in PHC. As such, it represents the current best understanding of the clinical importance of FN in patients with an uncomplicated acute sore throat.

The relatively high prevalence of FN in healthy controls (7.2%) indicates that FN, at least for some patients, is a part of the normal tonsillar flora. The proportion of patients with FN was 18% for Centor score 0-4 and 21% for Centor score 3-4, but the corresponding increase for GAS was from 19% to 35% (Table 2). Subsequently, the difference in the 95% confidence interval for P-EPV between patients having 0-4 versus 3-4 Centor scores was very

For peer review only

For peer review only - http://bmjopen.bmj.com/site/about/guidelines.xhtml

small for FN, even if there was a marginal increase in the point estimate for P-EPV. A larger difference would be expected if FN is the main etiologic agent in a relevant proportion of patients.

The P-EPV numbers indicate that FN plays a role as a pathogen in patients with an uncomplicated acute sore throat. However, compared to the results for GAS, the association between FN and the uncomplicated acute sore throat appears to be considerably weaker and only marginally higher than the P-EPV of 53% (95% CI 36-67%) previously found for adults harboring group C streptococcus. [15] Furthermore, the narrow confidence intervals for GAS P-EPV are in contrast to the wide confidence intervals associated with the P-EPV for FN.

The high P-EPV for a finding of GAS in a throat swab is convincing and confirms the already well-established link between GAS and sore throat.[6]

It has been suggested that antibiotic treatment of uncomplicated acute sore throat caused by FN would be cost-effective if it reduces the incidence of Lemierre's syndrome by at least 20%. [26] However, this has not yet been investigated in a clinical trial, and it is unlikely it ever will be due to the very low incidence of Lemierre's syndrome. Other possible reasons for prescribing antibiotic treatment to patients with an uncomplicated acute sore throat caused by FN might be to shorten symptom duration or reduce the incidence of PTA. However, neither one has ever been tested in a clinical trial. Hence, although theoretically possible, we still have no proof that antibiotic treatment is beneficial to patients with an uncomplicated sore throat and presence of FN.

Conclusions and implications

For uncomplicated acute sore throat in PHC, the confidence interval of P-EPV for FN (33-83%) is wider and lower compared to the corresponding P-EPV for GAS (83-99%). The level of certainty for these confidence intervals are deemed as high as they are based on three high

For peer review only - http://bmjopen.bmj.com/site/about/guidelines.xhtml

quality and one medium quality study including a total of 676 cases and 439 controls. Since the lower limit for the 95% confidence interval for FN is well above 0% we can with a high level of certainty state there is an association between FN and the uncomplicated acute sore throat in PHC. However, it is weaker than the same association for GAS.

We are not aware of any studies showing that antibiotic treatment has beneficial effects on the duration or severity of symptoms in an FN-associated acute sore throat. Nor do we have any evidence that antibiotic treatment to patients with an uncomplicated acute sore throat reduces the incidence of the life threatening Lemierre's syndrome. Hence, in the absence of this evidence we do not recommend routinely searching for FN in throat swabs or prescribing antibiotics to patients with a GAS negative uncomplicated acute sore throat in PHC. However, to our knowledge at least one randomized controlled trial focusing on GAS-negative patients with a sore throat, and analysing the presence of FN, is underway. Hence, our current advice may in the future have to be revised.

More future studies should focus on randomizing patients with an uncomplicated acute sore throat and presence of only FN to treatment with antibiotics or placebo in order to assess whether the treatment is effective to reduce duration and intensity of symptoms, and, more importantly, if complications such as PTA can be prevented. The prevalence of Lemierre's syndrome is so low that any effect of antibiotics on its prevalence most likely would need to be estimated using other designs than a simple clinical trial comparing antibiotics with placebo.

Contributors

RG was responsible for the conception of the idea. The study's overall design was made by RG, SM, PDS, SP and KH. SM and SP were responsible for assessing the eligibility and extract data from publications. Statistical analysis, interpretation of results, and

For peer review only - http://bmjopen.bmj.com/site/about/guidelines.xhtml

writing of the manuscript were made by all authors (RG, SM, PDS, SP and KH). All authors (RG, SM, PDS, SP and KH) approved the final version of the manuscript.

Data sharing statement

The raw data files are available from the corresponding author upon request.

Competing interest statement

None to declare.

Acknowledgements

Funding for this project came from the Local Research and Development Council, Södra Älvsborg, Sweden (reference number VGFOUSA-937533). Funding was also received from Research and Development Center for Primary Health Care, Borås, Sweden (reference number VGFOUSA-P-872381).

The authors would like to thank Professor Robert Centor who willingly provided further information from his study "The clinical presentation of Fusobacterium-positive and streptococcal-positive pharyngitis in a university health clinic: a cross-sectional study" [62], enabling us to calculate separate P-EPV for patients with 0-4 and 3-4 Centor criteria.

For peer review only

References

1. André M, Vernby Å, Odenholt I, et al. Diagnosis-prescribing surveys in 2000, 2002 and 2005 in Swedish general practice: Consultations, diagnosis, diagnostics and treatment choices. *Scandinavian Journal of Infectious Diseases* 2008(40):648- 54. doi: 10.1080/00365540801932439

2. Tyrstrup M, Beckman A, Molstad S, et al. Reduction in antibiotic prescribing for respiratory tract infections in Swedish primary care- a retrospective study of electronic patient records.(Report). *BMC Infectious Diseases* 2016;16(1) doi: 10.1186/s12879-016-2018-9

3. Armstrong GL, Pinner RW. Outpatient visits for infectious diseases in the United States, 1980 through 1996. *Arch Intern Med* 1999;159(21):2531-6. doi: 10.1001/archinte.159.21.2531 [published Online First: 1999/11/26]

4. Shulman ST, Bisno AL, Clegg HW, et al. Clinical practice guideline for the diagnosis and management of group A streptococcal pharyngitis: 2012 update by the Infectious Diseases Society of America. *Clinical infectious diseases : an official publication of the Infectious Diseases Society of America* 2012;55(10):e86. doi: 10.1093/cid/cis629

5. Centor RM, Witherspoon JM, Dalton HP, et al. The diagnosis of strep throat in adults in the emergency room. *Medical decision making : an international journal of the Society for Medical Decision Making* 1981;1(3):239.

6. Pelucchi C, Grigoryan L, Galeone C, et al. Guideline for the management of acute sore throat. Oxford, UK, 2012:1-28.

7. Marchello C, Ebell MH. Prevalence of Group C Streptococcus and Fusobacterium Necrophorum in Patients With Sore Throat: A Meta-Analysis. *Ann Fam Med* 2016;14(6):567-74. doi: 10.1370/afm.2005 [published Online First: 2017/04/05]

For peer review only - http://bmjopen.bmj.com/site/about/guidelines.xhtml

8. Klug TE, Rusan M, Fuursted K, et al. A systematic review of Fusobacterium necrophorum-positive acute tonsillitis: prevalence, methods of detection, patient characteristics, and the usefulness of the Centor score. *European journal of clinical microbiology & infectious diseases : official publication of the European Society of Clinical Microbiology* 2016;35(12):1903-12. doi: 10.1007/s10096-016-2757-y [published Online First: 2016/08/29]

9. Holm K, Bank S, Nielsen H, et al. The role of Fusobacterium necrophorum in pharyngotonsillitis - A review. *Anaerobe* 2016;42:89-97. doi: 10.1016/j.anaerobe.2016.09.006

10. Riordan T. Human infection with Fusobacterium necrophorum (Necrobacillosis), with a focus on Lemierre's syndrome. *Clinical microbiology reviews* 2007;20(4):622-59. doi: 10.1128/cmr.00011-07 [published Online First: 2007/10/16]

11. Wikstén J, Kaltiainen E, Pitkäranta A, et al. Renewal of peritonsillar abscess: Impact of the bacterial species of the infection and clinical features of the patient—A prospective comparative aetiological study. *Clinical Otolaryngology* 2017;42(6):1358-62. doi: 10.1111/coa.12893

12. Powell EL, Powell J, Samuel JR, et al. A review of the pathogenesis of adult peritonsillar abscess: time for a re-evaluation. *Journal of Antimicrobial Chemotherapy* 2013;68(9):1941-50. doi: 10.1093/jac/dkt128

13. Moher D, Liberati A, Tetzlaff J, et al. Preferred reporting items for systematic reviews and meta-analyses: the PRISMA statement. *Annals of internal medicine* 2009;151(4):264. doi: 10.7326/0003-4819-151-4-200908180-00135

14. Gunnarsson RK, Lanke J. The predictive value of microbiologic diagnostic tests if asymptomatic carriers are present. *Statistics in Medicine* 2002;21(12):1773-85. doi: 10.1002/sim.1119

For peer review only - http://bmjopen.bmj.com/site/about/guidelines.xhtml

For peer review only

15. Gunnarsson RK, Manchal N. Group C beta hemolytic Streptococci as a potential pathogen in patients presenting with an uncomplicated acute sore throat - a systematic literature review and meta-analysis. *Scand J Prim Health Care* 2020:1-12. doi: 10.1080/02813432.2020.1753374 [published Online First: 2020/05/05]

16. von Hippel PT. The heterogeneity statistic I(2) can be biased in small meta-analyses. *BMC Med Res Methodol* 2015;15:35. doi: 10.1186/s12874-015-0024-z [published Online First: 2015/04/17]

17. Bank S, Nielsen HM, Hoyer Mathiasen B, et al. Fusobacterium necrophorum- detection and identification on a selective agar. *APMIS* 2010;118(12):994-99. doi: 10.1111/j.1600-0463.2010.02683.x

18. Tejesvi MV, Uhari M, Tapiainen T, et al. Tonsillar microbiota in children with PFAPA (periodic fever, aphthous stomatitis, pharyngitis, and adenitis) syndrome. *European Journal of Clinical Microbiology and Infectious Diseases* 2016;35(6):963-70. doi: 10.1007/s10096-016-2623-y

19. Bailhache M, Mariani-Kurkdjian P, Lehours P, et al. Fusobacterium invasive infections in children: A retrospective study in two French tertiary care centres. *European Journal of Clinical Microbiology and Infectious Diseases* 2013;32(8):1041-47. doi: 10.1007/s10096-013-1848-2

20. Goldenberg NA, Knapp-Clevenger R, Hays T, et al. Lemierre's and Lemierre's-like syndromes in children: survival and thromboembolic outcomes. *Pediatrics* 2005;116(4):e543-8. doi: 10.1542/peds.2005-0433 [published Online First: 2005/09/09]

21. Papaparaskevas J, Katsandri A, Pantazatou A, et al. Epidemiological characteristics of infections caused by Bacteroides, Prevotella and Fusobacterium species: A prospective observational study. *Anaerobe* 2011;17(3):113-17. doi: 10.1016/j.anaerobe.2011.05.013

For peer review only

For peer review only - http://bmjopen.bmj.com/site/about/guidelines.xhtml

22. Ramirez S, Hild TG, Rudolph CN, et al. Increased diagnosis of Lemierre syndrome and other Fusobacterium necrophorum infections at a Children's Hospital. *Pediatrics* 2003;112(5):e380. [published Online First: 2003/11/05]

23. Rusan M, Klug TE, Ovesen T. An overview of the microbiology of acute ear, nose and throat infections requiring hospitalisation. *European journal of clinical microbiology & infectious diseases : official publication of the European Society of Clinical Microbiology* 2009;28(3):243-51. doi: 10.1007/s10096-008-0619-y [published Online First: 2008/10/03]

24. Yusuf E, Halewyck S, Wybo I, et al. Fusobacterium necrophorum and other Fusobacterium spp. isolated from head and neck infections: A 10-year epidemiology study in an academic hospital. *Anaerobe* 2015;34:120-4. doi: 10.1016/j.anaerobe.2015.05.006 [published Online First: 2015/05/20]

25. Albertz N, Nazar G. Peritonsillar abscess: treatment with immediate tonsillectomy - 10 years of experience. *Acta Otolaryngol* 2012;132(10):1102-7. doi: 10.3109/00016489.2012.684399 [published Online First: 2012/06/20]

26. Bank S, Christensen K, Kristensen LH, et al. A cost-effectiveness analysis of identifying Fusobacterium necrophorum in throat swabs followed by antibiotic treatment to reduce the incidence of Lemierre's syndrome and peritonsillar abscesses. *Eur J Clin Microbiol Infect Dis* 2013;32(1):71-8. doi: 10.1007/s10096-012-1715-6

27. Batty A, Wren MW, Gal M. Fusobacterium necrophorum as the cause of recurrent sore throat: comparison of isolates from persistent sore throat syndrome and Lemierre's disease. *J Infect* 2005;51(4):299-306. doi: 10.1016/j.jinf.2004.09.013 [published Online First: 2005/07/30]

For peer review only

28. Batty A, Wren MWD. Prevalence of Fusobacterium necrophorum and other upper respiratory tract pathogens isolated from throat swabs. *British Journal of Biomedical Science* 2005;62(2):66-70. doi: 10.1080/09674845.2005.11732687

29. Bjork H, Bieber L, Hedin K, et al. Tonsillar colonisation of Fusobacterium necrophorum in patients subjected to tonsillectomy. *BMC Infect Dis* 2015;15:264. doi: 10.1186/s12879-015-0975-z [published Online First: 2015/07/15]

30. Brook I. Aerobic and anaerobic bacteriology of peritonsillar abscess in children. *Acta paediatrica Scandinavica* 1981;70(6):831-5. [published Online First: 1981/11/01]

31. Brook I. Role of anaerobic beta-lactamase-producing bacteria in upper respiratory tract infections. *Pediatric Infectious Disease Journal* 1987;6(3):310-16. doi: 10.1097/00006454-198703000-00045

32. Brook I, Foote Jr PA, Slots J. Immune response to anaerobic bacteria in patients with peritonsillar cellulitis and abscess. *Acta Oto-Laryngologica* 1996;116(6):888-91. doi: 10.3109/00016489609137946

33. Brook I, Shah K, Jackson W. Microbiology of healthy and diseased adenoids. *Laryngoscope* 2000;110(6):994-9. doi: 10.1097/00005537-200006000-00021 [published Online First: 2000/06/14]

34. Dapefrid A, Lundström B, Tano K. Prevalence of Fusobacterium necrophorum in tonsils from patients with chronic tonsillitis. *Acta Oto-Laryngologica* 2017;137(3):297-301. doi: 10.1080/00016489.2016.1229024

35. Develioglu ON, Ipek HD, Bahar H, et al. Bacteriological evaluation of tonsillar microbial flora according to age and tonsillar size in recurrent tonsillitis. *European Archives of Oto-Rhino-Laryngology* 2014;271(6):1661-65. doi: 10.1007/s00405-014-2898-5

36. Ehlers Klug T, Rusan M, Fuursted K, et al. Fusobacterium necrophorum: most prevalent pathogen in peritonsillar abscess in Denmark. *Clinical infectious diseases : an official*

For peer review only

For peer review only - http://bmjopen.bmj.com/site/about/guidelines.xhtml

publication of the Infectious Diseases Society of America 2009;49(10):1467-72. doi: 10.1086/644616 [published Online First: 2009/10/22]

37. Hagelskjaer Kristensen L, Prag J. Localised Fusobacterium necrophorum infections: a prospective laboratory-based Danish study. *Eur J Clin Microbiol Infect Dis* 2008;27(8):733-9. doi: 10.1007/s10096-008-0497-3

38. Hagelskjær Kristensen L, Prag J. Lemierre's syndrome and other disseminated Fusobacterium necrophorum infections in Denmark: A prospective epidemiological and clinical survey. *European Journal of Clinical Microbiology and Infectious Diseases* 2008;27(9):779-89. doi: 10.1007/s10096-008-0496-4

39. Hayakawa K, Nagashima M, Ohta K, et al. Fusobacterium necrophorum subsp. funduliforme in tonsils from various patient populations in Japan. *Japanese journal of infectious diseases* 2018 doi: 10.7883/yoken.JJID.2018.061 [published Online First: 2018/06/01]

40. Holm K, Svensson PJ, Rasmussen M. Invasive Fusobacterium necrophorum infections and Lemièrre's syndrome: the role of thrombophilia and EBV. *European Journal of Clinical Microbiology and Infectious Diseases* 2015;34(11):2199-207. doi: 10.1007/s10096-015-2469-8

41. Horn J, Bender BS, Bartlett JG. Role of anaerobic bacteria in perimandibular space infections. *The Annals of otology, rhinology & laryngology Supplement* 1991;154:34-39.

42. Huggan PJ, Murdoch DR. Fusobacterial infections: clinical spectrum and incidence of invasive disease. *J Infect* 2008;57(4):283-9. doi: 10.1016/j.jinf.2008.07.016 [published Online First: 2008/09/23]

43. Jensen A, Fago-Olsen H, Sorensen CH, et al. Molecular mapping to species level of the tonsillar crypt microbiota associated with health and recurrent tonsillitis. *PLoS One*

For peer review only

For peer review only - http://bmjopen.bmj.com/site/about/guidelines.xhtml

2013;8(2):e56418. doi: 10.1371/journal.pone.0056418 [published Online First: 2013/02/26]

44. Jokipii AMM, Jokipii L, Sipila P, et al. Semiquantitative culture results and pathogenic significance of obligate anaerobes in peritonsillar abscesses. *Journal of Clinical Microbiology* 1988;26(5):957-61.

45. Klug TE. Incidence and microbiology of peritonsillar abscess: the influence of season, age, and gender. *European journal of clinical microbiology & infectious diseases : official publication of the European Society of Clinical Microbiology* 2014;33(7):1163-7. doi: 10.1007/s10096-014-2052-8 [published Online First: 2014/01/30]

46. Klug TE, Fischer AS, Antonsen C, et al. Parapharyngeal abscess is frequently associated with concomitant peritonsillar abscess. *Eur Arch Otorhinolaryngol* 2014;271(6):1701-7. doi: 10.1007/s00405-013-2667-x [published Online First: 2013/08/29]

47. Klug TE, Henriksen JJ, Fuursted K, et al. Significant pathogens in peritonsillar abscesses. *European journal of clinical microbiology & infectious diseases : official publication of the European Society of Clinical Microbiology* 2011;30(5):619-27. doi: 10.1007/s10096-010-1130-9 [published Online First: 2010/12/25]

48. Lepelletier D, Pinaud V, Le Conte P, et al. Is there an association between prior anti-inflammatory drug exposure and occurrence of peritonsillar abscess (PTA)? A national multicenter prospective observational case–control study. *European Journal of Clinical Microbiology and Infectious Diseases* 2017;36(1):57-63. doi: 10.1007/s10096-016-2770-1

49. Mitchelmore IJ, Prior AJ, Montgomery PQ, et al. Microbiological features and pathogenesis of peritonsillar abscesses. *European journal of clinical microbiology & infectious diseases : official publication of the European Society of Clinical Microbiology* 1995;14(10):870-7. [published Online First: 1995/10/01]

For peer review only - http://bmjopen.bmj.com/site/about/guidelines.xhtml

50. Price SL, Hardy S, Gale P, et al. Prevalence of Fusobacterium necrophorum in persistent sore throat samples. *Br J Biomed Sci* 2011;68(4):209-10. [published Online First: 2012/01/24]

51. Tachibana T, Orita Y, Takao S, et al. The role of bacteriological studies in the management of peritonsillar abscess. *Auris Nasus Larynx* 2016;43(6):648-53. doi: 10.1016/j.anl.2016.01.009

52. Wiksten JE, Laakso S, Maki M, et al. Microarray identification of bacterial species in peritonsillar abscesses. *European journal of clinical microbiology & infectious diseases : official publication of the European Society of Clinical Microbiology* 2015;34(5):905-11. doi: 10.1007/s10096-014-2301-x [published Online First: 2014/12/31]

53. Amess JA, O'Neill W, Ni Giollariabhaigh C, et al. A six-month audit of the isolation of Fusobacterium necrophorum from patients with sore throat in a district general hospital. *British Journal of Biomedical Science* 2007;64(2):63-65. doi: 10.1080/09674845.2007.11732757

54. Suzuki K, Kurono Y, Ikeda K, et al. Nationwide surveillance of 6 otorhinolaryngological infectious diseases and antimicrobial susceptibility pattern in the isolated pathogens in Japan. *Journal of Infection and Chemotherapy* 2015;21(7):483-91. doi: 10.1016/j.jiac.2015.03.005

55. Van TT, Cox LM, Cox ME, et al. Prevalence of Fusobacterium necrophorum in Children Presenting with Pharyngitis. *J Clin Microbiol* 2017;55(4):1147-53. doi: 10.1128/jcm.02174-16 [published Online First: 2017/01/27]

56. Windfuhr JP, Toepfner N, Steffen G, et al. Clinical practice guideline: tonsillitis I. Diagnostics and nonsurgical management. *European Archives of Oto-Rhino-Laryngology* 2016;273(4):973-87. doi: 10.1007/s00405-015-3872-6

For peer review only

For peer review only - http://bmjopen.bmj.com/site/about/guidelines.xhtml

57. Aliyu SH, Marriott RK, Curran MD, et al. Real-time PCR investigation into the importance of Fusobacterium necrophorum as a cause of acute pharyngitis in general practice. *J Med Microbiol* 2004;53(Pt 10):1029-35. doi: 10.1099/jmm.0.45648-0 [published Online First: 2004/09/11]

58. Atkinson TP, Centor RM, Xiao L, et al. Analysis of the tonsillar microbiome in young adults with sore throat reveals a high relative abundance of Fusobacterium necrophorum with low diversity. *PLoS One* 2018;13(1):e0189423. doi: 10.1371/journal.pone.0189423 [published Online First: 2018/01/20]

59. Eaton C, Swindells J. The significance and epidemiology of Fusobacterium necrophorum in sore throats. *J Infect* 2014;69(2):194-6. doi: 10.1016/j.jinf.2014.03.005 [published Online First: 2014/03/20]

60. Ludlam H, Howard J, Kingston B, et al. Epidemiology of pharyngeal carriage of Fusobacterium necrophorum. *J Med Microbiol* 2009;58(Pt 9):1264-5. doi: 10.1099/jmm.0.006718-0 [published Online First: 2009/06/17]

61. Pallon J, Sundqvist M, Hedin K. A 2-year follow-up study of patients with pharyngotonsillitis. *BMC Infect Dis* 2018;18(1):3. doi: 10.1186/s12879-017-2917-4 [published Online First: 2018/01/03]

62. Centor RM, Atkinson TP, Ratliff AE, et al. The clinical presentation of Fusobacterium-positive and streptococcal-positive pharyngitis in a university health clinic: a cross-sectional study. *Ann Intern Med* 2015;162(4):241-7. doi: 10.7326/m14-1305 [published Online First: 2015/02/17]

63. Hedin K, Bieber L, Lindh M, et al. The aetiology of pharyngotonsillitis in adolescents and adults - Fusobacterium necrophorum is commonly found. *Clinical microbiology and infection : the official publication of the European Society of Clinical Microbiology and*

For peer review only - http://bmjopen.bmj.com/site/about/guidelines.xhtml

Infectious Diseases 2015;21(3):263.e1-7. doi: 10.1016/j.cmi.2014.08.020 [published Online First: 2015/02/07]

64. Kjaerulff AM, Thomsen MK, Ovesen T, et al. Clinical and biochemical characteristics of patients with Fusobacterium necrophorum-positive acute tonsillitis. *Eur Arch Otorhinolaryngol* 2015;272(6):1457-63. doi: 10.1007/s00405-015-3535-7 [published Online First: 2015/04/16]

65. Hayakawa K, Nagashima M, Kanehisa E, et al. Real-time PCR investigation of the prevalence of Fusobacterium necrophorum in patients with pharyngitis in Japan. *J Infect Chemother* 2018;24(12):969-74. doi: 10.1016/j.jiac.2018.09.008 [published Online First: 2018/10/15]

66. Jensen A, Hagelskjær Kristensen L, Prag J. Detection of fusobacterium necrophorum subsp. funduliforme in tonsillitis in young adults by real-time PCR. *Clinical Microbiology and Infection* 2007;13(7):695-701. doi: 10.1111/j.1469-0691.2007.01719.x

67. Jensen A, Hansen TM, Bank S, et al. Fusobacterium necrophorum tonsillitis: an important cause of tonsillitis in adolescents and young adults. *Clinical microbiology and infection : the official publication of the European Society of Clinical Microbiology and Infectious Diseases* 2015;21(3):266.e1-3. doi: 10.1016/j.cmi.2014.09.020 [published Online First: 2015/02/07]

For peer review only

For peer review only - http://bmjopen.bmj.com/site/about/guidelines.xhtml

Table 1- Methodological quality assessment of included studies

	Low risk	Intermediate risk	High risk
**Definition of cases**	Cases well defined as per Centor criteria or similar.	At least two criteria mentioned in case definition.	Cases not defined.
**Healthy controls**	Study includes comparison with asymptomatic controls.	Controls not asymptomatic.	---
**Swab method**	Area of throat swabbed described, transport and storage mentioned.	Area of throat swabbed mentioned but not the transport or storage.	No mention of swab method.
**Culture method**	Clear description of culture media, incubation time (or PCR if used).	Method described but not in detail	Method not discussed
**Type of study**	Case control studies on FN	Community surveillance studies mentioning FN prevalence	Observational studies without well-defined cases and controls
**Same area and time period**	Cases and controls are collected in the same area and time of year	Cases and controls are collected in the same area but over different time periods	Cases and controls are collected in different regions and time periods

For peer review only - http://bmjopen.bmj.com/site/about/guidelines.xhtml

Table 2 – Case-control studies examining Fusobacterium necrophorum and group A streptococcus in patients with an acute uncomplicated sore throat in primary care

Study [ref]	Design	Method	Age, years (range)		Number of cases and controls			% FN detected (number)			% GAS detected (number)		
			Cases	Controls	Cases		Controls	Cases		Controls	Cases		Controls
					Centor 0-4	Centor 3-4		Centor 0-4	Centor 3-4		Centor 0-4	Centor 3-4	
Hedin 2015 [63]	Pro	Culture	33 (15–48)	31 (16-46)	220	85	128	15% (33)	19% (16)	3.1% (4)	30% (66)	49% (42)	2.3% (3)
Centor 2015 [62]	Pro	PCR	22 (15-30)	24 (15-30)	312	64	180	21% (64)	23% (15)	9.4% (17)	10% (32)	16% (10)	1.1% (2)
Kjærulff 2015 [64]	Pro	Culture	28 (15-40)	29 (15-40)	100	29	100	16% (16)	21% (6)	9.0 % (9)	26% (26)	48% (14)	3.0% (3)
Hayakawa 2018 [65]	Pro	PCR + culture	29 (25-37)	33 (26-36)	44	19	31	14% (6)	21% (4)	6.5% (2)	11% (5)	16% (3)	0.0% (0)
SUBTOTAL (low & medium RISK for bias)					***676***	***197***	***439***	***18% (119)***	***21% (41)***	***7.2% (32)***	***19% (129)***	***35% (69)***	***1.8(8)***
Jensen 2007 [66]	Pro	PCR + culture	25 (18-32)	22 (18-32)	105	-	92	51% (54)	-	21% (19)	5.7%* (6*)	-	-
Jensen 2015 [67]	Retro	Culture	19 (10-40)	22 (10-40)	179	-	176	24% (43)	-	5.7% (10)	3.9%* (7*)	-	0% (0)
SUBTOTAL (high risk for bias)					*284*	-	*268*	*34% (97)*	-	*11% (29)*	*4.6%* (13*)*	-	-
TOTAL (ALL SIX ARTICLES)					960	178	707	23% (216)	21% (37)	8.6% (61)	15%* (142*)	39% (69)	1.1% (8)

*GAS-tonsillitis was excluded (by GPs using rapid antigen tests) in the two articles by Jensen et al.; thus, those results for GAS were irrelevant for the purpose of this meta-analysis.

For peer review only

Table 3 – Positive etiologic predictive value (P-EPV)*

Study	Fusobacterium necrophorum Centor 0-4**	Fusobacterium necrophorum Centor 3-4**	Group A streptococcus Centor 0-4**	Group A streptococcus Centor 3-4**
Hedin 2015 (Sweden)	82% (34-100)	86% (32-100)	95% (82-100)	98% (91-100)
Centor 2015 (US)	60% (8.2-87)	67% (0.0-92)	90% (57-100)	94% (49-100)
Kjaerulff 2015 Denmark)	49% (0.0-92)	63% (0.0-96)	93% (71-100)	97% (81-100)
Hayakawa 2018 (Japan)	57% (0.0-100)	76% (0.0-100)	-----	-----
All studies combined***	64% (33-83)	71% (34-88)	93% (83-99)	97% (91-100)

* P-EPV is a method of quantifying the probability of a true link between symptoms and signs (a sore throat) and the finding of a bacterium in the throat while considering the possibility of healthy carriers of the same bacterium. 0% indicates no probability for a true link and 100% indicates a certain link.

** Including either all patients with a sore throat (Centor criteria 0-4) or only those with more prominent symptoms (Centor score 3-4).

*** Only studies with low or medium risk for bias are included.

For peer review only

For peer review only - http://bmjopen.bmj.com/site/about/guidelines.xhtml

Figure 1 – PRISMA Flow Diagram

Figure 2 – Quality assessment of included studies

Figure 3 - Probability of a true link between sore throat with Centor score 0-4 and positive FN test

*Positive etiologic predictive value is the probability of a true link between sore throat and FN based on studies with data from both patients and healthy controls.

Figure 4 - Probability of a true link between the sore throat with Centor score 3-4 and positive FN test

*Positive etiologic predictive value is the probability of a true link between sore throat and FN based on studies with data from both patients and healthy controls.

For peer review only - http://bmjopen.bmj.com/site/about/guidelines.xhtml

Figure 5 - Probability of a true link between sore throat with Centor score 0-4 and positive GAS test

*Positive etiologic predictive value is the probability of a true link between sore throat and GAS based on studies with data from both patients and healthy controls.

Since the study by Hayakawa 2018 found no positive tests for GAS among healthy controls, P-EPV could not be calculated for that study separately. However, the results from the four studies selected for meta-analysis are included in the summary.

Figure 6 - Probability of a true link between sore throat with Centor score 3-4 and positive GAS test

*Positive etiologic predictive value is the probability of a true link between sore throat and GAS based on studies with data from both patients and healthy controls.
Since the study by Hayakawa 2018 found no positive tests for GAS among healthy controls, P-EPV could not be calculated for that study separately. However, the results from the four studies selected for meta-analysis are included in the summary.

For peer review only

For peer review only - http://bmjopen.bmj.com/site/about/guidelines.xhtml

Acute Sore Throat and Fusobacterium Necrophorum in PHC – A Systematic Review and Meta-analysis
Version: (15/7/20 12:12:00 pm)

PRISMA 2009 Flow Diagram

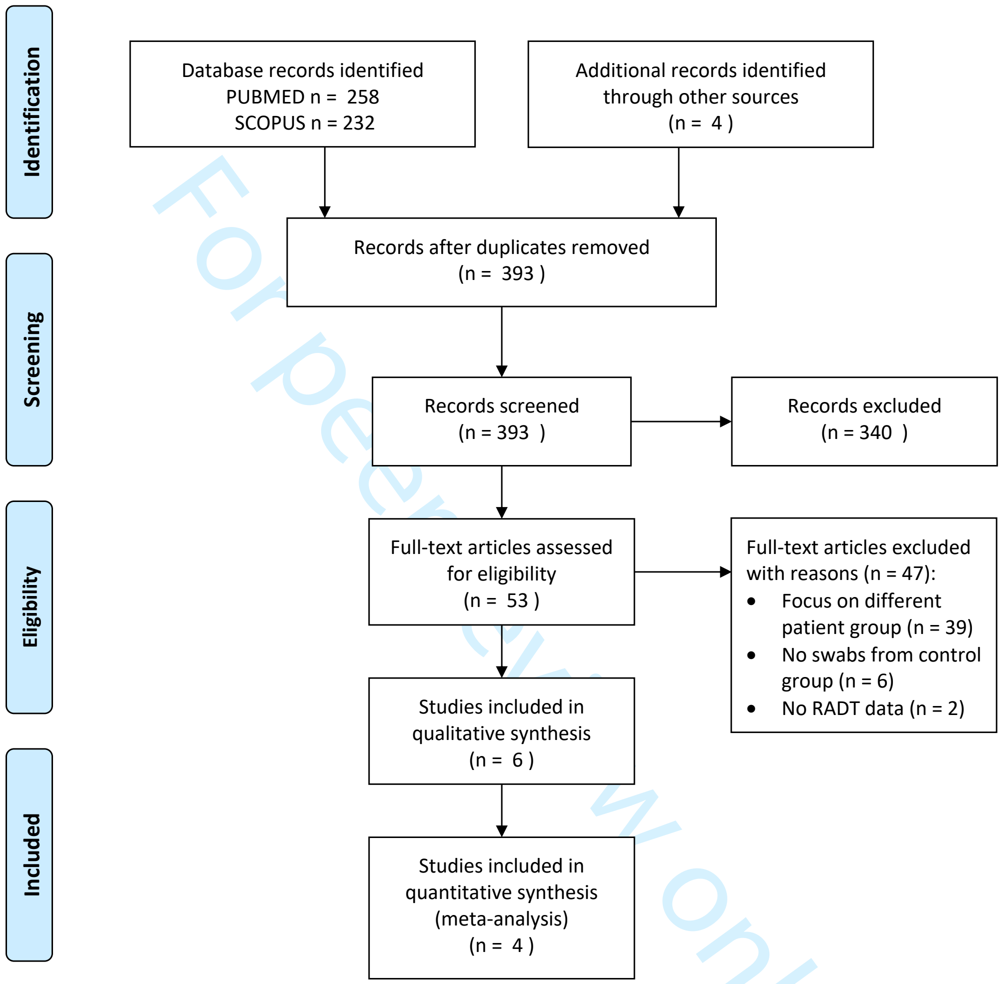

From: Moher D, Liberati A, Tetzlaff J, Altman DG, The PRISMA Group (2009). *P*referred *R*eporting *I*tems for *S*ystematic Reviews and *M*eta-*A*nalyses: The PRISMA Statement. PLoS Med 6(7): e1000097. doi:10.1371/journal.pmed1000097

For more information, visit www.prisma-statement.org.

Figure 1- PRISMA Flow Diagram

For peer review only

For peer review only - http://bmjopen.bmj.com/site/about/guidelines.xhtml

Study	Case defined	Healthy controls	Swab method	Culture method	Type of study	Same geo-graphical area, time
Hedin, 2015	+ (green)	+ (green)	+ (green)	+ (green)	+ (green)	+ (green)
Centor, 2015	+ (green)	+ (green)	+ (green)	+ (green)	+ (green)	+ (green)
Kjærulf, 2015	+ (green)	+ (green)	+ (green)	+ (green)	+ (green)	+ (green)
Hayakawa, 2018	+ (orange)	+ (green)	+ (green)	+ (green)	+ (green)	+ (green)
Jensen, 2007	+ (orange)	+ (green)	+ (red)	+ (green)	+ (green)	+ (orange)
Jensen, 2015	+ (green)	+ (orange)	+ (red)	+ (orange)	+ (red)	+ (green)

Figure 2 - Quality assessment of included studies

+ (green) Low risk of bias + (orange) Intermediate risk of bias + (red) High risk of bias

For peer review only

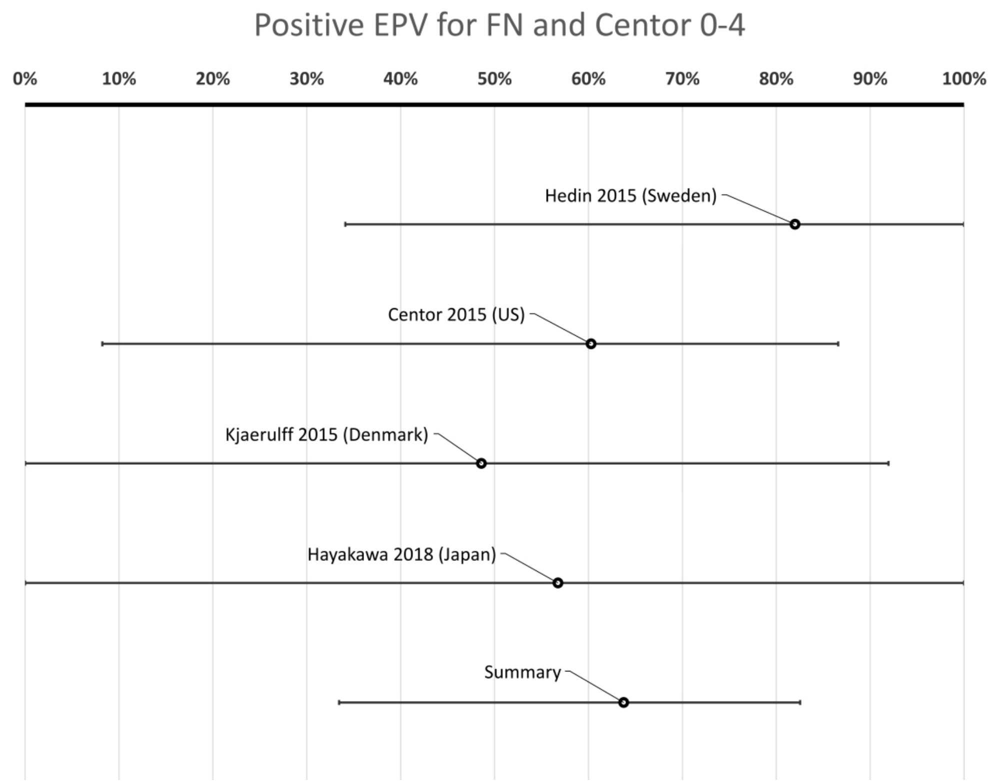

Figure 3 - Probability of a true link between sore throat with Centor score 0-4 and positive FN test

176x137mm (600 x 600 DPI)

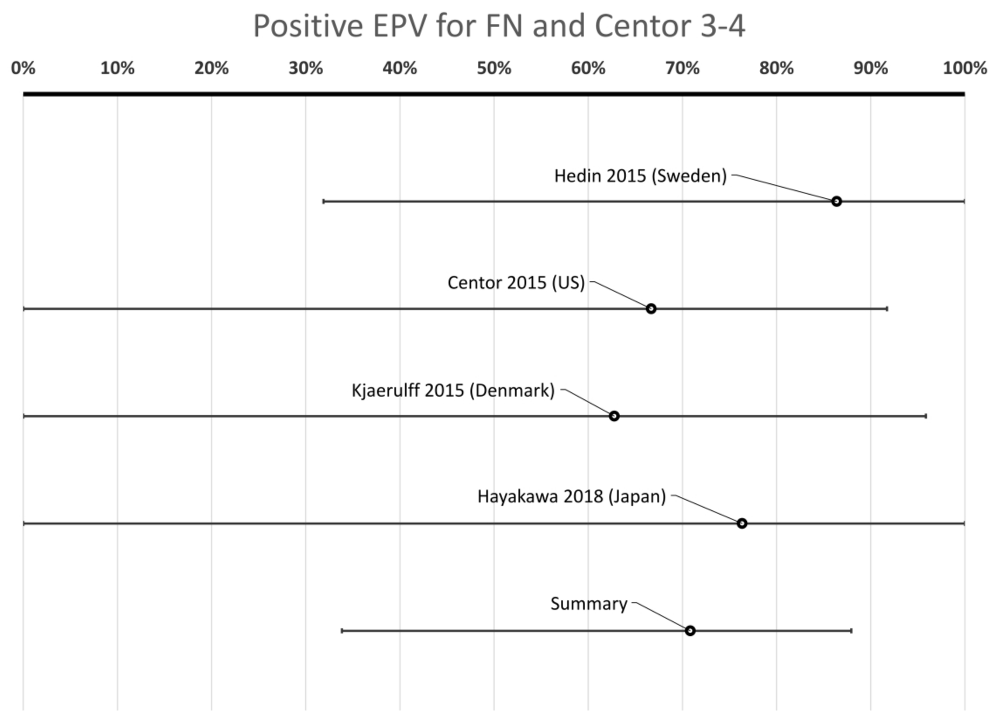

Figure 4 - Probability of a true link between the sore throat with Centor score 3-4 and positive FN test

175x125mm (600 x 600 DPI)

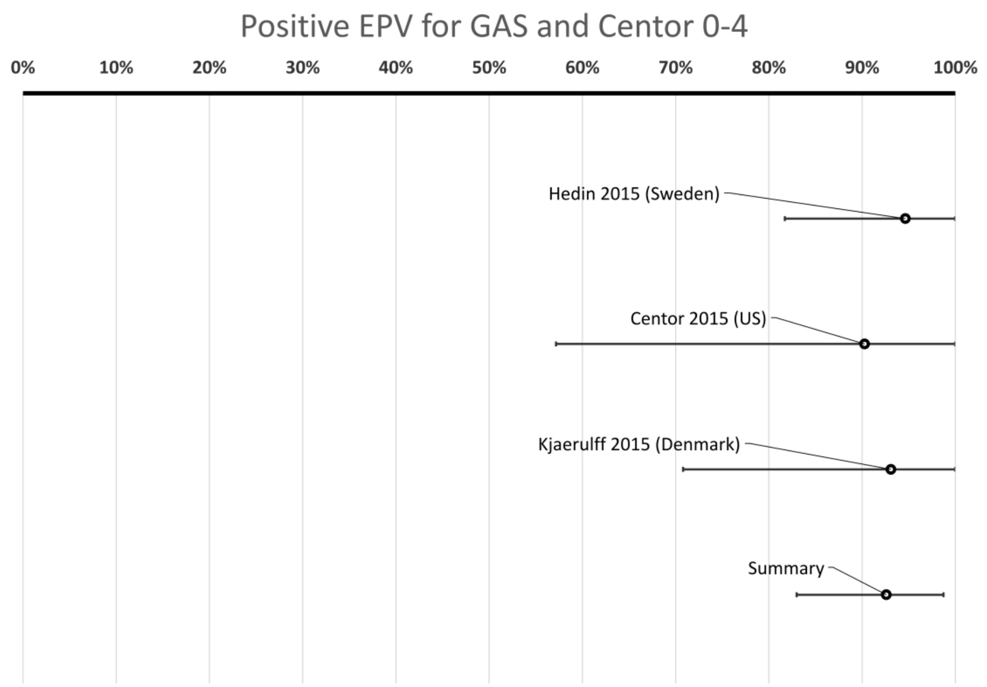

Figure 5 - Probability of a true link between sore throat with Centor score 0-4 and positive GAS test

178x121mm (600 x 600 DPI)

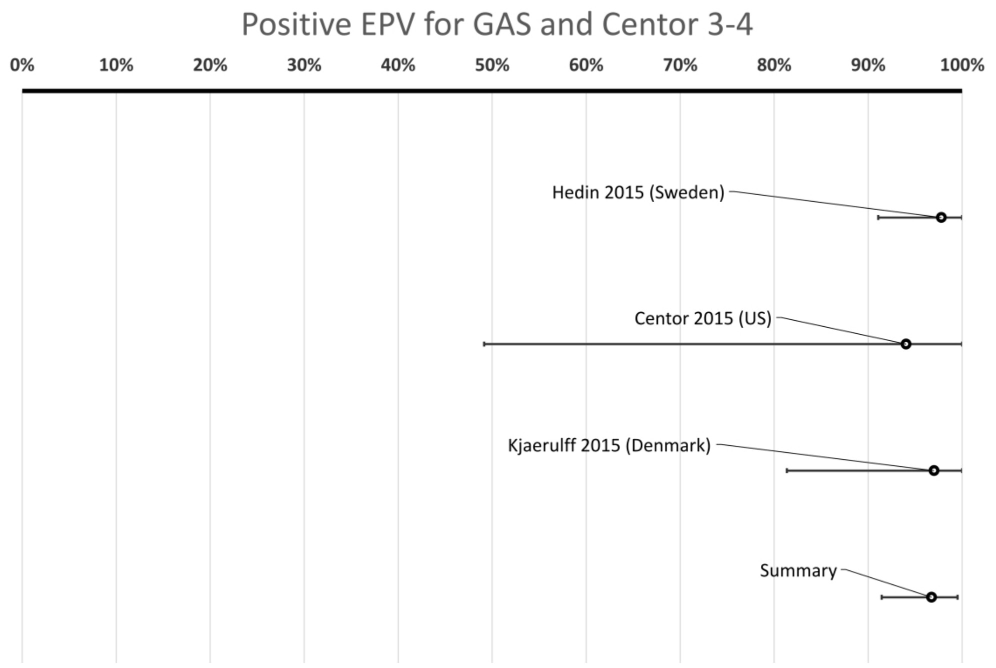

Figure 6 - Probability of a true link between sore throat with Centor score 3-4 and positive GAS test

176x117mm (600 x 600 DPI)

Acute Sore Throat and Fusobacterium Necrophorum in PHC – A Systematic Review and Meta-analysis - Appendix 1 and 2
Version: (11 January 2021)

Appendix 1: Search strings for PubMed and PROSPERO

PubMed:

(("pharyngitis"[MH] OR "pharyngitis"[TIAB] OR "Lemierre Syndrome"[MH] OR "Lemierre Syndrome"[TIAB] OR "Necrobacillosis"[TIAB] OR "pharyngotonsillitis "[TIAB] OR "tonsillitits"[TIAB] OR "throat"[TIAB] OR "epidemiology"[TIAB])

AND ("fusobacterium necrophorum"[MH] OR "fusobacterium"[TIAB]))

NOT "Case Reports"[pt]

Scopus:

(TITLE-ABS-KEY ((pharyngitis OR "Lemierre Syndrome" OR necrobacillosis OR pharyngotonsillitis OR tonsillitis OR "Peritonsillar Abscess" OR throat OR epidemiology))

AND TITLE-ABS-KEY (fusobacterium))

AND DOCTYPE (ar)

AND (LIMIT-TO (SUBJAREA , "MEDI ") OR LIMIT-TO (SUBJAREA , " IMMU ")

OR LIMIT-TO (SUBJAREA , " BIO "))

AND (LIMIT-TO (LANGUAGE , "English "))

AND (EXCLUDE (EXACTKEYWORD , "Case Report"))

For peer review only - http://bmjopen.bmj.com/site/about/guidelines.xhtml

Acute Sore Throat and Fusobacterium Necrophorum in PHC – A Systematic Review and Meta-analysis - Appendix 1 and 2
Version: (11 January 2021)

Appendix 2: Formulae for calculating P-EPV

Formula for the point estimate of P-EPV:

$$P(\mathrm{D}^+|\mathrm{S}^+\mathrm{T}^+) = 1 - \left(\frac{\frac{\mathrm{Sen}}{P(\mathrm{T}^+|\mathrm{S}^+)} - 1}{\frac{\mathrm{Sen}}{P(\mathrm{T}^+|\mathrm{S}^+\mathrm{D}^-)} - 1} \right)$$

Formula for the confidence interval of P-EPV:

$$\left(1 - \left(\frac{\frac{\mathrm{Sen}}{a} - 1}{\frac{\mathrm{Sen}}{d} - 1} \right) \right), \left(1 - \left(\frac{\frac{\mathrm{Sen}}{b} - 1}{\frac{\mathrm{Sen}}{c} - 1} \right) \right)$$

$$a = p(\mathrm{T}^+|\mathrm{S}^+) - \left(Z_{\alpha/4} \times \sqrt{\frac{p(\mathrm{T}^+|\mathrm{S}^+) \times (1 - p(\mathrm{T}^+|\mathrm{S}^+))}{\#(\mathrm{S}^+)}} \right)$$

$$b = p(\mathrm{T}^+|\mathrm{S}^+) + \left(Z_{\alpha/4} \times \sqrt{\frac{p(\mathrm{T}^+|\mathrm{S}^+) \times (1 - p(\mathrm{T}^+|\mathrm{S}^+))}{\#(\mathrm{S}^+)}} \right)$$

$$c = p(\mathrm{T}^+|\mathrm{S}^+\mathrm{D}^-) - \left(Z_{\alpha/4} \times \theta \times \sqrt{\frac{p(\mathrm{T}^+|\mathrm{S}^-) \times (1 - p(\mathrm{T}^+|\mathrm{S}^-))}{\#(\mathrm{S}^-)}} \right)$$

$$d = p(\mathrm{T}^+|\mathrm{S}^+\mathrm{D}^-) + \left(Z_{\alpha/4} \times \theta \times \sqrt{\frac{p(\mathrm{T}^+|\mathrm{S}^-) \times (1 - p(\mathrm{T}^+|\mathrm{S}^-))}{\#(\mathrm{S}^-)}} \right)$$

Definitions:
T = Test outcome which may be positive (T^+) or negative (T^-)
S = Symptoms of a sore throat which may be present (S^+) or absent (S^-)
M = The bacteria of interest (FN or GAS) which may be present (M^+) or absent (M^-)
D = Disease (a sore throat caused by FN/GAS) which may be present (D^+) or absent (D^-)
Sensitivity of test to detect bacteria = $\mathrm{Sen} = P(\mathrm{T}^+|\mathrm{M}^+)$

Setting the width of the confidence interval. For 95%: $Z_{\alpha/4} = Z_{0.05/4} = Z_{0.0125} = 2.24$

An online calculator doing the above: https://science-network.tv/epv-calculator/

For peer review only - http://bmjopen.bmj.com/site/about/guidelines.xhtml

PRISMA 2009 Checklist

Section/topic	#	Checklist item	Reported on page #
**TITLE**			
Title	1	Identify the report as a systematic review, meta-analysis, or both.	1
**ABSTRACT**			
Structured summary	2	Provide a structured summary including, as applicable: background; objectives; data sources; study eligibility criteria, participants, and interventions; study appraisal and synthesis methods; results; limitations; conclusions and implications of key findings; systematic review registration number.	2
**INTRODUCTION**			
Rationale	3	Describe the rationale for the review in the context of what is already known.	4
Objectives	4	Provide an explicit statement of questions being addressed with reference to participants, interventions, comparisons, outcomes, and study design (PICOS).	4
**METHODS**			
Protocol and registration	5	Indicate if a review protocol exists, if and where it can be accessed (e.g., Web address), and, if available, provide registration information including registration number.	4
Eligibility criteria	6	Specify study characteristics (e.g., PICOS, length of follow-up) and report characteristics (e.g., years considered, language, publication status) used as criteria for eligibility, giving rationale.	5
Information sources	7	Describe all information sources (e.g., databases with dates of coverage, contact with study authors to identify additional studies) in the search and date last searched.	5
Search	8	Present full electronic search strategy for at least one database, including any limits used, such that it could be repeated.	31 (Appendix 1)
Study selection	9	State the process for selecting studies (i.e., screening, eligibility, included in systematic review, and, if applicable, included in the meta-analysis).	5
Data collection process	10	Describe method of data extraction from reports (e.g., piloted forms, independently, in duplicate) and any processes for obtaining and confirming data from investigators.	6
Data items	11	List and define all variables for which data were sought (e.g., PICOS, funding sources) and any assumptions and simplifications made.	6
Risk of bias in individual studies	12	Describe methods used for assessing risk of bias of individual studies (including specification of whether this was done at the study or outcome level), and how this information is to be used in any data synthesis.	6 and 26 (figure 2)
Summary measures	13	State the principal summary measures (e.g., risk ratio, difference in means).	6-7

For peer review only - http://bmjopen.bmj.com/site/about/guidelines.xhtml

PRISMA 2009 Checklist

Synthesis of results	14	Describe the methods of handling data and combining results of studies, if done, including measures of consistency (e.g., I^2) for each meta-analysis.	6-7

Section/topic	#	Checklist item	Reported on page #
Risk of bias across studies	15	Specify any assessment of risk of bias that may affect the cumulative evidence (e.g., publication bias, selective reporting within studies).	24 and 26
Additional analyses	16	Describe methods of additional analyses (e.g., sensitivity or subgroup analyses, meta-regression), if done, indicating which were pre-specified.	7 and 27-30
**RESULTS**			
Study selection	17	Give numbers of studies screened, assessed for eligibility, and included in the review, with reasons for exclusions at each stage, ideally with a flow diagram.	25
Study characteristics	18	For each study, present characteristics for which data were extracted (e.g., study size, PICOS, follow-up period) and provide the citations.	24
Risk of bias within studies	19	Present data on risk of bias of each study and, if available, any outcome level assessment (see item 12).	7-10 and 26
Results of individual studies	20	For all outcomes considered (benefits or harms), present, for each study: (a) simple summary data for each intervention group (b) effect estimates and confidence intervals, ideally with a forest plot.	24
Synthesis of results	21	Present results of each meta-analysis done, including confidence intervals and measures of consistency.	10 and 27-30
Risk of bias across studies	22	Present results of any assessment of risk of bias across studies (see Item 15).	9-10 and 26
Additional analysis	23	Give results of additional analyses, if done (e.g., sensitivity or subgroup analyses, meta-regression [see Item 16]).	11 and 27-30
**DISCUSSION**			
Summary of evidence	24	Summarize the main findings including the strength of evidence for each main outcome; consider their relevance to key groups (e.g., healthcare providers, users, and policy makers).	10
Limitations	25	Discuss limitations at study and outcome level (e.g., risk of bias), and at review-level (e.g., incomplete retrieval of identified research, reporting bias).	11-12
Conclusions	26	Provide a general interpretation of the results in the context of other evidence, and implications for future research.	12-13
**FUNDING**			

For peer review only

PRISMA 2009 Checklist

Funding	27	Describe sources of funding for the systematic review and other support (e.g., supply of data); role of funders for the systematic review.	14

From: Moher D, Liberati A, Tetzlaff J, Altman DG, The PRISMA Group (2009). Preferred Reporting Items for Systematic Reviews and Meta-Analyses: The PRISMA Statement. PLoS Med 6(7): e1000097. doi:10.1371/journal.pmed1000097

For more information, visit: **www.prisma-statement.org**.

For peer review only

For peer review only - http://bmjopen.bmj.com/site/about/guidelines.xhtml
